# Expanded roles of community health workers beyond malaria in the Asia-Pacific: A systematic review

**DOI:** 10.1371/journal.pgph.0003113

**Published:** 2024-10-16

**Authors:** Monnaphat Jongdeepaisal, Panarasri Khonputsa, Massaya Sirimatayanant, Worarat Khuenpetch, Elinor Harriss, Richard J. Maude

**Affiliations:** 1 Mahidol Oxford Tropical Medicine Research Unit, Faculty of Tropical Medicine, Mahidol University, Bangkok, Thailand; 2 Centre for Tropical Medicine and Global Health, Nuffield Department of Medicine, University of Oxford, Oxford, United Kingdom; 3 Bodleian Health Care Libraries, University of Oxford, Oxford, United Kingdom; 4 Harvard TH Chan School of Public Health, Harvard University, Boston, Massachusetts, United States of America; 5 The Open University, Milton Keynes, United Kingdom; UP Manila: University of the Philippines Manila, PHILIPPINES

## Abstract

In the Greater Mekong Subregion (GMS), community health workers (CHWs) are a key component of malaria elimination strategies. As malaria declines, support for, and uptake of, malaria services may also subsequently decrease. Expanding their roles beyond malaria has been proposed to sustain the services. A systematic review was conducted to identify and characterize programmes with CHWs providing services in addition to those for malaria in the Asia Pacific. This review describes the expanded roles, identifies evidence of impact or success of the programmes, and explores strategies to ensure sustainability and factors for effective implementation to inform the design of malaria CHW programmes. Searches were conducted in 6 databases, for grey literature, and in bibliographies of retrieved articles. Data were extracted from 38 published articles, 12 programme reports, and 4 programme briefs and analysed using thematic coding and descriptive analysis. Twenty-nine programmes were identified with CHWs performing both malaria and non-malaria roles in the Asia Pacific. There was evidence of impact on malaria incidence in 4 of these, none on malaria mortality, and 4 on other diseases. Monitoring and evaluation mechanisms, multi-sectoral stakeholder collaborations, and adequate training and consistent supervision of CHWs were key to effective programme implementation. Integration of programmes into broader health services, ongoing political and funding support, and engagement with local communities were found to contribute to sustaining provision of health services by CHWs. Expanding CHW roles depends on programme management and strengthening linkages with local health systems. To sustain malaria CHW services, countries need adequate policies and financing, and sufficiently strong health systems to deliver basic health services that are adapted to the health needs of the community which means transitioning away from vertical disease programs. Further research should explore programmes that have not been captured in this review and address gaps in measuring malaria outcomes.

## Introduction

Community-based lay health workers also known as “Community Health Workers” (CHWs) have been recognised for their capacity to enhance access to, and quality of, care delivery in resource-limited settings [1]. A 2010 World Health Organization (WHO) report promoted a wide range of services provided by CHWs from infectious disease prevention to maternal and child health services in remote communities where health professionals are insufficient [2]. CHWs also provide people-centered services such as care coordination, health coaching, and social support [3]. In the GMS, large CHW networks such as the Village Health Volunteers (VHVs) in Thailand [4] and Lao PDR [5] have long been leveraged to provide health information and services in their communities to support the Primary Health Care Systems to deliver decentralized health services in alignment with the Declaration of Alma Ata in 1978.

CHWs have also become a key component of malaria elimination strategies in the GMS region. These frontline health workers, often referred to as Village Malaria Workers (VMWs), can provide malaria testing, treatment, surveillance, and other prevention and control interventions to local communities in remote areas. While well accepted, a limitation to this strategy could be the decreasing uptake of malaria services as malaria declines. Countries approaching elimination may also find it difficult to justify ongoing investment in these workers to detect and treat the few remaining cases, and the programmes may be scaled back [6, 7]. From 2000–2022, there has been a 77% decrease in estimated malaria cases from 22.8 to 5.2 million in the South-East Asia Region, accounting for 2% of the global malaria burden in 2022 [8]. The Asia-Pacific region aims to eliminate all malaria by 2030 [9], and as countries approach the elimination phase, there is an increasing urgency to promptly detect and treat all malaria cases with effective antimalarials to prevent onward transmission in the community [10]; CHWs have been leveraged to support these efforts [11].

Much of the literature on CHWs has focused on factors affecting CHW and programme performance [12], yet there is limited research on effectiveness of CHW integrated interventions in addressing different health issues, including malaria. The currently recommended malaria integration package draws heavily on findings from high endemic countries in Sub-Saharan Africa [13–16]. In 2012, the WHO has recommended that CHWs provide integrated community case management (iCCM) for malaria, pneumonia, diarrhoea, and malnutrition, to children under five [17]. While iCCM is recognised across the region as an effective strategy to reduce child mortality and can maintain the quality of malaria services with additional roles [14, 18, 19], various implementation challenges have been identified in a 2017 review of programmes in Sub-Saharan Africa and Asia [20]. Heterogeneity of community delivered models and their respective impacts on health outcomes makes it difficult to generalize findings from the African context to low transmission and elimination settings [10]. Furthermore, there is also a lack of evidence for iCCM impact on disease morbidity and mortality [13, 21].

More recently, a 2021 WHO guidance on health policy and system support to optimize CHW programmes for HIV, tuberculosis and malaria services specified the need for further research regarding the contextual factors, enablers for effective implementation, and implications of implementing multiple interventions simultaneously [22]. Additionally, the question remains whether expanding malaria CHWs roles can lead to sustaining malaria services in the GMS context.

In light of limited evidence on malaria CHWs expanded roles beyond malaria, this systematic review was conducted to identify and characterise malaria CHW programmes in the Asia Pacific that have expanded CHWs’ role beyond malaria. The aim is to answer the following: 1. What expanded roles beyond providing malaria services do CHWs perform in the Asia-Pacific region? 2. What evidence is there for the outcomes or impact of each programme? 3. What strategies are used to ensure sustainability?

## Methodology

A systematic review was conducted following the Preferred Reporting Items for Systematic Reviews and Meta-Analyses (PRISMA) guidelines. The protocol was registered on PROSPERO (CRD42021250639) [23]. Using insights from 2 published systematic reviews [12, 24], the primary outcomes of interest were derived from the three main review questions (**Table 1**).

**Table 1:** Outcomes of interest.

**Outcome 1**	Expanded roles of malaria CHWs	◾ Identification of malaria services provided by CHWs ◾ Identification of non-malaria services provided by CHWs
**Outcome 2**	Evidence of outcomes or impact of the CHW programme	◾ Malaria incidence (number and/or percentage of people diagnosed with malaria by microscopy, rapid diagnostic test (RDT), or polymerase chain reaction (PCR)) ◾ Malaria mortality ◾ Other disease incidence or prevalence as diagnosed by formal healthcare providers ◾ Other disease mortality as reported in the health information system or government reports ◾ Change in measures of service uptake of malaria delivered by CHWs (including but not limited to consultations, diagnoses, treatments, and referrals) ◾ Measures of service uptake of other diseases delivered by CHWs (including but not limited to consultations, screening, treatments, and referrals)
**Outcome 3**	Strategies to ensure sustainability and factors for effective implementation	◾ Quantitative and/or qualitative measures of community engagement activities (e.g. CHW acceptability by communities and stakeholders, job satisfaction, and motivation) ◾ Quantitative and/or qualitative measures of stakeholder collaborations ◾ Quantitative and/or qualitative measures of integration into the wider health system ◾ Quantitative and/or qualitative measures of training and supervision of CHWs ◾ Quantitative and/or qualitative measures of sources of funding and supply/materials I◾ dentification of barriers to effective implementation ◾ Identification of facilitators of effective implementation

### Definition of CHWs

This review follows and expands on the WHO definition of CHWs as a group of healthcare workers selected by, or working in, their communities, and may receive training to perform specific health interventions and/or roles related to healthcare delivery [25]. The CHW terminology embraces a broader group or cadres of health workers beyond VMWs present in different Asia-Pacific countries. This is intended to accommodate identification of CHW groups with different names that exist within the same country, and the varying combinations of roles they may perform within their communities. In this review “malaria CHWs” is used to refer to those who provide malaria and non-malaria services.

### Search strategy

An information specialist (EH) searched the following databases from inception to the final search date (26/02/2024): Ovid Medline; Ovid Embase; Ovid Global Health; Cochrane Central Register of Controlled Trials; WHO Global Index Medicus; and PubMed. No limitations were applied. The search strategies used text words, acronyms, phrases, and relevant indexing to capture studies about the expanded roles of CHWs beyond malaria services in the Asia-Pacific, using the list of 58 countries [26]. Additional references were retrieved from Google Scholar using search strings adapted from the original search strategy. All references were exported to Endnote X21 (Thomson Reuters, New York, NY), and duplicates were removed [27]. In addition to the above databases, web searches were conducted for grey literature on organisations working with malaria programmes in the Asia Pacific, including reports from government and non-government agencies. Reference lists of included papers were also assessed for additional relevant studies.

### Eligibility criteria, screening, and article selection

Studies were selected for inclusion based on fitness for purpose rather than following a hierarchy of evidence. This review, therefore, considered any literature which met the following inclusion criteria:

1. Identify CHWs or related cadre roles, AND
2. Focus on malaria programme(s) in Asia-Pacific countries, AND
3. Identify expanded roles of CHW or related cadres beyond malaria services, AND
4. Provide analysis of outcomes or impact of the CHW programme on malaria, AND/OR
5. Provide information on sustainability of the CHW programme, AND/OR
6. Are qualitative studies, case studies, process evaluations, and/or cost-effectiveness studies that identify barriers to, and/or facilitators of, effective implementation.

Screened literature ranged from published peer-reviewed primary research with no restriction on study design and methodology, to grey literature including unpublished reports, evaluations, and project briefs. If a systematic review was identified for inclusion, the reference list will be screened for papers that may fulfil the inclusion criteria. Only items written in English were included. This review excluded conference abstracts, clinical trial registration, review or opinion papers, and publications where full text could not be acquired or only reported activities outside of the Asia Pacific.

Deduplicated lists of article titles and abstracts were divided and assigned for independent review among two review teams (PK and WK, and MS and MJ) for evaluation. Reconciliation regarding diverging opinions on eligibility was done against the inclusion criteria within the individual teams through discussion. Disagreements within either review team were resolved by consulting a third reviewer from the other team; if the disagreement persisted, a fourth reviewer’s opinion (RJM) was sought. Full-text articles and grey literature underwent a final screening for eligibility.

### Data extraction and management

Included articles were divided between the two review teams for data extraction which was done for each article by both reviewers in each team. The accuracy and correctness of information extracted by each reviewer was reconciled within each review team. A pre-designed data extraction form was developed and piloted through joint assessment of selected literature; adjustments were subsequently made to clarify the categories.

### Quality assessment

Included articles were divided between the two review teams for quality assessment. Each reviewer assessed the quality of included papers and reconciled ratings within their respective teams using the Mixed Method Appraisal Tool (MMAT) [28]. The MMAT was designed for the appraisal stage of systematic mixed studies reviews that combines qualitative, quantitative, and/or mixed methods studies. Grey literature was assessed for quality using the Authority, Accuracy, Coverage, Objectivity, Date, Significance (AACODS) checklist designed to enable evaluation and critical appraisal of grey literature [29]. Articles were not excluded based on the results of their critical appraisal, as papers of lesser quality may still facilitate the characterization of CHW programmes, CHW roles, their impact on malaria and other health outcomes, or describe factors contributing to the sustainability of CHW programmes. Additionally, outcome 2 on extractions for evidence of outcomes or impact of the CHW programme were assessed for the level of evidence, or the degree to which bias was eliminated from the study design. The assessment was based on the National Health and Medical Research Council (NHMRC) guide complemented with the National Institute for Health and Clinical Excellence (NICE) [30].

### Data synthesis

Thematic coding and descriptive analysis were adopted to identify and synthesize the data and findings for all primary outcomes. Inductive and deductive approaches guided the coding process, and emerging themes from the selected articles were added to the framework where needed. Where required, additional information from web searches also supplemented extracted data to support the characterization of the programmes. For outcome 3 on strategies to ensure sustainability and factors for effective implementation, an interpretive approach [31] and framework-based synthesis [32] were used to derive a conceptual framework for sustainability factors through three complementary processes:

1. Following an initial reading of 3 articles related to CHWs [12, 24, 33], factors that could potentially influence implementation and sustainability of CHW programmes were identified and adapted into a pre-designed extraction template;
2. Concepts derived from the literature during the initial extraction were used to further guide data extraction and categorize findings according to emerging themes;
3. A conceptual framework was developed to synthesize findings. Existing frameworks on malaria programme management [34] and Community Health Worker Assessment and Improvement Matrix (CHW AIM)’s CHW functionality matrix [35] were consulted.

The final framework developed from the process of synthesizing emerging themes with pre-identified factors is in **Table 2**. The PRISMA checklist, search strategies, lists of countries, organisations, information extracted from articles, and adapted tables of the MMAT and ACCODS checklist are in **S1 Appendix**.

**Table 2:** Conceptual framework of factors influencing sustainability of CHW programmes.

**Programme design and management**	◾ How the programme is structured, managed, and linked with the wider public health system ◾ How the programme incorporates information and documentation into the national reporting system ◾ How the programme implements monitoring and evaluation processes and incorporates feedback
**Financing or** **funding**	◾ How the programme is financed, through what source(s), and for how long ◾ How the programme funds and manages its operation
**Support system**	◾ How much political commitment there is to the development and maintenance of the programme ◾ How the programme collaborates with other stakeholders or organisations
**Community ownership and engagement**	◾ How the programme engages or mobilises communities to promote awareness and uptake of CHW services ◾ The extent to which communities are involved with the programme implementation and CHW role
**Capacity building and human resource management**	◾ How motivation (e.g. financial and non-financial incentives) and performance (e.g. workload, clarity of role, contextual factors) of CHWs are managed ◾ How training and supervision is provided to VHWs to maintain and develop their capacity

## Results

### Search results and study types

**Fig 1:**
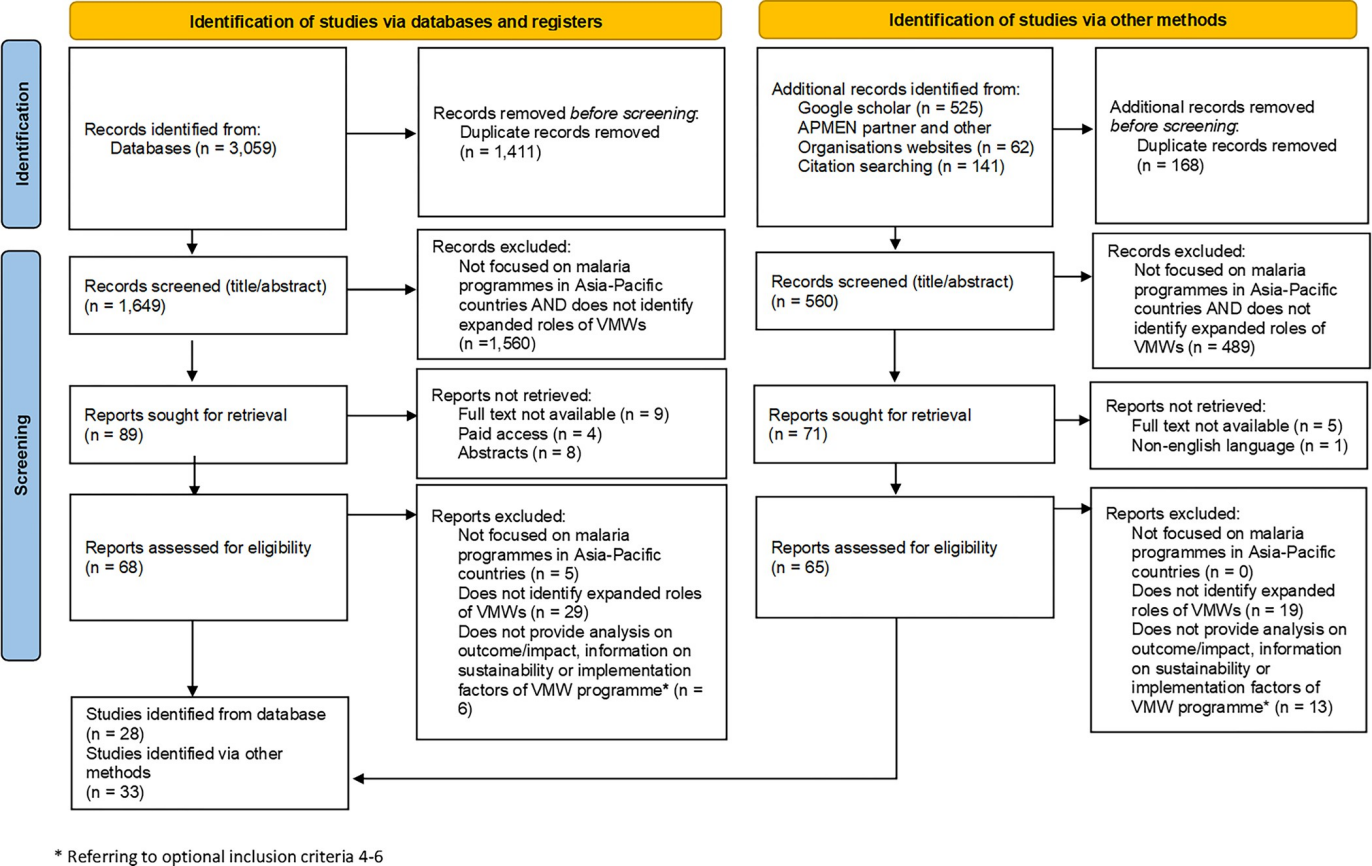
PRISMA 2020 flow diagram of search results from databases, registers and other sources.

3,059 references were identified in the database searches, from which 1,649 unique articles were selected for title and abstract screening (see **S2 Appendix** for the full list of articles). An additional 560 unique records were identified from conducting searches on Google Scholar, implementing organizations website, and citations in articles screened for eligibility. A total of 68 and 65 full-text articles were retrieved and assessed for eligibility from each respective search method. The analysis included 28 studies from database searches and 33 records identified from other methods (see **Fig 1**) comprising: 45 published articles, 11 programme reports, and 5 programme briefs. Of all published articles, 15 were qualitative, 15 quantitative, and 8 mixed methods studies.

All 61 included articles met the first 3 inclusion criteria, of which 33, 33, and 51 articles met the fourth, fifth, and sixth criteria respectively. Among reviewed studies, 43 had a mean MMAT categorical score of 6 out of 7. Based on the ACCODS checklist for reports, 12 programme reports and 2 peer-reviewed articles were on average of fair quality (mean score of 12 out of 14). Programme briefs were also assessed based on the ACCODS checklist for grey literature: 2 programme briefs were of medium quality (scores 17 and 18) and 2 of low quality (scores 8 and 11). The latter 2 briefs lacked details in the significance and/or accuracy dimensions, or otherwise were inapplicable to the checklist due to the nature or format of the literature. Results from the eligibility assessment and quality appraisal are summarized in **S1 Appendix** and provided in full in **S4 Appendix**).

### Characteristics of identified programmes

A total of 31 expanded CHW programmes were identified in 13 countries. The largest number of expanded CHW programs was identified in Myanmar (n = 8), followed by India, Cambodia (n = 5 each), and Indonesia, Lao PDR, and Papua New Guinea (n = 2 each). Only 1 programme was identified in each of the following countries: Afghanistan, Bangladesh, Iran, Nepal, Pakistan, Philippines, and Sri Lanka (**Table 3**). Among included programmes, 19 were still active while 9 were no longer active. These included 6 short pilot programmes [63–66, 79, 80, 91–94], 2 that transitioned into other succeeding programmes [61], and 1 discontinued [52]. The statuses of 4 programmes were not specified [55, 71, 98, 99]. A majority of the programmes (n = 21) operated in malaria endemic regions. The other 10 programmes were described as operating nationwide; 9 among which did not specify whether malaria services were being offered by the whole cadre [36, 37, 49, 50, 52, 56, 57, 73, 96, 97, 99–102].

**Table 3:** Identified programmes, implementing institution, countries, CHW cadre, scale, and status.

Country name (abbrev.)	Programme name / Implementing institution	CHW cadre	Scale (number of CHWs, locations)	Start date, status
Afghanistan (AFG) [36, 37]	National CBHC programme (community basic health care) / Ministry of Public Health in partnership with NGOs (names not mentioned)	Community Health Workers (CHW)	26,560 CHWs in 34 provinces	2003, active [38]
Bangladesh (BNG) [39]	Malaria Control Programme / Partnership between Bangladesh Rural Advancement Committee, the National Malaria Control Programme, and 20 partner NGOs	Shasthya Shebika (SS) Shasthya Komi (SK)	3,132 SS and 686 SK in 13 high malaria endemic districts	2007, active [2]
India (IND-I) [40–44]	Accredited Social Health Activists (ASHA) programme / State-level management under the National Rural Health Mission (NRHM), Ministry of Health and Family Welfare	Accredited Social Health Activists (ASHA)	971,000 ASHAs, 376,017 ASHA trained on malaria services in high endemic areas across 24 states	2005, active
India (IND-II) [45–48]	Mandla Malaria Elimination Demonstration Project (MEDP) / Indian Council of Medical Research, National Institute of Tribal Health, Government of Madhya Pradesh and the Foundation of Disease Elimination and Control (Corporate Social Responsibility initiative under Sun Pharmaceuticals Industry)	Village Malaria Worker (VMW) Accredited Social Health Activists (ASHA) Auxiliary Nurse Midwife (ANW) Multipurpose Health Worker (MPW)	MEDP-specific 235 VMWs and 25 VMW coordinators, and existing system comprising of 1300 ASHA per village), and approximately 1 ANM and 1 MPW per sub-center in 1233 villages in Mandla district	2017, active
India (IND-III) [49, 50]	Mitanin Programme/Department of Health and Family Welfare, Government of Chhattisgarh in collaboration with State Health Resource Center	Mitanins (female health volunteers)	69,000 Mitanins in Chhattisgarh state	2002, active [51]
India (IND-IV) [52]	India’s National Village Health Guides Scheme /Health and Medical Education Committee (Srivastava Committee), Ministry of Health and Family Welfare	Village Health Guide (VHG)	Approximately 500,000 VHGs	1977–2002, discontinued
India (IND-V) [53]	Multipurpose Health Scheme / National Malaria Eradication Programme, National Vector Borne Disease Control Programme	Multipurpose Health Workers (MPW)	52,215 MPW trained in malaria services in high endemic areas across 24 states	1973, active [54]
Indonesia (IDN-I) [55]	Integrated Malaria and Maternal and Child Health Programme (MiP-MCH) / Ministry of Health, UNICEF, Indonesian Society of Obstetrics and Gynaecology	Midwives	34 provinces in 511 districts	2016, not specified
Indonesia (IDN-II) [56]	Posyandu (integrated health care post) Programme / Ministry of Home Affairs, Ministry of Health, Family Welfare Movement (Pemberdayaan Kesejahteraan Keluarga or PKK) and Village Community Health (Pembangungan Kesehatan Masyarakat Desa or PKMD)	Kaders	Over 1 million kaders nationwide	1984, active
Iran (IRN) [57]	Iran’s Community Health Worker Programme Ministry of Health and Education, Tehran University, WHO (at piloting phase)	Behvarz	34,000 Behvarzs (2019)	1984, active
Cambodia (KHM- I) [58–60]	VMW programme on appropriate treatment for malaria and childhood illnesses amongst the most vulnerable populations in Cambodia / National Centre for Parasitology, Entomology and Malaria Control (CNM)	Village Malaria Workers (VMW)	1,602 villages in 17 provinces, approximately 400 villages part of the expanded programme	2001, 2009 (expanded roles), active
Cambodia (KHM-II) [61]	Greater Mekong Subregion Elimination of Malaria through Surveillance (GEMS) /Population Services International sub-project collaboration with National Centre for Parasitology, Entomology and Malaria Control (CNM)	Mobile Malaria Workers (MMW)	273 MMWs (prior to 2020) 0 MMVs (2020 discontinued support to 236 and transitioned 39 to NMCP)	2016–2019, transitioned to GEMS+ project in 2022 [62]
Cambodia (KHM-III) [63]	The Roll Out Radical Cure (RORC) Research Project / Mahidol Oxford Tropical Medicine Research Unit with National Centre for Parasitology, Entomology and Malaria Control (CNM)	Village Malaria Workers (VMWs) Lab technicians	28 VMWs and 5 laboratory technicians in Kravanh and Prognil health centers, Kravanh district, Pursat province enrolled in pilot programme from 2,548 VMWs and 275 Mobile Malaria Workers in the national programme	May 2021 –July 2022 (15 months) pilot study
Cambodia KHM-IV [64]	Program to develop novel multiplex point-of-need (PON) diagnostics for surveillance of emerging infectious diseases / U.S. Defense Threat Reduction Agency Joint Science and Technology Office (DTRA-JSTO) with support from the National Malaria Center (CNM) and Naval Medical Research Unit-2 (NAMRU-2)	Village Health Workers (VHW)	45 VHWs in peri urban areas near Phnom Penh were enrolled in pilot study from a larger nation-wide VHW programme	2018, pilot study ended
KHM-V [65, 66]	Sustaining village health worker programmes with expanded roles in the GMS / Action for Health Development Cambodia (AHEAD), Cambodian National Center for Entomology, Parasitology and Malaria Control (CNM), and Mahidol Oxford Tropical Medicine Research Unit (MORU)	Village Malaria Workers (VMWs)	120 VMWs trained on health education package and 9 health centres in 4 districts in Battambang province 105 VMWs from 82 villages in Battambang and Pailin provinces trained to use new diagnostics	18 months during 2021–2023, operational research study ended
Lao PDR (LAO-I) [67–70]	Village Health Volunteer and Village Malaria Workers Programme / National Malaria Control Programme, Lao PDR Center for Malariology, Parasitology, and Entomology (CMPE)	Village Health Volunteers (VHV) Village Malaria Workers (VMWs)	13,722 VHVs and 2,576 VMWs across the country	1980s for VHV programme, 2008 for VMW programme, active
Lao PDR (LAO-II) [61]	Greater Mekong Subregion Elimination of Malaria through Surveillance (GEMS) /Population Services International (PSI) sub-project collaboration with National Malaria Control Programme, Lao PDR Center for Malariology, Parasitology, and Entomology (CMPE)	Shop-based volunteer malaria workers (sVMW) and PSI/Laos supported private providers	9 sVMW and 474 private providers	2016–2019, transitioned to GEMS+ project [62]
Sri Lanka (LKA) [71]	Health Volunteers Programme / Ministry of Health	Health Volunteers (HV)	Approximately 18,000 volunteers (1989)	1976, not specified
Myanmar (MMR-I) [72, 73]	Sun Primary Health (SPH) Network / Population Services International (PSI)	Sun Primary Health (SPH) providers	2,192 SPH/CHWs in 74 townships in Myanmar	2008, active [74]
Myanmar (MMR-II) [75, 76]	MAM Village Health Workers* / Medical Action Myanmar (MAM) and Myanmar National Malaria Control Programme	Village Health Workers (VHW) overall, community health workers (CHWs) in Mon state	2,000 VHWs; among which are 172 CHWs in 4 townships in Mon State	2011, 2015–2016 (expanded roles), active [77, 78]; Programme in Mon state transitioned to Department of Health since 2018
Myanmar (MMR-III) [79, 80]	Myanmar Integrated Community Case Management (iCCM) Pilot Project / Malaria Consortium (MC) in partnership with the Ministry of Health and Sports, the National Malaria Control Programme and township health departments	Malaria Volunteers (MV) superseded by Integrated Community Case Management Volunteers (ICMV)	Sagaing region	2016–2017, the pilot period ended
Myanmar (MMR-IV) [81–85]	Integrated Community Malaria Volunteers Programme*/ Myanmar National Malaria Control Programme and other implementing partners (including Save the Children, and Myanmar Council of Churches)	Integrated Community Malaria Volunteers (ICMVs) preceded by Malaria Volunteers (MV) (2004–2017) or Village Health Volunteers (VHV)	9,074 ICMVs in 218 townships in Myanmar (approximately 38% of originally trained 40,000 MVs still active); 1,500 ICMVs in 47 townships trained to do Malaria Case-Based Reporting (MCBR) since 2018	ICMV training provided from 2016 but malaria volunteer programmes started prior, active
Myanmar (MMR-V) [86]	Integrated Community Malaria Worker (ICMW) / Malaria Elimination Task Force (METF) under the Shoklo Malaria Research Unit (SMRU)	Integrated Community Malaria Worker (ICMW)	ICMWs located at 435 malaria posts—237 located in Hlaingbwe, 152 in Kawkareik, and 46 in Myawaddy	ICMW training rolled out in 2019 but programme started in 2014, active
Myanmar (MMR-VI) [87]	Better Health Together Project / Community Partners International and 6 ethnic health organizations	Integrated Community Malaria Volunteers (ICMVs)	632 volunteers in 17 townships of Kachin, Kayah, Kayin, and Mon State, and Tanintharyi Region	2017, active
Myanmar (MMR-VII) [88–90]	Medical Care Programme / Backpack Health Worker Team (BPHWT)	Backpack Health Worker (BPHW)	1,460 BPHWT members (447 BPHWs, 777 Trained Birth Attendants, and 236 Village Health Volunteers/Workers)	1998, active
Myanmar (MMR-VIII) [91–93]	Mobile Obstetric Maternal Health Worker (MOM) Project / Mae Tao Clinic, Karen Department of Health and Welfare, Burma Medical Association, local ethnic health departments (from Shan, Mon, Karenni, and Karen states), Johns Hopkins Center for Public Health and Human Rights, Global Health Access Program	Community-based maternal health workers, Maternal Health Workers (MHW), Traditional Birth Attendants (TBAs)	33 lay or community-based maternal health workers, 131 MHWs, 288 TBAs in a total of 12 communities in 4 states (Karen, Shan, Karenni, and Mon) with approximate target population of 60,000	2005–2008, 3-year pilot project ended
Nepal (NPL) [94, 95]	Rapid response team (Combined Fever Camp Approach) and Female Community Health Volunteer Programme (Incentive approach) / Visceral Leishmaniasis National Programme and District Public Health Office	Female community health volunteers (FCHVs) as part of the rapid response team	49 local health workers and 76 FCHVs enrolled in Combined fever camp pilot study; the FCHV programme comprises of over 50,000 volunteers	June–August 2016, pilot period ended; programme was stablished since 1988, active
Pakistan (PAK) [96, 97]	National Programme for Family Planning and Primary Health Care or Lady Health Worker Programme (LHW-P) / Federal Development Programme or the Programme for Family Planning and Primary Health Care, Government of Pakistan	Lady health workers (LHWs)	Approximately 100,000 LHWs nationwide	1994, active
Papua New Guinea (PNG-I) [98]	Home-based Management of Malaria / Population Services International (PSI) in collaboration with the PNG National Malaria Control Programme	Volunteer Community-based Distributors (CBDs)	1,000 CBDs trained by PSI	2017, not specified
Papua New Guinea (PNG-II) [99]	Village Health Volunteers / National Department of Health, Papua New Guinea	Marasin meri or Marasin man (Village Health Volunteer or VHV)	Not specified	Not specified
Philippines (PHL) [100–102]	Barangay Health Workers Program / Department of Health and decentralized management by local government units at cities (urban) and municipality (rural) levels, in collaboration with the national malaria control programme and other implementing partners for site specific projects (Agusan del Sur Malaria Control and Prevention Project–ADS-MCP)	Barangay Health Workers (BHWs) Barangay Nutrition Scholars (BNSs)	196,562 BHWs (2009) nationwide	1979 and officially established in 1995 under the Barangay Health Worker Act [103], active

### CHW roles and services

**Fig 2:**
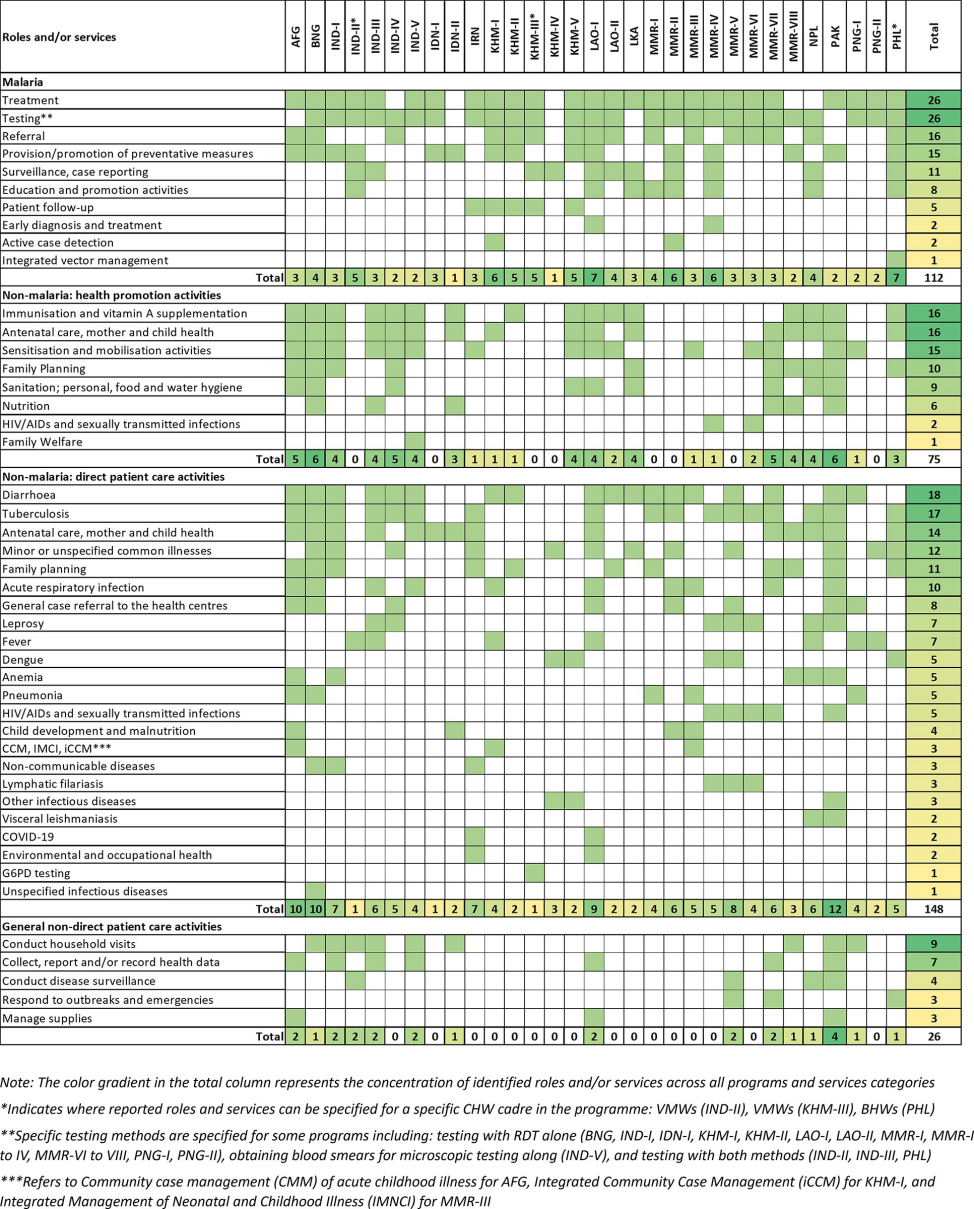
Malaria and non-malarial roles and/or services performed by CHWs.

**Fig 2** reports, at the programme level, roles and/or services performed by CHWs: malaria, non-malaria health promotion, direct patient care, and general activities.

#### Identification of malaria roles and/or services performed by CHWs

Malaria services provided by CHWs were grouped into 10 categories (**Fig 2**): treatment; testing; referral of severe cases; provision and/or promotion of preventive measures and vector control activities; surveillance and case reporting; education and promotion activities; treatment follow-up with patients; early diagnosis and treatment; active case detection; and, integrated vector management. Description of roles performed under each service category are found in **S2 Appendix**. Malaria treatment and/or testing were most frequently identified in 26 of 31 programmes; both services were provided as a package in 23 programmes, but in some programmes CHWs only provide testing (n = 3) or treatment (n = 3). Across all programmes, CHWs performed an average of 3.6 types of malaria services which ranged from provision of a single type of service (provision of insecticide-treated nets and larvicide by Kaders in Indonesia [56], and performance of malaria surveillance activities by Village Health Workers in peri-urban settings of Cambodia [64] to coverage of 7 services (Village Health Volunteers in Lao PDR [67–70], and Barangay health workers in the Philippines [100–102].

#### Identification of non-malaria roles and/or services performed by CHWs

Non-malaria services identified were grouped into 3 categories of activity: health promotion, direct patient care, and general non-direct patient care. Eight categories of health promotion activities were identified, with the most common being promoting immunisation and vitamin A supplementation, and services relating to antenatal care (ANC) and mother and child health (MCH) (n = 16 each), followed by conducting community sensitisation and mobilisation activities (n = 15). CHWs were also found to provide family planning education (n = 10); promote sanitation (n = 9), provide counselling on nutrition (n = 6), and/or health education on human immunodeficiency virus (HIV) and sexually transmitted infections (STIs) (n = 2) or were assigned responsibilities under a family welfare programme (n = 1).

Direct patient care, which includes screening, testing, treatment, and/or referral services for community members, were performed by CHWs across 21 health or disease categories. Most frequent were services relating to diarrhoea (n = 18), closely followed by tuberculosis (n = 17). CHWs also provided services relating to ANC and MCH (n = 14); minor or unspecified common illnesses (n = 12); family planning (n = 11); and, acute respiratory infections (ARI) (n = 10), among others. Few programmes described CHWs providing services for non-communicable diseases (NCDs), lymphatic filariasis, other unspecified infectious diseases, visceral leishmaniasis, and COVID-19. Only 3 programmes explicitly indicated implementing community case management (CCM) of acute childhood illness, integrated management of childhood illnesses (IMCI), and integrated community case management (iCCM) for children under 5 years. In Cambodia, 3 programmes piloted the use of new diagnostics including quantitative Glucose 6 Phosphate Dehydrogenase (G6PD) biosensors [63], NS1 dengue rapid tests (SD Bioline Dengue Duo) [64], and multiplex biosensors for malaria, dengue virus, zika virus, chikungunya virus, leptospirosis, *Rickettsia typhi*, *Burkholderia pseudomallei*, and *Orientia tsutsugamushi* [66]. Additionally, CHWs performed general non-direct patient care activities such as the performance of household visits for various health programmes (n = 9); collection, reporting and/or recording of health data (n = 7); participation in non-malaria disease surveillance (n = 4); responding to outbreaks of infectious disease (n = 3); and, managing equipment and supplies (n = 3).

### Evidence of outcome and/or impact of the CHW programme

#### Outcome and/or impact on malaria incidence and mortality

**Fig 3:**
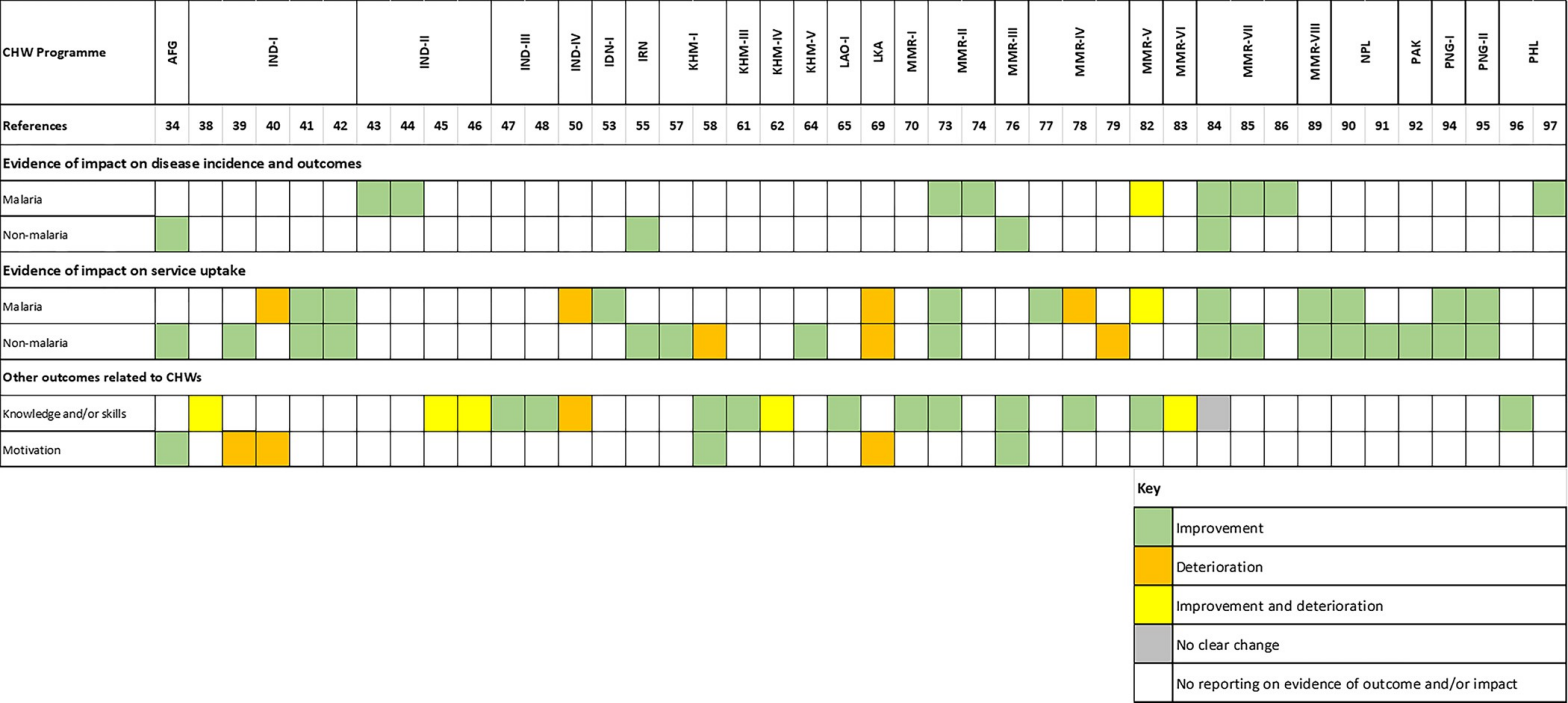
Evidence of outcome and/or impact of the CHW programmes.

Following CHW implementation, decreases in malaria incidence were reported for 4 programmes (**Fig 3**). Medical Action Myanmar (MAM) used a negative binomial mixed effects regression to assess rates of decline in *Plasmodium falciparum* (Pf) and *Plasmodium vivax* (Pv) incidence rates and rapid diagnostic test (RDT) positivity rates with each year of CHW operation (level III-3 evidence based on the NHMRC and NICE level) [75]. A retrospective analysis was also conducted on a subset of CHWs managed by MAM in hard-to-reach communities in Mon state, finding declines in Pf incidence (including mixed infections) and RDT positivity rate by 70% and 69%, respectively, and declines in Pv incidence and RDT positivity rates by 56% and 53%, respectively, over 8 years (level III-3) [76]. The Malaria Elimination Taskforce (METF) also used a negative binomial mixed-effects model to assess rates of decline for the same outcomes as MAM, but they observed mixed findings with the incidence of Pf declining since programme establishment in 2014 not being observed for Pv (level IV) [86]. Other programmes in Myanmar [88–90], as well as in India [45–47] and the Philippines [101] are case reports which include decreases in malaria incidence, and were thus considered level V evidence. There were no reports of impact on mortality or other malaria outcomes. Detailed findings on evidence are available in **S5 Appendix**.

#### Outcome/impact on non-malaria disease incidence and mortality

Improvements in incidence and outcome of non-malaria diseases or health issues (level V evidence) were reported for 4 programmes [36, 57, 80, 88]. For Afghanistan, a decrease in the total fertility rate from 6.7 to 5.3, maternal mortality ratio from 1,600 to 661 per 100,000 live births, and under-five mortality rate from 257 to 55 per 1,000 live births were reported during programme implementation from 2003–2013 [36]. In Iran, Behvarzs working in rural areas were reported to have contributed to reducing the rural-urban gap in health status [57], namely the decrease in infant mortality rate from 123.7 to 30.2 per 1,000 live births in rural areas, and from 60.4 to 27.7 in urban area between 1976 and 2000. By 2017, these rates further declined to 15.8 and 11.3 in rural and urban areas, respectively.

#### Outcome and/or impact on malaria and non-malaria service uptake

Measures of malaria service uptake were reported for 12 programmes: 7 programmes reported increased uptake of malaria services [55, 75, 88, 93, 94, 98, 99], 2 reported decreased uptake [52, 71], and 3 reported mixed findings [81, 82, 86]. Measures of service uptake for other diseases were also reported for 14 programmes, with higher uptake in 11 programmes [36, 41, 43, 44, 55, 57, 75, 88, 89, 93–99], lower uptake in 2 [71, 83], and mixed findings in 1 [59, 60]. In Myanmar, communities preferred to receive services from midwives and/or health facility staff over Integrated Community Malaria Volunteer (ICMV) services negatively impacting uptake [83]. However, the integrated package was shown to sustain increase in blood examination rates in one study [75], while in another, there was no significant impact on RDT testing rates but high rates of consultations within 48 hours of fever onset (an indicator for service uptake is the delay between fever onset and consultation) [86]. Uptake of malaria screening and use of insecticide treated nets (ITNs), among other ANC and MCH services, were also significantly improved among pregnant women in the MOM Project [93]. In Cambodia, a higher percentage of caregivers was found to visit VMWs for child health services in villages where VMW roles were expanded when compared to villages where the service has not been integrated [59], meanwhile, another study found only a minority of caregivers used VMW services for their children in villages where VMWs had expanded their roles [60].

#### Other outcomes related to CHWs

CHWs’ knowledge and/or skills were measured for 16 CHW programmes; 10 programmes found improvements [49, 50, 60, 63, 67, 72, 75, 80, 82, 86, 100], 1 deterioration [52], 4 mixed findings [40, 41, 47, 48, 64, 87], and 1 no clear change [88]. In Myanmar, it was reported that 99% and 94% of malaria volunteers (MVs) in 1 programme were correctly prescribing amoxicillin and cotrimoxazole, respectively; 95% were able to count respiratory rates correctly, 98% could accurately identify severe signs of pneumonia, 90% could correctly identify cases for referral, 91% were able to assess malnutrition using mid upper-arm circumference tape measurement, and 90% could accurately use a weighing scale [80]. RDT quality control assessments found improvements in the recording of RDT results by ICMWs since their roles were expanded [86]. Another programme in Myanmar found that while 94.6% malaria patients received the correct treatment by ICMVs, less than half (44.6%) received both correct and timely treatment (patients received the correct treatment within 24 hours of fever onset). In the same programme, village-based ICMVs were found to be significantly more likely to provide correct treatment than health posts [87]. In Cambodia, 2 feasibility studies found that VMWs were able to progressively improve their skills in using G6PD biosensors and provide primaquine for radical treatment of *Plasmodium vivax* malaria [63], and to perform dengue antigen–antibody RDTs, combined malaria/C-reactive protein (CRP) tests, and multiplexed biosensors [66]. Conversely, another pilot study trialling multiplex RDTs found that competency to use the tests ranged from 26–76% and 23–72%, for the two-line (RDT identified dengue virus and *Burkholderia pseudomallei*) and five-line tests (RDT identified dengue virus, *Plasmodium vivax/falciparum*, *Yersinia pestis*, B. pseudomallei) [64].

In India, Accredited Social Health Activists (ASHAs) provide local and peripheral healthcare services, mainly for MCH, and more recently for non-communicable and communicable diseases programmes; in high-malaria endemic areas, ASHAs were trained to diagnose and treat malaria. In 2011, it was reported that 85% of ASHAs in malaria endemic states had correct knowledge about malaria diagnosis with blood smears, but their knowledge of drug choice for malaria was much lower [40]. Additionally, a 2019 Knowledge, Attitudes, and Practice (KAP) study of ASHAs working under the Mandla Malaria Elimination Demonstration Project (MEDP) programme also found mixed findings regarding their malaria knowledge and/or skill [47]. The baseline study found only 1 out of 220 ASHAs could interpret results of bivalent Pf and Pv malaria RDTs correctly and less than 15% can identify Pf and Pf positive cases correctly; and while almost all ASHAs knew that mosquitoes were the main means of spread of malaria, a majority lacked knowledge about correct antimalarials and had difficulty recognising age-group specific packs. A 2022 follow-up MEDP study noted improvement in ASHA’s ability to accurately interpret RDT results compared to the baseline survey, but ASHAs’ ability to treat a malaria case according to the national drug policy was still lacking in comparison to Auxiliary Nurse Midwives (ANMs) who were health workers that ASHAs reported to at each sub-center [48].

Assessment of CHW motivation was reported for 5 programmes; 3 programmes found improvements [36, 60, 79], and 2 deteriorations [41, 42, 71]. In India, inadequate healthcare delivery support system and certain working modalities were found to reduce ASHA’s motivation and it was suggested that changes in their management are needed to ensure adequate supportive supervision, enhance skills and knowledge, and provide enabling working modalities [41, 42]. Conversely, a 2012 survey found that 66.3% of VMW respondents in Cambodia reported being more enthusiastic about serving as a VMW since the programme scaled-up to include child health services [60].

#### Strategies to ensure sustainability and factors for effective implementation

**Fig 4:**
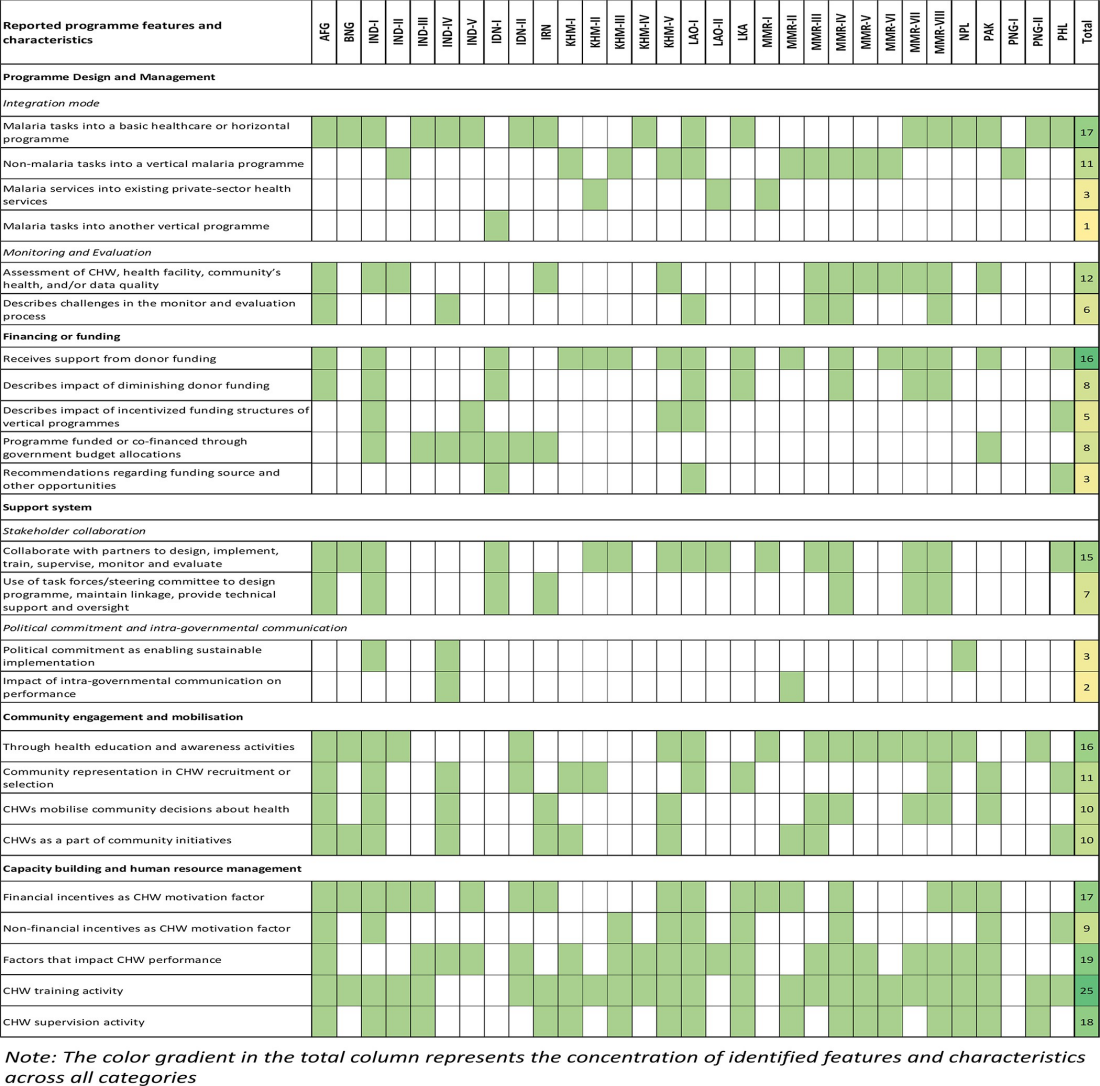
Reported programme features and characteristics based on the conceptual framework of factors influencing sustainability of CHW programmes.

Findings reported in this section are based on a conceptual framework developed to explain factors that are barriers or facilitators to CHW programme implementation (**Fig 4**). Descriptions of findings in this section are based on reported characteristics and features of each programme and do not necessarily imply a causal relationship with sustainability. Additional extracted findings are in **S2 Appendix**.

### Programme design and management

#### Structure and management of CHW programme

Integration of malaria and non-malaria services into a programme that delivers a package of health services was a common design choice in many Asia-Pacific countries. This involved either integrating malaria tasks into another vertical programme (n = 1), integrating non-malaria tasks into a vertical malaria programme (n = 11) or, most commonly, integrating malaria tasks into a basic health care programme (n = 17). Only 3 programmes managed by Population Services International (PSI) in Cambodia, Lao PDR and Myanmar used a private sector expansion model, whereby existing private-sector clinics and pharmacies or private worksites were recruited to provide malaria services [59, 61, 72, 73]. While this strategy expanded services quickly and at low risk by working with local providers that communities are already familiar with [59], the programmes in Cambodia and Lao PDR were phased out in 2020 [62]; mobile malaria workers and shop-based volunteer malaria workers recruited through these programmes had since being integrated into malaria control programmes in each country. Specifically in Lao PDR, the national malaria control program is implementing 2 processes simultaneously whereby VMWs, under the vertical malaria programme, are expanding their roles, while VHVs under the horizontal programme are integrating malaria services into their existing care package [70].

In addition to the integration mode, programmes also identified various channels through which their CHWs were integrated with, and linked to, the local health system. In Indonesia, malaria interventions were included in national guidelines, training materials, and school curricula which facilitated the formalisation of integrating new CHW interventions at multiple levels [55]. ASHAs and village health guides (VHGs) were formalized by their financing through direct national level budget allocation and payment of salaries or incentives through public health centres in India [42, 45, 52]. In terms of recruitment, CHW cadres of pre-existing horizontal programmes were either absorbed into newly integrated programmes [45] or recruited to perform malaria services under a vertical programme in endemic regions [39, 41, 44]. CHWs may be linked to the national health system through their connection with health facilities that provide supervision and training [36, 43, 96], clarification of CHW roles and duties within the wider context of the formal health system [43], and establishment of a clear CHW reporting system that is integrated into the national health management information system [36, 43, 96]. Developing a comprehensive manual was suggested by stakeholders interviewed in Lao PDR to ensure volunteers’ understanding of, and adherence to, duties, policies, procedures, and reference guidelines [69]. When adopting new CHW roles, such as the mobile case-based reporting in Myanmar, lack of standard operating procedures and policies to guide proper operation of new CHW roles were reported challenges to sustainability of these systems [85]. In addition, 3 studies reported piloting or adapting the designs of previous similar programmes to decide which programme design features to maintain, change or prioritize [43, 57, 75].

#### Monitoring and evaluation

Monitoring and evaluation activities were reportedly performed in 10 programmes, with diverse levels of consistency, scope, and target groups. CHW assessments on the workers’ performance, motivation, and constraints were performed in 6 programmes [36, 43, 79, 90, 96]. For 2 programmes, the assessments were systematically conducted every month through meetings with supervisors or coordinators; these assessments informed how functional CHWs were in performing their roles and were consolidated at the district and state levels for the ASHA programme in India [43], or were incorporated into the overall assessment of programme performance for the Lady Health Workers (LHWs) programme in Pakistan [96]. Other programmes assessed CHWs biannually [86] or annually [36, 89]; with 3 programmes implementing ad hoc assessments either before CHWs were trained to identify training needs [47], or after CHWs received training to measure the impact of the training [65, 79, 91].

MEDP in India performed weekly and monthly reviews of surveillance data and drug stock consumption [45–48]. The programme in Afghanistan [36] reported performing routine annual health post functionality assessments, while programmes in Myanmar [83] and Pakistan [96] reported performing impact assessments on care and community health; the programme in Iran [57] reported performing both types of assessments. Comprehensive health records were also used to track individual care and community health indicators to assess and align community health needs with CHW service delivery [57, 96]. Behvarz in Iran also implemented censuses once a year to classify target groups according to the health services [57]. In 2 programmes, intersectoral committees provided technical guidance and evaluated the effectiveness and quality of CHW programmes [57, 81]. External reviews were performed in 2 programmes [40, 90], and in 1 programme an independent review was conducted on an annual basis [46].

Multiple data sources [68], lack of standardized data collection tools [79, 81, 82], and lack of formal feedback mechanisms [36, 52] were described as negatively impacting the monitoring and evaluation process. For example, stakeholders described operational challenges in managing multiple data sources, which impacted routine monitoring and accuracy in maintaining data on CHW demographics, contribution, performance and attrition in Lao PDR [68]. Under the MEDP in India, mHealth tools have been leveraged to digitalize case reporting and supply chain management systems in real-time, which improved data accountability and robustness of the surveillance system and ensured uninterrupted service delivery [45–48]. A steering committee in the MOM project in Myanmar was tasked to conduct periodic field visits to capture and evaluate programmatic activities, however, data gathered were not utilised for evaluation purpose because of their irregularity caused by security constraints, subjectivity in verbal assessments by project workers, and the brevity of the visits [91]. Oversight, therefore, primarily remained through the supervision of daily activities by higher-level staff, along with periodic follow-up training and/or annual follow-up training [91].

### Financing and funding

Over half of the programmes (n = 16) explicitly indicated receiving funding support from donors. Diminishing donor funding was described for 8 programmes as limiting their ability to maintain adequate and ongoing equipment and supplies (n = 5), provide and maintain training and/or supervision (n = 4), perform and feedback evaluations (n = 1), and scale up (n = 1). The negative impacts of incentivized funding structures specific to vertical programmes were described in 5 programmes. As donor funding does not cover non-malarial commodities or activities in Lao PDR, stakeholders have expressed concerns that financial and in-kind support have not increased to match the increasing integrated services [68]. In addition to ongoing policy formation, stakeholders also attributed the challenge to integrating VMWs in Cambodia to the need for funding and resources to support VMWs delivering more complex roles without adequate renumeration [70].

Only 8 programmes reported receiving funding from their government: 2 in Iran [57] and Pakistan [96] reported being largely funded by the central government, 4 in India funded directly through national and state level budget allocations [43, 49, 52, 53], and 2 in Indonesia co-financed at national and district levels [55, 56]. These same programmes also reported budgetary constraints and the inability of national budgets to provide sufficient support [57, 96], or fill the gap as foreign donor funding declines [49, 55].

To sustain funding for integrated programmes, recommendations were made to allocate additional national budgets [70, 101], leverage additional donor funding [55, 70], or identify co-financing opportunities that are in the mutual interests of malaria and non-malaria gains [68]. In Lao PDR, 2 potential scenarios were recommended to support community health care provision by VMWs and VHVs. This included by sustaining donor investment in primary care and new health priorities, and/or drawing on potential domestic funding at the local level, leveraging opportunities from ongoing administrative decentralisation [70]. Conversely, it was found that budget structures of local government units in the Philippines limited the allocation of resources for specific health concerns leading to disrupted health service delivery and/or inadequate coverage of services [88, 89]. Attempts at self-funding programmes were found to be unsuccessful in settings where communities prefer to provide in-kind or no compensation [36], when they perceived CHWs as salaried public health officers [52], or believe that CHW benefit did not outweigh costs [71].

### Support systems

#### Stakeholder collaboration

Across identified programmes, there was collaboration with multiple types of stakeholders at varying levels with 15 programmes explicitly reported collaborating with partners to design (n = 4), implement (n = 11), supervise and train (n = 5), conduct monitoring and evaluation (n = 3), and/or provide technical support (n = 2).

Establishing task forces and steering committees comprising multiple stakeholders ensured that the design of CHW programmes considered the local context, resources, and cultural and health needs of the community [43, 55, 93]. When promoted early, a bottom-up approach that engages multiple stakeholders was reported to contribute to strong national and local ownership of the programme [55], encourage a sense of collective accountability and learning [42, 93], and enhance effective implementation by not duplicating or substituting any pre-existing programme activities [41]. The absence of a formal review process that incorporated input from stakeholders in the selection of disease coverage resulted in a mismatch between the communities’ expectation of health services and those currently being delivered in Myanmar [84] and India [46].

Technical task forces or committees may also be used to maintain effective communication between implementing partners and the national programme, and provide clear policy and technical guidance [36, 40, 81, 90, 91]. Additional oversight was provided by scientific bodies and universities for the Behvarz programme in Iran [57], and an independent monitoring and evaluation committee comprised of technical partners for the MOM programme in Myanmar [92, 93].

#### Political commitment and intra-governmental coordination

In India, 2 programmes described the impact of political commitment on sustainability of CHW programmes. The ASHA programme’s success in Orissa state was attributed to stable leadership and high political commitment at the state level [40]. Conversely, when national commitment declined, the VHG programme, which was designed unilaterally at the national level, was abandoned by many states when the financial burden of managing these programmes fell to them [52]. In Nepal, government investment in building the knowledge, counselling and reporting skills of female community health volunteers (FCHV) and assurance of appropriate supportive supervision links with health workers at the nearest health facility were reported to contribute to programme success [95].

Lack of communication between the central government, where national policies are dictated, and state and district levels, where programmes are implemented, often confused CHW roles and negatively affected their motivation and performance. In India, VHGs were trained in health promotion and preventative activities as dictated by national guidelines, but in practice they were also asked to treat diseases [52]. Lack of coordination between national and township levels in Myanmar resulted in VHWs with different tasks and skillsets providing overlapping services in the same village [75].

### Community engagement and mobilisation

Out of 28 programmes, 19 described performing community engagement and mobilisation activities. Many programmes provided health education by using educational materials (n = 4), organizing discussions about local health concerns (n = 5), making household visits (n = 3), joining or hosting social or cultural events as an opportunity to educate (n = 4), and implementing information education communication (IEC) and behaviour change communication (BCC) activities on malaria preventative measures (n = 1). These activities were reported to promote CHW services and inform community members about, or refer them to, other health providers in the communities in India and Cambodia [41, 59]. In Papua New Guinea, carers were motivated to receive health services from village health volunteers (VHVs) who frequently publicised their activities in and outside of the village [99]. Mobilization activities, such as community dialogues, also helped workers performing integrated roles in Myanmar to earn their communities’ acceptance and trust [72, 79, 80], and were perceived as a crucial step to ensure their support [91]. Incentives to compensate for time and expenses were also suggested to enhance community’s motivation to join health education sessions, especially when these conflicted with household and livelihood responsibilities [65, 66].

Community representation was widely reported (n = 11), including through the selection of CHWs from among the community (n = 7), and involving communities or their representatives in the selection process (n = 4). Gender [36, 70], literacy [40], ethnicity [79, 80], spoken language [91, 92] and residency in the community [36, 42, 71, 91, 92, 96, 101] were important considerations for CHW selection to ensure community acceptance and utilization of services. Participation of the community and transparency in the selection process were perceived to contribute to acceptance and awareness of these workers and their services among hard-to-reach populations [58], internally displaced community [91], and elsewhere [102]. However, community participation could be limited when high-powered individuals designate a candidate without consulting the wider community [52, 71], or when the community bears the costs to replace a CHW with their preferred candidate [52].

CHWs were involved in various community initiatives (n = 10), such as by joining meetings with local government [41] and community committees [36, 37]. CHWs were also reported to mobilise communities about local health needs (n = 9), and by actively organising local meetings, self-help groups, or action groups [36, 37, 42, 57, 101]. In the Philippines, Barangay health workers advocated for malaria control activities through Barangay action committees [96]. The collaboration with local health providers [65, 79, 80] and with community leaders, including monks and teachers [65], increased community’s trust and their acceptance [79, 80], while its absence resulted in CHWs being viewed as competing with other health providers, and thus being less accepted within the community [52, 55, 72]. The establishment of community-owned networks was noted as a mechanism for health workers to receive feedback [75, 83, 84] and build positive relationships with the community [92] in Myanmar, and in other countries to encourage more visits to local health facilities [36, 96].

### Capacity building and human resource management

#### Motivation

Many programmes (n = 17) reported providing CHWs with financial (n = 17) or non-financial (n = 9) incentives. Financial incentives commonly were monthly stipends [40, 41, 43, 72, 91] and per diem allowance or task-based incentives for additional roles [37, 40, 42, 91]. Non-financial incentives included social recognition [36, 41, 71, 81, 102], sense of accomplishment [36, 37] and communal responsibilities [66], knowledge and career advancement [42, 82, 96], as well as being awarded with an academic degree [57] or better career opportunities [71, 82]. In contrast, no payment due to volunteerism [95], delayed payments [57], lack of pension, and payment discrepancy between different cadres of workers [70] demotivated CHWs, while prospects for higher paid work elsewhere was described as increasing CHW attrition rate [44, 70]. ASHAs and MPWs in India, and ICMVs in Myanmar were negatively influenced by performance-based incentives which led them to underperform for uncompensated tasks and be demotivated from actively providing those tasks as malaria incidence declines [40, 46, 53, 56, 67, 82]. Compensation for travel expenses was provided to workers attending training, regular meetings [41, 60, 67] or follow up of patients [63]. Provision of travel allowances and monthly phone bills to ICMVs in Myanmar were additionally suggested to strengthen malaria surveillance activities [85], while trained volunteers also suggested a higher cash incentive to match their increased workload when performing integrated roles [65, 79, 80].

#### Performance

Various factors that affected CHW performance were reported (n = 19). Educational qualifications [96, 99] or written examination [45, 57] have also been used to ensure that selected CHWs can perform their roles well. Such selection processes may be limited by availability of eligible candidates in the local area [97]. When their responsibilities were not clearly defined, CHWs were reported to perform roles that overlapped with other existing health providers [42, 43, 52, 56, 65], resulting in CHWs being less accepted by the community and other health workers [52, 56, 71]. These are common issues in programmes where CHWs were simultaneously recruited by multiple governmental and non-governmental implementers and engaged in different health programmes with various roles, for example, BHWs in the Philippines [102], ASHA in India [41, 42], VHVs in Lao PDR [61, 68], and LHWs in Pakistan [96, 97]. For programmes that managed CHWs with multiple roles, growing workload limited CHW performance [65, 96], resulting in CHWs selectively performing their roles [53] or providing lower quality malaria services [81, 84]. By contrast, CHWs only providing care for a specific health scheme was also described as the reason why clients opted for other health providers for non-malaria conditions [59, 60]. However, high program uptake was also attributed to services being provided free of charge in villages by MAM’s VHWs [76].

CHWs were also limited in their ability to provide continuous services in conflict zones or for internally displaced populations due to security issues and logistical constraints [36, 89, 90, 93]. Effective collaboration across different tiers of CHWs to provide maternal emergency care in situations where facility-based care was not available, as demonstrated by the MOM Project in Myanmar, was described as a successful model for maintaining service continuity and delivery under such conditions [91, 92]. Occasionally, CHWs with low-socioeconomic status may be occupied with paid work elsewhere, limiting their service provision in Cambodia [60] and Lao PDR [61, 70]. In Sri Lanka, the voluntary nature of health volunteers may induce lack of commitment and potentially reduce work ethics [71], although in Nepal, researchers have attributed the low attrition rate of FCHV as evidence of viability of the volunteer model arguing that the voluntary nature helped earn workers the trust and social respect from community members [95].

#### Training

Training was widely provided to CHWs across programmes (n = 15). Many benefits of training were reported, for example, CHW were able to conduct more malaria tests [67, 68], provide better paediatric malaria care [72], collect higher quality data [89, 90], and support their health centers on basic care activities [70]. Conversely, inadequate training reduced CHW abilities to communicate [95, 96], and provide effective care to their patients [58, 60, 72, 94]. Other barriers to training provision included lack of training for the trainers [52], poor access to remote communities [55], and inconsistent provision of supplies and equipment [36] BHWs in the Philippines were reported as being replaced with each change in local administration, and limited budget to provide more frequent training to new workers was reported as having consequences for their functionality [102]. ASHAs working under the MEDP are provided with multiple training opportunities; but due to competing priorities, malaria training was found to be less prioritized than training for maternal health [47].

To improve training for workers, programmes have suggested or implemented the following measures: standardising content across health programmes [36, 76], adapting to local context [67, 91], focusing on providing knowledge and skills in locally prevalent illnesses [86], offering more refreshers [72, 91] and small-group training [104], incorporating on-the-job [81] or practical training sessions [36, 93], and using updated health education [36, 96] and participatory materials [91]. Combining training or workshop sessions between multi-cadres of CHWs or with other primary care staff can also enable CHWs to share their learning experience [89–91] and jointly share recommendations with managerial staff [45]. The MOM project’s Maternal Health Workers (MHWs) were able to offer basic emergency obstetric care at home that was normally considered feasible at facilities, after participating in 6-month training sessions designed by the project team [91, 92]. Additionally, extending the training to CHWs’ assistants [58] or other health workers in the communities [83] may increase service access when CHWs are occupied or absent.

**Supervision.** Details of supervision for CHWs was reported for 18 programmes; with 3 reporting regular supervisory meetings at health centres but inconsistent field activities [52, 68, 79, 80]. Inadequate number and capacity of personnel at the management level [97], especially as many supervisors manage other infectious disease programmes, were reported as challenges [85]. To improve these activities, it was suggested that supervision should be targeted and responsive [46, 82] and conducted continuously [53], while staff performing supervisory roles should monitor necessary CHW supplies [46, 82–84], clearly define the roles and responsibilities for CHWs and programme staff, and work closely with local staff to employ specific interventions such as mass testing and treatment [46], and receive feedback on CHW training [65]. Benefits were also perceived when supervisors could be tasked with monitoring and evaluating CHW’s knowledge and service quality [79, 80], and conducting follow-up information sharing workshops to review fieldwork and logistical arrangements [91] In addition to properly supplying CHWs, it was also suggested that referral centres should be equipped with regular supplies of medicines for common ailments and medical equipment [97].

## Discussion

In the Asia-Pacific, CHWs perform a range of roles and services ranging from direct patient care, health promotion, and general management and reporting activities. Evidence for impact on health outcomes was found for only 8 out of 29 identified expanded CHW programmes with reduction in malaria incidence and improvement in non-malaria indicators being reported. However, there was an absence of higher quality evidence of impact on outcomes from malaria programmes in the region, whereas service uptake, knowledge, skills and motivation of workers were reported with mixed positive and negative results. While many programmes reported broad overlapping features highlighting enabling characteristics, the design of these programmes was highly heterogeneous due to diverse country contexts.

### Prospects for expansion of roles beyond malaria

Our findings reveal that CHWs in the Asia Pacific perform a myriad of both malaria and non-malaria roles like those of CHWs in highly endemic African countries [20]. Generally, the groupings of CHW roles align with the diversity of CHW functions described elsewhere [3, 24, 105]. Beyond malaria services, CHWs across the region were tasked to address a wide range of common illnesses, with the most frequently identified diseases being diarrhoea and tuberculosis, often in combination with community sensitization and mobilization activities. For the GMS countries, this suggests prospects for establishing VMWs as a primary health educator providing knowledge beyond malaria, promoting health and preventative practices within their respective communities. This expanded preventative role may be coupled with delivering services targeting common illnesses, for example, fever screening using multiplex RDTs and health education packages piloted in Cambodia [65, 66], or combinations of health issues for vulnerable groups such as mothers and children under 5, as shown with the successful delivery of integrated services for pregnant women and mothers in Myanmar [91, 92]. Similar to in Africa, iCCM interventions have also been piloted in the Asia Pacific. While only programmes in Afghanistan [36, 37], India [49, 50], and Myanmar [49, 79, 80] explicitly describe implementing iCCM interventions, many programmes provide a similar combination of services including for diarrhoea, tuberculosis, ARI, and ANC/MCH activities.

GMS countries looking to expand VMW roles may also learn from pilot projects in the sub-region where there has been evidence of sustained blood examination rates for malaria post role-expansion [75], or evidence where these workers can be effectively trained to use multiplex RDTs [66], provide G6PD testing [63] or child health services [59, 80]. While our review did not identify evidence for impact of long-running basic health care programmes [39, 40, 57, 96], the GMS countries may learn from how these programmes deliver and sustain a wide range of CHW activities from minor illnesses to NCDs. Qualitative research into health and community stakeholders’ perspective on what roles VMWs can perform can also help identify context specific preferences for services. In Myanmar, implementing partner stakeholders argue that diseases covered in an integrated community-delivered model in the elimination phase should depend on the geographical location of the service [83], while stakeholders in Lao PDR identified the integration of sanitation awareness raising and services for priority diseases such as dengue, diarrhoea, influenza, skin infection and tuberculosis into the current VHV model as the desired community-delivered model for malaria elimination [69, 70].

However, reports of expected roles do not equate actual nor better performance of these roles. In India, “multipurpose” health workers were heavily biased towards vector control activities for which they receive financial incentives [54], while excessive workloads and poor incentives lead ASHAs to neglect low incentive-based roles and other health promotion activities [54, 106, 107]. The increased workload from covering larger populations and performance of multiple tasks has also been found to negatively impact CHW performance in Africa [14]. Beyond the amount of work, role selection should also match CHWs’ capacity and qualifications. In Myanmar, the ICMV programme reported operational challenges with implementing mobile-based case reporting due to the poor information technology literacy of the ICMVs [85]. Conversely, if adequate training, supervision and health system support were available, the addition of tasks was found to not reduce the quality of community case management of malaria [13]. As integration of CHW roles may result in CHWs being overworked or choosing to prioritize roles that provide financial incentives, programmes should be cautious about competing tasks and the financial incentives that may cause performance bias, particularly those funded by donors as malaria declines.

### Gaps in measuring malaria outcomes

Existing literature from other regions has largely investigated the impact of integrated CHW programmes on malaria outcomes relating to high-risk sub-groups such as pregnant women and children under 5. While many found positive impact on mortality, they tended to be CHW programmes in high transmission settings in Africa [17, 21, 108]. With most existing evidence focusing on high-risk sub-groups in African settings, generalising these findings to low transmission and elimination settings in Asia is difficult. In our review, 4 programmes reported positive impacts of integrated CHW programmes on malaria incidence, but none reported impacts on malaria mortality, or age- or pregnancy-specific malaria outcomes. Establishing the impact on mortality or pregnancy outcomes in Asia-Pacific countries could be underwhelming due to the lower numbers of deaths and infected pregnant women in the region than in Sub-Saharan Africa.

This review identified multiple studies evaluating the impact on service uptake for malaria and other diseases, and other CHW functionality indicators, echoing a similar observation made in previous systematic reviews. Furthermore, while prevailing research tends to focus on identifying and evaluating the factors impacting functionality of CHWs such as their knowledge and skills [13, 15], there are still other core CHW roles such as being community activators or bridges which are both critical but more difficult to measure than disease specific outcomes. As the GMS countries move towards elimination and leverage the expansion of VMW roles as a strategy to maintain malaria services, it is crucial for programmes to conduct comprehensive impact evaluations of new interventions. This could be performed by using confirmed malaria incidence as the impact indicator alongside process evaluations [109]. These evaluations may be further complemented with indicators that measure the full spectrum of CHW roles beyond malaria.

### Defining programme features for effective implementation (and sustainability)

Similar to our review, several programme design factors and characteristics observed to be enablers or barriers for CHW programme implementation have been identified in previous reviews [108, 110–114], WHO CHW programme evidence guides [22, 115], and a report by the Community Health Impact Coalition [116]. However, the broad categories and frameworks diverge, and the majority have focused on scaling up and sustaining CHW functions without a particular emphasis on malaria service delivery.

Overall, the effective integration of CHW programs into health systems has previously been found to support programme sustainability and credibility, clarify CHW roles, and foster collaboration between CHWs and higher-level health system actors [15, 24, 110]. In all countries, the design of CHW programmes with integration of malaria roles depends heavily on the pre-existing CHW system in place. In countries where health services are already being offered by an existing CHW, malaria services were added to their service package. In others, where health systems did not already have a pre-existing CHW cadre, donor-initiated malaria CHW programmes were leveraged to expand the coverage of basic health services within remote communities. Expanded CHW programmes should explore ways to integrate or link to the national health system by building on collaboration with key multilateral stakeholders. In Cambodia, it was suggested that integration of VMWs in the formal CHW system will require collaboration with various departments within the Ministry of Health to formulate policy, manage resources, and ensure that VMWs are properly trained, supported, and supervised as they take on tasks beyond malaria [66]. In other settings where CHW programmes are implemented by multiple partners, effective health care model reform will require coordinated and synchronized efforts from all implementing partners including allocating responsibilities to train, supervise, and manage supply chains, among others [85]. Successful examples of effective multi-tier stakeholder collaboration that help maintain service continuity in fragile settings were identified in this review, including the Backpack Health Workers [88–90], MAM’s programme [75, 76], and the MOM [91–93] programme in Myanmar.

Equally important to sustaining expanded programmes are the presence of ongoing funding and political support. Most programmes identified in our review relied on donor funding to scale service coverage, maintain logistical support, provide training and supervision, and execute monitoring and evaluation. Political commitment to these programmes was vital to support allocation of domestic funding in the face of declining donor funding. This review captures some potential ways forward. Decentralization of administrative units in Lao PDR [70] and Thailand [117] presents an opportunity to leverage local funding to support integrating malaria services into community-based care, although this may face constraints of competing local health and non-health priorities. This challenge is not unique to the Asia Pacific. There is growing evidence that, when well-integrated, community-based service provision by CHWs serves as a cost-effective model for extending health services to hard-to-reach communities [118–120]. Unfortunately, 60% of CHW programmes in sub-Saharan Africa were funded by donors, mostly for vertical disease-specific programmes [121]. These CHW programmes therefore face challenges with scaling due to inadequate national funding which is also restricted by competing national health priorities or lack of political support [121]. As donors move towards ensuring the sustainability of malaria investments and contribution to overall health systems strengthening, there is also an opportunity to sustain donor investment in primary care and new health priorities which may integrate malaria services into the basic care package.

### Tailoring and combining motivation package for CHWs with multiple tasks

This review found a range of CHW motivation and performance factors contributing to effective implementation. Similarly, a mix of financial and non-financial incentives, frequent supervision and continuous training, embedding of CHWs into community and health systems, clear definitions of CHW roles, and effective multi-level communication were found to enhance CHW performance in low- and middle-income settings [10, 23, 110]. Apart from programmatic factors, a 2015 systematic review found that contextual factors relating to the community, economy, environment, and health system policy and practice also influenced CHW performance [111]. Stakeholders also highlighted the importance of managing CHW expectations and workload to motivate and retain CHWs in iCCM programmes [112]. A programme in the Philippines found that, unlike older CHWs who may be engaged in these programmes years before renumeration was given to volunteers, younger CHWs demand allowances to engage in training [89]. To avoid overloading VMWs and ensuring their performance, programmes in the GMS should consider contextual factors and ensure strong support systems are in place, as well as tailor incentive packages to fit VMW expectations when seeking to expand services.

### Community engagement approach to empower CHWs and their communities

Community participation in CHW programmes was extensively reported in our review. Past studies have investigated how engaging communities improves uptake of malaria services and interventions, from providing health education [122–124] to strengthening the local communities and health system [24, 110]. Specifically for malaria, community engagement activities have been conducted to maximise the uptake of malaria interventions in at-risk communities, such as mass drug administration in Lao PDR [125], targeted malaria treatment in Myanmar [126], and community-driven vector control measures in the Philippines [101]. Moving towards elimination, programme implementers are suggested to work with communities to take ownership of elimination activities, particularly when programmes aim to detect and treat asymptomatic cases and reach vulnerable populations [127]. Educating community leaders, members and volunteers about the concept of malaria elimination and the importance of testing and surveillance activities are suggestions for increasing support and recognition of CHW malaria and expanded services [65, 66, 85]. Encouraging communities to take ownership of their health needs and services also helps sustain the uptake of new and old CHW services in the long term.

Beyond malaria, the central role for CHWs found in our study was that of a health promoter; however, their potential as a key link between the communities and the health system was also demonstrated. To accommodate this, programmes should consider minimising the barriers hindering CHW performance within the communities. Limited socioeconomic and educational background [60], and gendered notions of care leading to gender imbalance in the selection and creation of primarily female CHWs in various large-scale volunteer CHW programmes [40, 96, 102] may contribute to CHWs being undervalued and underrecognised [128]. For CHWs to be able to maintain their communities’ trust and respect, not only do they have to be empowered, but the health system they are referring their communities to must also be strengthened [129, 130]. Programmes could achieve this by empowering CHWs to take on meaningful and impactful roles that translate into their performance [129], inform CHWs about outcomes of their service delivery [130], and proactively identify and implement adaptive strategies, especially in times of public health emergencies like the recent global pandemic [131]. Previous reviews suggested integrated case management of malaria with other health services at the community level is preferable and sustainable, and expanding roles does not have an impact on the functionality of CHWs [13] if certain conditions are present [2, 13, 110]. Our findings suggest that the method and subsequent success of expanding VMW roles beyond malaria depends heavily on the country context and the respective public health systems in place (Table 4).

**Table 4:** Key discussion points.

**Prospects for expansion of roles beyond malaria**	• **Diverse roles**: In the Asia Pacific, CHWs perform a wide range of roles beyond malaria such as addressing common illnesses like diarrhoea and tuberculosis, and engaging in community sensitization and mobilization activities. •**Expansion opportunities**: In the GMS, malaria CHWs roles may be expanded to include preventative health education and integrated services for various health issues. A pilot project in Cambodia found that CHW’s preventative roles may be coupled with the delivery of services targeting common febrile illnesses. •**Challenges and considerations for expansion**: Effective CHW role expansion will need to address challenges such as workload, performance bias due to financial incentives, and the need for adequate training and supervision to prevent negative impacts on CHW performance.
**Gaps in measuring malaria outcomes **	• **Limited evidence on malaria outcomes**: Existing literature focus on impact of CHW programmes on malaria outcomes among high-risk groups in high transmission settings, limiting generalizability to low transmission and elimination settings in the Asia Pacific. •**More comprehensive evaluations needed**: Many identified studies evaluate impact of CHW programmes on malaria service uptake and CHW functionality, such as CHW knowledge and skills. New interventions will require more comprehensive evaluations using confirmed malaria incidence as an impact indicator and additional indicators that reflect the full range of CHW roles.
**Defining programme features for effective implementation (and sustainability)**	• **Integration and collaboration**: Effective integration of malaria CHW programs into health systems supports program sustainability and credibility, clarifies CHW roles, and fosters collaboration between CHWs and higher-level health system actors. However, the design of such programs depends heavily on the pre-existing CHW system and should involve collaboration with key multilateral stakeholders. •**Funding and political support**: Ongoing funding and political support are vital for sustainability of CHW programs. As donor funding declines, leveraging local funding or integrating malaria services into primary care may help maintain and extend these programmes. However, this may face constraints of competing local health and non-health priorities.
**Tailoring and combining motivation package for CHWs with multiple tasks**	• **Programmatic factors:** CHW performance is enhanced by a combination of financial and non-financial incentives, frequent supervision and training, integration into community and health systems, clear role definitions, and effective communication. •**Contextual factors**: Community, economy, environment, and health system policies also significantly influence CHW performance, alongside managing CHW expectations and workload to maintain motivation and retention.
**Community engagement approach to empower CHWs and their communities**	• **Community participation and ownership:** Community participation in CHW programs significantly improves the uptake of malaria services and interventions, as observed in mass drug administration, targeted malaria treatment, and community-driven vector control. Encouraging communities to take ownership of their health needs and services also helps sustain community-based services. •**Key linkage to care**: CHWs serve as health promoters and key links between communities and the health system. Empowering CHWs is crucial for maintaining community trust and respect, which can be achieved by assigning meaningful roles, and informing CHWs about their service outcomes. CHWs have also displayed their adaptability by maintaining their role as key linkage to care during public health emergencies.

### Strengths and limitations

This review focused on the types of roles that CHWs perform alongside malaria services in the Asia Pacific, adding to previous literature [2, 3, 112, 132], by exploring the delivery of integrated health services, including for malaria, by CHWs in programmes identified in the Asia Pacific. This is as compared to the more typically studied African context, where malaria transmission is much higher. Strengths of this review include the systematic search in 6 databases and Google Scholar, supplemented by grey literature searches from the bibliography and web pages of relevant organisations in the Asia Pacific. Having specific inclusion criteria also allowed for a particular focus on programmes with expanded CHW roles beyond malaria in the region. However, this exclusion applied to malaria CHW programs with expanded roles that did not report on the outcomes of interest (as outlined in **Table 1**), did not document non-malaria services, or were not reported in English. Due to the widely heterogeneous contexts and findings, there was difficulty in synthesising and comparing information on some topics such as implementation and sustainability factors. Insufficient evidence on malaria outcomes limited the ability to analyse the extent to which the expanded programmes have positively impacted malaria. Eligible literature was not excluded based on quality which limited robustness of findings suitable for a more comprehensive analysis of programmes. Additionally, the systematic review is a retrospective investigation of ongoing or past programmes, and the literature searches may not capture programmes that have yet to report on or publish about their work. With the exception of two programmes [45, 57], none of the extracted literature described changes in implementation since the COVID-19 pandemic began. Therefore, a landscaping online survey will be conducted to capture additional expanded CHW programmes that have not been described in the current academic or grey literature, identify gaps and provide a more updated description of programmes included in this review.

## Conclusion

In the Asia Pacific, we identified 31 programmes in 13 countries where CHWs perform a wide range of both malaria and non-malaria roles. Our findings suggest a positive impact of CHWs on malaria incidence as well as other disease outcomes, but there was a lack of higher quality evidence. Presence of monitoring and evaluation mechanisms, multi-sectoral stakeholder collaborations, and provision of sufficient training and supervision for CHWs were key to effective implementation. The integration of programmes into broader health services, ongoing political and funding support, and engagement with the local communities can significantly contribute to sustain the expansion of CHW roles. In the GMS, heterogeneity of country contexts will require countries to adapt their malaria CHW programmes to fit within the local health system and respond to the health needs of the community in order to sustain these services. More importantly, the evidence suggests that the sustainability of these programs could be best achieved by transitioning from vertical approaches to a broader, community-centric model of care.

## Supporting information

S1 Appendix Methods supplementary information. (DOCX)

S2 Appendix Table of all studies identified in the literature search. (XLSX)

S3 Appendix Table of all data extracted from the primary research sources. (XLSX)

S4 Appendix Table of completed risk of bias and quality assessments for each study. (XLSX)

S5 Appendix Results supplementary information. (DOCX)

## Abbreviations

ACD: Active case detection
ACF: Active case finding
ACT: Artemisinin-based combination therapy
AIDS: Acquired immunodeficiency syndrome
AMG: ASHA mentoring group
ANC: Antenatal care
ANM: Auxiliary nurse midwife
ARC: ASHA resource centre
ARI: Acute respiratory infection
ASHA: Accredited social health activist
AWW: Anganwadi worker
BHS: Basic health staff
BPHWT: Backpack health worker team
CBD: Volunteer community-based distributors
CBHW: Community-based health workers
CCM: Community case management
CHEPP: Community health education and prevention program
CHV: Female community health volunteer
CHW: Community health worker
CPR: Contraceptive prevalence rate
EHC: Essential health care
FLW: Frontline health workers
G6PD: Glucose 6 Phosphate Dehydrogenase
HIV: Human immunodeficiency virus
HMM: Home-based malaria management
iCCM: Integrated community case management of childhood illness
ICMV: Integrated community malaria volunteer
IDP: Internally displaced person
IRS: Indoor residual spraying
ITN: Insecticide-treated net
IVM: Integrated vector management
LF: Lymphatic filariasis
LHP: Local health professional
LHW: Lady health worker
MCHP: Maternal and child health program
MCP: Medical care programme
MDA: Mass drug administration
MHW: Maternal health worker
mHealth: Mobile health
MOM: Mobile obstetric maternal health worker project
MV: Malaria volunteer
NCD: Non-communicable disease
NMCP: National malaria control program
NRHM: National Rural Health Mission
Pf: Plasmodium falciparum malaria
PHC: Primary health centre
PNC: Postnatal care
Pv: Plasmodium vivax malaria
RDT: Rapid diagnostic test
TB: Tuberculosis
TBA: Traditional birth attendant
VHV: Village health volunteer
WHO: World Health Organization
