# Supplementary material for: Expanded roles of community health workers beyond malaria in the Asia-Pacific: A systematic review
Source: PLOS Glob Public Health. 2024 Oct 16;4(10):e0003113. doi: 10.1371/journal.pgph.0003113 (PMC11482702; doi:10.1371/journal.pgph.0003113)
Supplement: S1 Appendix — (DOCX) [file pgph.0003113.s001.docx]

**S1 Appendix.** Methods Supplementary Information.

**S1.1 PRISMA-P 2020 Checklist**

| **Section and Topic** | **Item #** | **Checklist item** | **Location where item is reported** |
| --- | --- | --- | --- |
| **TITLE** | | |  |
| Title | 1 | Identify the report as a systematic review. | P1 |
| **ABSTRACT** | | |  |
| Abstract | 2 | See the PRISMA 2020 for Abstracts checklist. | P1 |
| **INTRODUCTION** | | |  |
| Rationale | 3 | Describe the rationale for the review in the context of existing knowledge. | P1-2 |
| Objectives | 4 | Provide an explicit statement of the objective(s) or question(s) the review addresses. | P2 |
| **METHODS** | | |  |
| Eligibility criteria | 5 | Specify the inclusion and exclusion criteria for the review and how studies were grouped for the syntheses. | P4 |
| Information sources | 6 | Specify all databases, registers, websites, organisations, reference lists and other sources searched or consulted to identify studies. Specify the date when each source was last searched or consulted. | P4, S1 Appendix |
| Search strategy | 7 | Present the full search strategies for all databases, registers and websites, including any filters and limits used. | S1 Appendix |
| Selection process | 8 | Specify the methods used to decide whether a study met the inclusion criteria of the review, including how many reviewers screened each record and each report retrieved, whether they worked independently, and if applicable, details of automation tools used in the process. | P4 |
| Data collection process | 9 | Specify the methods used to collect data from reports, including how many reviewers collected data from each report, whether they worked independently, any processes for obtaining or confirming data from study investigators, and if applicable, details of automation tools used in the process. | P4 |
| Data items | 10a | List and define all outcomes for which data were sought. Specify whether all results that were compatible with each outcome domain in each study were sought (e.g. for all measures, time points, analyses), and if not, the methods used to decide which results to collect. | Table 1, Table 2 |
|  | 10b | List and define all other variables for which data were sought (e.g. participant and intervention characteristics, funding sources). Describe any assumptions made about any missing or unclear information. | S1 Appendix |
| Study risk of bias assessment | 11 | Specify the methods used to assess risk of bias in the included studies, including details of the tool(s) used, how many reviewers assessed each study and whether they worked independently, and if applicable, details of automation tools used in the process. | P5 |
| Effect measures | 12 | Specify for each outcome the effect measure(s) (e.g. risk ratio, mean difference) used in the synthesis or presentation of results. | n/a |
| Synthesis methods | 13a | Describe the processes used to decide which studies were eligible for each synthesis (e.g. tabulating the study intervention characteristics and comparing against the planned groups for each synthesis (item #5)). | P5 |
|  | 13b | Describe any methods required to prepare the data for presentation or synthesis, such as handling of missing summary statistics, or data conversions. | n/a |
|  | 13c | Describe any methods used to tabulate or visually display results of individual studies and syntheses. | n/a |
|  | 13d | Describe any methods used to synthesize results and provide a rationale for the choice(s). If meta-analysis was performed, describe the model(s), method(s) to identify the presence and extent of statistical heterogeneity, and software package(s) used. | P5 |
|  | 13e | Describe any methods used to explore possible causes of heterogeneity among study results (e.g. subgroup analysis, meta-regression). | n/a |
|  | 13f | Describe any sensitivity analyses conducted to assess robustness of the synthesized results. | n/a |
| Reporting bias assessment | 14 | Describe any methods used to assess risk of bias due to missing results in a synthesis (arising from reporting biases). | n/a |
| Certainty assessment | 15 | Describe any methods used to assess certainty (or confidence) in the body of evidence for an outcome. | n/a |
| **RESULTS** | | |  |
| Study selection | 16a | Describe the results of the search and selection process, from the number of records identified in the search to the number of studies included in the review, ideally using a flow diagram. | Figure 1 |
|  | 16b | Cite studies that might appear to meet the inclusion criteria, but which were excluded, and explain why they were excluded. | Figure 1 |
| Study characteristics | 17 | Cite each included study and present its characteristics. | S1 Appendix, S2 Appendix  = |
| Risk of bias in studies | 18 | Present assessments of risk of bias for each included study. | S1 Appendix |
| Results of individual studies | 19 | For all outcomes, present, for each study: (a) summary statistics for each group (where appropriate) and (b) an effect estimate and its precision (e.g. confidence/credible interval), ideally using structured tables or plots. | Figure 2-4, S2 Appendix |
| Results of syntheses | 20a | For each synthesis, briefly summarise the characteristics and risk of bias among contributing studies. | n/a |
|  | 20b | Present results of all statistical syntheses conducted. If meta-analysis was done, present for each the summary estimate and its precision (e.g. confidence/credible interval) and measures of statistical heterogeneity. If comparing groups, describe the direction of the effect. | n/a |
|  | 20c | Present results of all investigations of possible causes of heterogeneity among study results. | n/a |
|  | 20d | Present results of all sensitivity analyses conducted to assess the robustness of the synthesized results. | n/a |
| Reporting biases | 21 | Present assessments of risk of bias due to missing results (arising from reporting biases) for each synthesis assessed. | n/a |
| Certainty of evidence | 22 | Present assessments of certainty (or confidence) in the body of evidence for each outcome assessed. | n/a |
| **DISCUSSION** | | |  |
| Discussion | 23a | Provide a general interpretation of the results in the context of other evidence. | P22-25 |
|  | 23b | Discuss any limitations of the evidence included in the review. | P25-26 |
|  | 23c | Discuss any limitations of the review processes used. | P25-26 |
|  | 23d | Discuss implications of the results for practice, policy, and future research. | P25-26 |
| **OTHER INFORMATION** | | |  |
| Registration and protocol | 24a | Provide registration information for the review, including register name and registration number, or state that the review was not registered. | P3 |
|  | 24b | Indicate where the review protocol can be accessed, or state that a protocol was not prepared. | P3 |
|  | 24c | Describe and explain any amendments to information provided at registration or in the protocol. | n/a |
| Support | 25 | Describe sources of financial or non-financial support for the review, and the role of the funders or sponsors in the review. | P37 |
| Competing interests | 26 | Declare any competing interests of review authors. | P37 |
| Availability of data, code and other materials | 27 | Report which of the following are publicly available and where they can be found: template data collection forms; data extracted from included studies; data used for all analyses; analytic code; any other materials used in the review. | P37 |

**S1.2 Countries in the Asia-Pacific**

Afghanistan

American Samoa

Armenia

Australia

Azerbaijan

Bangladesh

Bhutan

Brunei

Cambodia

China

Cook Islands

Democratic People's Republic of Korea

Fiji

French Polynesia

Georgia

Guam

Hong Kong

India

Indonesia

Iran

Japan

Kazakhstan

Kiribati

Kyrgyzstan

Laos

Macau

Malaysia

Maldives

Marshall Islands

Micronesia (Federation states of)

Mongolia

Myanmar

Nauru

Nepal

New Caledonia

New Zealand

Niue

Northern Mariana Islands

Pakistan

Palau

Papua New Guinea

Philippines

Republic of Korea

Russia

Samoa

Singapore

Solomon island

Sri Lanka

Tajikistan

Thailand

Timor-Leste

Tonga

Turkey

Turkmenistan

Tuvalu

Uzbekistan

Vanuatu

Viet Nam

**S1.3 Search Terms and Strategy**

**Search Terms and Strategy**

The search strategy was developed for use with Medline, and was translated and modified for use in the other databases [120], using controlled vocabulary (MESH terms) as appropriate. Reference lists of included papers were searched for potentially relevant papers. In addition to the above database, we also conducted web searches for grey literature on organisations working with malaria programmes in the Asia Pacific region, including reports from government agencies and development agencies.

We follow the WHO definition of CHWs: “Community health workers should be members of the communities where they work, should be selected by the communities, should be answerable to the communities for their activities, should be supported by the health system but not necessarily a part of its organization, and have shorter training than professional workers.” [25] This definition embraces a broader group of CHWs to capture a diverse group of CHWs in different countries.

No restriction was placed on language of publication (although English was used in data extraction). The last search was conducted on 26^th^ February 2024 in order to capture the most recent findings working within the time and resources available.

**Databases and search dates**

- OVID MEDLINE (<http://www.ovid.com/product-details.901.html>) on 26/02/2024
- PUBMED (<https://pubmed.ncbi.nlm.nih.gov>) on 22/02/2024
- EMBASE (<https://libraryguides.mayo.edu/c.php?g=280102&p=1868117>) on 26/02/2024
- Global Health (<https://www.ebsco.com/products/research-databases/global-health>) on 26/02/2024Cochrane Central Register of Controlled Trials (CENTRAL) (<https://www.cochranelibrary.com/advanced-search>) on 26/02/2024
- WHO Global Index Medicus database (<https://www.globalindexmedicus.net/>) on 26/02/2024
- Google Scholar (<https://scholar.google.com/>) on 04/03/2024

**Search Terms**

**Database: Medline (Ovid MEDLINE® Epub Ahead of Print, In-Process & Other Non-Indexed Citations, Ovid MEDLINE® Daily and Ovid MEDLINE®) 1946 to present**

Link to search history: <https://ovidsp.ovid.com/ovidweb.cgi?T=JS&NEWS=N&PAGE=main&SHAREDSEARCHID=6LrLKlWItjZsG2eEaLVtkyJg6YAkiSkbpk0KXOvusvWN7rz24nrdX4c9RmWhhh1wl>

Search Strategy:
1  exp Malaria/ (75799)
2  exp Plasmodium/ (53024)
3  (malaria or plasmodium).ti,ab. (110369)
4  1 or 2 or 3 (120349)
5  Community Health Workers/ (6749)
6  exp Volunteers/ (38599)
7  ("community health worker*" or "community health aide*" or "village health worker*" or volunteer* or "village malaria worker*" or "mobile malaria worker*" or "community malaria agent*" or "basic health staff" or "barefoot doctor*" or "lay health worker*" or "lay health advisor*" or "lay health educator*" or "community health agent*" or "lady health worker*" or "voluntary collaborator*" or "health committee member*" or "community health promoter*" or "health assistant worker*" or "health surveillance assistant*" or "community care giver*" or "community caregiver*" or "accredited social health activist*" or ASHA or mitanin or "traditional midwi*" or "family health team*" or "family health program*" or "community health promotion*" or "home based care*" or "home community based care*").ti,ab. (232131)
8  exp Community Health Services/ (334961)
9  ("community health service*" or "community healthcare" or "integrated community case management" or ICCM or "integrated health service*").ti,ab. (3544)
10  exp "Delivery of Health Care, Integrated"/ (14593)
11  5 or 6 or 7 or 8 or 9 or 10 (598425)
12  (Afghanistan or "American Samoa" or Armenia or Australia or Azerbaijan or Bangladesh or Bhutan or Brunei or Cambodia or China or "Cook Islands" or Fiji or "French Polynesia" or Georgia or Guam or "Hong Kong" or India or Indonesia or Iran or Japan or Kazakhstan or Kiribati or Kyrgyzstan or Laos or Macau or Malaysia or Maldives or "Marshall Islands" or Micronesia or Mongolia or Myanmar or Nauru or Nepal or "New Caledonia" or "New Zealand" or Niue or "Northern Mariana Islands" or Pakistan or Palau or "Papua New Guinea" or Philippines or "Republic of Korea" or Russia or Samoa or Singapore or "Solomon islands" or "Sri Lanka" or Tajikistan or Thailand or Timor-Leste or Tonga or Turkey or Turkmenistan or Tuvalu or Uzbekistan or Vanuatu or "Viet Nam" or Vietnam).tw. (1089872)
13  afghanistan/ or iran/ or turkey/ (83254)
14  exp samoa/ or tonga/ (1113)
15  exp Transcaucasia/ (4877)
16  exp Australia/ (174168)
17  bangladesh/ or bhutan/ or exp india/ or nepal/ or pakistan/ or sri lanka/ (174167)
18  brunei/ or cambodia/ or indonesia/ or laos/ or malaysia/ or myanmar/ or philippines/ or singapore/ or thailand/ or timor-leste/ or vietnam/ (109097)
19  china/ or hong kong/ or macau/ or exp japan/ or exp korea/ or mongolia/ (490873)
20  Polynesia/ (2036)
21  exp Melanesia/ (7209)
22  exp Micronesia/ (2175)
23  exp Asia, Central/ (8101)
24  Indian Ocean Islands/ (843)
25  New Zealand/ (44811)
26  exp Russia/ (56626)
27  12 or 13 or 14 or 15 or 16 or 17 or 18 or 19 or 20 or 21 or 22 or 23 or 24 or 25 or 26 (1619055)
28  4 and 11 and 27 (569)

**Database: Embase 1974 to present**

Link to search history: <https://ovidsp.ovid.com/ovidweb.cgi?T=JS&NEWS=N&PAGE=main&SHAREDSEARCHID=7GSgkaPM19juQvJE3N7BbSKBVrrIl4CCHGCpEN2VZydQ09ygIyj6EhiZXQ8LCpUbo>

Search Strategy:
1  exp malaria/ (104065)
2  exp Plasmodium/ (69772)
3  (malaria or plasmodium).ti,ab. (125759)
4  1 or 2 or 3 (148667)
5  health auxiliary/ (9995)
6  volunteer/ (60955)
7  ("community health worker*" or "community health aide*" or "village health worker*" or volunteer* or "village malaria worker*" or "mobile malaria worker*" or "community malaria agent*" or "basic health staff" or "barefoot doctor*" or "lay health worker*" or "lay health advisor*" or "lay health educator*" or "community health agent*" or "lady health worker*" or "voluntary collaborator*" or "health committee member*" or "community health promoter*" or "health assistant worker*" or "health surveillance assistant*" or "community care giver*" or "community caregiver*" or "accredited social health activist*" or ASHA or mitanin or "traditional midwi*" or "family health team*" or "family health program*" or "community health promotion*" or "home based care*" or "home community based care*").ti,ab. (313422)
8  exp community care/ (133411)
9  ("community health service*" or "community healthcare" or "integrated community case management" or ICCM or "integrated health service*").ti,ab. (4571)
10  integrated health care system/ (13861)
11  5 or 6 or 7 or 8 or 9 or 10 (470370)
12  (Afghanistan or "American Samoa" or Armenia or Australia or Azerbaijan or Bangladesh or Bhutan or Brunei or Cambodia or China or "Cook Islands" or Fiji or "French Polynesia" or Georgia or Guam or "Hong Kong" or India or Indonesia or Iran or Japan or Kazakhstan or Kiribati or Kyrgyzstan or Laos or Macau or Malaysia or Maldives or "Marshall Islands" or Micronesia or Mongolia or Myanmar or Nauru or Nepal or "New Caledonia" or "New Zealand" or Niue or "Northern Mariana Islands" or Pakistan or Palau or "Papua New Guinea" or Philippines or "Republic of Korea" or Russia or Samoa or Singapore or "Solomon islands" or "Sri Lanka" or Tajikistan or Thailand or Timor-Leste or Tonga or Turkey or Turkmenistan or Tuvalu or Uzbekistan or Vanuatu or "Viet Nam" or Vietnam).tw. (1472394)
13  exp south asia/ (281761)
14  exp Pacific islands/ (62516)
15  armenia/ or exp azerbaijan/ or exp "georgia (republic)"/ or exp russian federation/ (71840)
16  exp "Australia and New Zealand"/ (268791)
17  exp Southeast Asia/ (162928)
18  exp China/ (341993)
19  iran/ or "turkey (republic)"/ (110244)
20  japan/ or exp korea/ or philippines/ (295045)
21  ussr/ or kazakhstan/ or kyrgyzstan/ or tajikistan/ or turkmenistan/ or exp uzbekistan/ (42495)
22  maldives/ (475)
23  12 or 13 or 14 or 15 or 16 or 17 or 18 or 19 or 20 or 21 or 22 (2042239)
24  4 and 11 and 23 (814)

**Database: Global Health <1973 to 2024 Week 08>**

Link to search history: <https://ovidsp.ovid.com/ovidweb.cgi?T=JS&NEWS=N&PAGE=main&SHAREDSEARCHID=13CUuxzs8cS72M0wmJTZtv0HdbW0TqeFHciBfkVSCG6uh3lTG5gitUrQjtvdFCJiC>

**Search Strategy:**
**1**  exp malaria/ (70489)
**2**  exp Plasmodium/ (91584)
**3**  (malaria or plasmodium).ti,ab. (97390)
**4**  1 or 2 or 3 (100692)
**5**  exp community health workers/ (1978)
**6**  volunteers/ (2656)
**7**  ("community health worker*" or "community health aide*" or "village health worker*" or volunteer* or "village malaria worker*" or "mobile malaria worker*" or "community malaria agent*" or "basic health staff" or "barefoot doctor*" or "lay health worker*" or "lay health advisor*" or "lay health educator*" or "community health agent*" or "lady health worker*" or "voluntary collaborator*" or "health committee member*" or "community health promoter*" or "health assistant worker*" or "health surveillance assistant*" or "community care giver*" or "community caregiver*" or "accredited social health activist*" or ASHA or mitanin or "traditional midwi*" or "family health team*" or "family health program*" or "community health promotion*" or "home based care*" or "home community based care*").ti,ab. (42196)
**8**  community health services/ (5560)
**9**  ("community health service*" or "community healthcare" or "integrated community case management" or ICCM or "integrated health service*").ti,ab. (1433)
**10**  5 or 6 or 7 or 8 or 9 (47947)
**11**  (Afghanistan or "American Samoa" or Armenia or Australia or Azerbaijan or Bangladesh or Bhutan or Brunei or Cambodia or China or "Cook Islands" or Fiji or "French Polynesia" or Georgia or Guam or "Hong Kong" or India or Indonesia or Iran or Japan or Kazakhstan or Kiribati or Kyrgyzstan or Laos or Macau or Malaysia or Maldives or "Marshall Islands" or Micronesia or Mongolia or Myanmar or Nauru or Nepal or "New Caledonia" or "New Zealand" or Niue or "Northern Mariana Islands" or Pakistan or Palau or "Papua New Guinea" or Philippines or "Republic of Korea" or Russia or Samoa or Singapore or "Solomon islands" or "Sri Lanka" or Tajikistan or Thailand or Timor-Leste or Tonga or Turkey or Turkmenistan or Tuvalu or Uzbekistan or Vanuatu or "Viet Nam" or Vietnam).tw. (840856)
**12**  afghanistan/ or kazakhstan/ or kyrgyzstan/ or mongolia/ or tajikistan/ or turkmenistan/ or uzbekistan/ (9377)
**13**  exp polynesia/ (4491)
**14**  ussr/ or armenia/ or azerbaijan/ or kazakhstan/ or kyrgyzstan/ or "republic of georgia"/ or russia/ or tajikistan/ or turkmenistan/ or uzbekistan/ (29490)
**15**  australia/ or new zealand/ (73378)
**16**  exp south asia/ (189483)
**17**  brunei darussalam/ or indonesia/ or malaysia/ or myanmar/ or philippines/ or singapore/ or thailand/ (78214)
**18**  exp indochina/ (15428)
**19**  exp china/ (256457)
**20**  exp melanesia/ (5831)
**21**  iran/ (66849)
**22**  exp japan/ (60880)
**23**  exp micronesia/ (1043)
**24**  maldives/ (287)
**25**  korea democratic people's republic/ or korea republic/ (34888)
**26**  east timor/ (392)
**27**  turkey/ (44688)
**28**  11 or 12 or 13 or 14 or 15 or 16 or 17 or 18 or 19 or 20 or 21 or 22 or 23 or 24 or 25 or 26 or 27 (880708)
**29**  4 and 10 and 28 (518)

**Cochrane Central Register of Controlled Trials**

**Issue 2 of 12, February 2024**

#1 (malaria or plasmodium):ti,ab,kw 7597

#2 (("community health" NEXT worker*) or ("community health" NEXT aide*) or ("village health" NEXT worker*) or volunteer* or ("village malaria" NEXT worker*) or ("mobile malaria" worker*) or ("community malaria" NEXT agent*) or "basic health staff" or (barefoot NEXT doctor*) or ("lay health" NEXT worker*) or ("lay health" NEXT advisor*) or ("lay health" educator*) or ("community health" NEXT agent*) or ("lady health" NEXT worker*) or (voluntary NEXT collaborator*) or ("health committee" NEXT member*) or ("community health" NEXT promoter*) or ("health assistant" NEXT worker*) or ("health surveillance" NEXT assistant*) or ("community care" NEXT giver*) or (community NEXT caregiver*) or ("accredited social health" NEXT activist*) or ASHA or mitanin or (traditional NEXT midwi*) or ("family health" NEXT team*) or ("family health" NEXT program*) or ("community health" NEXT promotion*) or ("home based" NEXT care*) or ("home community based" NEXT care*)):ti,ab,kw 86851

#3 (("community health" service*) or "community healthcare" or "integrated community case management" or ICCM or ("integrated health" NEXT service*)):ti,ab,kw 3450

#4 #2 or #3 89198

#5 (Afghanistan or "American Samoa" or Armenia or Australia or Azerbaijan or Bangladesh or Bhutan or Brunei or Cambodia or China or "Cook Islands" or Fiji or "French Polynesia" or Georgia or Guam or "Hong Kong" or India or Indonesia or Iran or Japan or Kazakhstan or Kiribati or Kyrgyzstan or Laos or Macau or Malaysia or Maldives or "Marshall Islands" or Micronesia or Mongolia or Myanmar or Nauru or Nepal or "New Caledonia" or "New Zealand" or Niue or "Northern Mariana Islands" or Pakistan or Palau or "Papua New Guinea" or Philippines or "Republic of Korea" or Russia or Samoa or Singapore or "Solomon islands" or "Sri Lanka" or Tajikistan or Thailand or Timor-Leste or Tonga or Turkey or Turkmenistan or Tuvalu or Uzbekistan or Vanuatu or "Viet Nam" or Vietnam):ti,ab,kw 107511

#6 #1 and #4 and #5 122

**World Health Organization Global Index Medicus**

https://pesquisa.bvsalud.org/gim/?output=&lang=en&from=&sort=&format=&count=&fb=&page=1&index=tw&q=%28tw%3A%28malaria+or+plasmodium%29%29+AND+%28tw%3A%28%28%22community+health+worker*%22+or+%22community+health+aide*%22+or+%22village+health+worker*%22+or+volunteer*+or+%22village+malaria+worker*%22+or+%22mobile+malaria+worker*%22+or+%22community+malaria+agent*%22+or+%22basic+health+staff%22+or+%22barefoot+doctor*%22+or+%22lay+health+worker*%22+or+%22lay+health+advisor*%22+or+%22lay+health+educator*%22+or+%22community+health+agent*%22+or+%22lady+health+worker*%22+or+%22voluntary+collaborator*%22+or+%22health+committee+member*%22+or+%22community+health+promoter*%22+or+%22health+assistant+worker*%22+or+%22health+surveillance+assistant*%22+or+%22community+care+giver*%22+or+%22community+caregiver*%22+or+%22accredited+social+health+activist*%22+or+ASHA+or+mitanin+or+%22traditional+midwi*%22+or+%22family+health+team*%22+or+%22family+health+program*%22+or+%22community+health+promotion*%22+or+%22home+based+care*%22+or+%22home+community+based+care*%22+or+%22community+health+service*%22+or+%22community+healthcare%22+or+%22integrated+community+case+management%22+or+ICCM+or+%22integrated+health+service*%22%29%29%29

or

(tw:(malaria or plasmodium)) AND (tw:(("community health worker*" or "community health aide*" or "village health worker*" or volunteer* or "village malaria worker*" or "mobile malaria worker*" or "community malaria agent*" or "basic health staff" or "barefoot doctor*" or "lay health worker*" or "lay health advisor*" or "lay health educator*" or "community health agent*" or "lady health worker*" or "voluntary collaborator*" or "health committee member*" or "community health promoter*" or "health assistant worker*" or "health surveillance assistant*" or "community care giver*" or "community caregiver*" or "accredited social health activist*" or ASHA or mitanin or "traditional midwi*" or "family health team*" or "family health program*" or "community health promotion*" or "home based care*" or "home community based care*" or "community health service*" or "community healthcare" or "integrated community case management" or ICCM or "integrated health service*")))

**Google Scholar**

***Screen the first 10 pages or 100 results for each set:***

(malaria|plasmodium)("community health worker*"|"community health aide*"|"village health worker*"|volunteer*|"village malaria worker*")(Afghanistan|Armenia|Australia|Azerbaijan|Bangladesh|Bhutan|Brunei|Cambodia|China|"Cook Islands"|Fiji|"French Polynesia")

(malaria|plasmodium)("community health worker*"|"community health aide*"|"village health worker*"|volunteer*|"village malaria worker*")(Georgia|Guam|"Hong Kong"|India|Indonesia|Iran|Japan|Kazakhstan|Kiribati|Kyrgyzstan|Laos|Macau|Malaysia|Maldives|"Marshall Islands"|Micronesia)

(malaria|plasmodium)("community health worker*"|"community health aide*"|"village health worker*"|volunteer*|"village malaria worker*")(Mongolia|Myanmar|Nauru|Nepal|"New Caledonia"|"New Zealand"|Niue|"Northern Mariana Islands"|Pakistan|Palau)

(malaria|plasmodium)("community health worker*"|"community health aide*"|"village health worker*"|volunteer*|"village malaria worker*")("Papua New Guinea"|Philippines|"Republic of Korea"|Russia|Samoa|Singapore|"Solomon islands"|"Sri Lanka"|Tajikistan)

(malaria|plasmodium)("community health worker*"|"community health aide*"|"village health worker*"|volunteer*|"village malaria worker*")(Thailand|Timor-Leste|Tonga|Turkey|Turkmenistan|Tuvalu|Uzbekistan|Vanuatu|"Viet Nam"|Vietnam)

(malaria|plasmodium)("mobile malaria worker*"|"community malaria agent*"|"basic health staff"|"barefoot doctor*"|"lay health worker*"|"lay health advisor*"|"lay health educator*")(Afghanistan|Armenia|Australia|Azerbaijan|Bangladesh|Bhutan|Brunei|Cambodia)

(malaria|plasmodium)("mobile malaria worker*"|"community malaria agent*"|"basic health staff"|"barefoot doctor*"|"lay health worker*"|"lay health advisor*"|"lay health educator*")(China|"Cook Islands"|Fiji|"French Polynesia"|Georgia|Guam)

(malaria|plasmodium)("mobile malaria worker*"|"community malaria agent*"|"basic health staff"|"barefoot doctor*"|"lay health worker*"|"lay health advisor*"|"lay health educator*")("Hong Kong"|India|Indonesia|Iran|Japan|Kazakhstan|Kiribati)

(malaria|plasmodium)("mobile malaria worker*"|"community malaria agent*"|"basic health staff"|"barefoot doctor*"|"lay health worker*"|"lay health advisor*"|"lay health educator*")(Kyrgyzstan|Laos|Macau|Malaysia|Maldives|"Marshall Islands"|Micronesia)

(malaria|plasmodium)("mobile malaria worker*"|"community malaria agent*"|"basic health staff"|"barefoot doctor*"|"lay health worker*"|"lay health advisor*"|"lay health educator*")(Mongolia|Myanmar|Nauru|Nepal|"New Caledonia"|"New Zealand"|Niue)

(malaria|plasmodium)("mobile malaria worker*"|"community malaria agent*"|"basic health staff"|"barefoot doctor*"|"lay health worker*"|"lay health advisor*"|"lay health educator*")("Northern Mariana Islands"|Pakistan|Palau|"Papua New Guinea"|Philippines)

(malaria|plasmodium)("mobile malaria worker*"|"community malaria agent*"|"basic health staff"|"barefoot doctor*"|"lay health worker*"|"lay health advisor*"|"lay health educator*")(Korea|Russia|Samoa|Singapore|"Solomon islands"|"Sri Lanka"|Tajikistan)

(malaria|plasmodium)("mobile malaria worker*"|"community malaria agent*"|"basic health staff"|"barefoot doctor*"|"lay health worker*"|"lay health advisor*"|"lay health educator*")(Thailand|Timor-Leste|Tonga|Turkey|Turkmenistan|Tuvalu|Uzbekistan|Vanuatu)

(malaria|plasmodium)("mobile malaria worker*"|"community malaria agent*"|"basic health staff"|"barefoot doctor*"|"lay health worker*"|"lay health advisor*"|"lay health educator*")("Viet Nam"|Vietnam)

(malaria|plasmodium)("community health agent*"|"lady health worker*"|"voluntary collaborator*"|"health committee member*"|"community health promoter*")(Afghanistan|Armenia|Australia|Azerbaijan|Bangladesh|Bhutan|Brunei|Cambodia|China|"Cook Islands"|Fiji)

(malaria|plasmodium)("community health agent*"|"lady health worker*"|"voluntary collaborator*"|"health committee member*"|"community health promoter*")("French Polynesia"|Georgia|Guam|"Hong Kong"|India|Indonesia|Iran|Japan|Kazakhstan)

(malaria|plasmodium)("community health agent*"|"lady health worker*"|"voluntary collaborator*"|"health committee member*"|"community health promoter*")(Kiribati|Kyrgyzstan|Laos|Macau|Malaysia|Maldives|"Marshall Islands"|Micronesia|Mongolia|Myanmar|Nauru)

(malaria|plasmodium)("community health agent*"|"lady health worker*"|"voluntary collaborator*"|"health committee member*"|"community health promoter*")(Nepal|"New Caledonia"|"New Zealand"|Niue|"Northern Mariana Islands"|Pakistan|Palau)

(malaria|plasmodium)("community health agent*"|"lady health worker*"|"voluntary collaborator*"|"health committee member*"|"community health promoter*")("Papua New Guinea"|Philippines|Korea|Russia|Samoa|Singapore)

(malaria|plasmodium)("community health agent*"|"lady health worker*"|"voluntary collaborator*"|"health committee member*"|"community health promoter*")("Solomon islands"|"Sri Lanka"|Tajikistan|Thailand|Timor-Leste|Tonga)

(malaria|plasmodium)("community health agent*"|"lady health worker*"|"voluntary collaborator*"|"health committee member*"|"community health promoter*")(Turkey|Turkmenistan|Tuvalu|Uzbekistan|Vanuatu|"Viet Nam"|Vietnam)

(malaria|plasmodium)("health assistant worker*"|"health surveillance assistant*"|"community care giver*"|"community caregiver*"|"accredited social health activist*")(Afghanistan|Armenia|Australia|Azerbaijan|Bangladesh|Bhutan|Brunei|Cambodia|China)

(malaria|plasmodium)("health assistant worker*"|"health surveillance assistant*"|"community care giver*"|"community caregiver*"|"accredited social health activist*")("Cook Islands"|Fiji|"French Polynesia"|Georgia|Guam|"Hong Kong"|India)

(malaria|plasmodium)("health assistant worker*"|"health surveillance assistant*"|"community care giver*"|"community caregiver*"|"accredited social health activist*")(Indonesia|Iran|Japan|Kazakhstan|Kiribati|Kyrgyzstan|Laos|Macau|Malaysia|Maldives)

(malaria|plasmodium)("health assistant worker*"|"health surveillance assistant*"|"community care giver*"|"community caregiver*"|"accredited social health activist*")("Marshall Islands"|Micronesia|Mongolia|Myanmar|Nauru|Nepal|"New Caledonia")

(malaria|plasmodium)("health assistant worker*"|"health surveillance assistant*"|"community care giver*"|"community caregiver*"|"accredited social health activist*")("New Zealand"|Niue|"Northern Mariana Islands"|Pakistan|Palau)

(malaria|plasmodium)("health assistant worker*"|"health surveillance assistant*"|"community care giver*"|"community caregiver*"|"accredited social health activist*")("Papua New Guinea"|Philippines|Korea|Russia|Samoa|Singapore|"Solomon islands")

(malaria|plasmodium)("health assistant worker*"|"health surveillance assistant*"|"community care giver*"|"community caregiver*"|"accredited social health activist*")("Sri Lanka"|Tajikistan|Thailand|Timor-Leste|Tonga|Turkey|Turkmenistan|Tuvalu|Uzbekistan|Vanuatu|"Viet Nam"|Vietnam)

(malaria|plasmodium)("health assistant worker*"|"health surveillance assistant*"|"community care giver*"|"community caregiver*"|"accredited social health activist*")(Vanuatu|"Viet Nam"|Vietnam)

(malaria|plasmodium)(ASHA|mitanin|"traditional midwi*"|"family health team*"|"family health program*"|"community health promotion*"|"home based care*"|"home community based care*")(Afghanistan|Armenia|Australia|Azerbaijan|Bangladesh|Bhutan|Brunei|Cambodia)

(malaria|plasmodium)(ASHA|mitanin|"traditional midwi*"|"family health team*"|"family health program*"|"community health promotion*"|"home based care*"|"home community based care*")(China|"Cook Islands"|Fiji|"French Polynesia"|Georgia|Guam)

(malaria|plasmodium)(ASHA|mitanin|"traditional midwi*"|"family health team*"|"family health program*"|"community health promotion*"|"home based care*"|"home community based care*")("Hong Kong"|India|Indonesia|Iran|Japan|Kazakhstan|Kiribati)

(malaria|plasmodium)(ASHA|mitanin|"traditional midwi*"|"family health team*"|"family health program*"|"community health promotion*"|"home based care*"|"home community based care*")(Kyrgyzstan|Laos|Macau|Malaysia|Maldives|"Marshall Islands"|Micronesia)

(malaria|plasmodium)(ASHA|mitanin|"traditional midwi*"|"family health team*"|"family health program*"|"community health promotion*"|"home based care*"|"home community based care*")(Mongolia|Myanmar|Nauru|Nepal|"New Caledonia"|"New Zealand"|Niue)

(malaria|plasmodium)(ASHA|mitanin|"traditional midwi*"|"family health team*"|"family health program*"|"community health promotion*"|"home based care*"|"home community based care*")("Northern Mariana Islands"|Pakistan|Palau|"Papua New Guinea"|Philippines)

(malaria|plasmodium)(ASHA|mitanin|"traditional midwi*"|"family health team*"|"family health program*"|"community health promotion*"|"home based care*"|"home community based care*")(Korea|Russia|Samoa|Singapore|"Solomon islands"|"Sri Lanka"|Tajikistan)

(malaria|plasmodium)(ASHA|mitanin|"traditional midwi*"|"family health team*"|"family health program*"|"community health promotion*"|"home based care*"|"home community based care*")(Thailand|Timor-Leste|Tonga|Turkey|Turkmenistan|Tuvalu|Uzbekistan|Vanuatu)

(malaria|plasmodium)(ASHA|mitanin|"traditional midwi*"|"family health team*"|"family health program*"|"community health promotion*"|"home based care*"|"home community based care*")("Viet Nam"|Vietnam)

(malaria|plasmodium)("community health service*"|"community healthcare"|"integrated community case management"|ICCM|"integrated health service*")(Afghanistan|Armenia|Australia|Azerbaijan|Bangladesh|Bhutan|Brunei|Cambodia|China|"Cook Islands"|Fiji)

(malaria|plasmodium)("community health service*"|"community healthcare"|"integrated community case management"|ICCM|"integrated health service*")("French Polynesia"|Georgia|Guam|"Hong Kong"|India|Indonesia|Iran)

(malaria|plasmodium)("community health service*"|"community healthcare"|"integrated community case management"|ICCM|"integrated health service*")(Japan|Kazakhstan|Kiribati|Kyrgyzstan|Laos|Macau|Malaysia|Maldives|"Marshall Islands"|Micronesia|Mongolia)

(malaria|plasmodium)("community health service*"|"community healthcare"|"integrated community case management"|ICCM|"integrated health service*")(Myanmar|Nauru|Nepal|"New Caledonia"|"New Zealand"|Niue|"Northern Mariana Islands")

(malaria|plasmodium)("community health service*"|"community healthcare"|"integrated community case management"|ICCM|"integrated health service*")(Pakistan|Palau|"Papua New Guinea"|Philippines|Korea|Russia|Samoa|Singapore)

(malaria|plasmodium)("community health service*"|"community healthcare"|"integrated community case management"|ICCM|"integrated health service*")("Solomon islands"|"Sri Lanka"|Tajikistan|Thailand|Timor-Leste|Tonga|Turkey|Turkmenistan|Tuvalu|Uzbekistan)

(malaria|plasmodium)("community health service*"|"community healthcare"|"integrated community case management"|ICCM|"integrated health service*")(Vanuatu|"Viet Nam"|Vietnam)

**PubMed**

(((malaria[Title/Abstract] OR plasmodium[Title/Abstract]) OR (("Malaria"[Mesh]) OR "Plasmodium"[Mesh])) AND (((("community health worker*"[Title/Abstract] or "community health aide*"[Title/Abstract] or "village health worker*"[Title/Abstract] or volunteer* or "village malaria worker*"[Title/Abstract] or "mobile malaria worker*"[Title/Abstract] or "community malaria agent*"[Title/Abstract] or "basic health staff"[Title/Abstract] or "barefoot doctor*"[Title/Abstract] or "lay health worker*"[Title/Abstract] or "lay health advisor*"[Title/Abstract] or "lay health educator*"[Title/Abstract] or "community health agent*"[Title/Abstract] or "lady health worker*"[Title/Abstract] or "voluntary collaborator*"[Title/Abstract] or "health committee member*"[Title/Abstract] or "community health promoter*"[Title/Abstract] or "health assistant worker*"[Title/Abstract] or "health surveillance assistant*"[Title/Abstract] or "community care giver*"[Title/Abstract] or "community caregiver*"[Title/Abstract] or "accredited social health activist*"[Title/Abstract] or ASHA or mitanin or "traditional midwi*"[Title/Abstract] or "family health team*"[Title/Abstract] or "family health program*"[Title/Abstract] or "community health promotion*"[Title/Abstract] or "home based care*"[Title/Abstract] or "home community based care*"[Title/Abstract])) OR (("community health service*"[Title/Abstract] OR "community healthcare"[Title/Abstract] OR "integrated community case management"[Title/Abstract] OR ICCM[Title/Abstract] OR "integrated health service*")[Title/Abstract])) OR (((("Community Health Workers"[Mesh]) OR "Volunteers"[Mesh]) OR "Community Health Services"[Mesh]) OR "Delivery of Health Care, Integrated"[Mesh]))) AND (Afghanistan or "American Samoa" or Armenia or Australia or Azerbaijan or Bangladesh or Bhutan or Brunei or Cambodia or China or "Cook Islands" or Fiji or "French Polynesia" or Georgia or Guam or "Hong Kong" or India or Indonesia or Iran or Japan or Kazakhstan or Kiribati or Kyrgyzstan or Laos or Macau or Malaysia or Maldives or "Marshall Islands" or Micronesia or Mongolia or Myanmar or Nauru or Nepal or "New Caledonia" or "New Zealand" or Niue or "Northern Mariana Islands" or Pakistan or Palau or "Papua New Guinea" or Philippines or "Republic of Korea" or Russia or Samoa or Singapore or "Solomon islands" or "Sri Lanka" or Tajikistan or Thailand or Timor-Leste or Tonga or Turkey or Turkmenistan or Tuvalu or Uzbekistan or Vanuatu or "Viet Nam" or Vietnam)

**S1.4 List of organisations searched for in grey literature**

1. Aga Khan University
2. American Refugee Committee, Myanmar
3. American Refugee Committee, Thailand
4. Armed Forces Research Institute of Medical Science (AFRIMS), Bangkok
5. Asia Regional, U.S. Agency for International Development
6. Asian Collaborative Training Network for Malaria (ACTMalaria), Manila
7. Australian Defence Force Malaria and Infectious Disease Institute, Brisbane
8. Australian Institute of Tropical Health and Medicine (AITHM), James Cook University, Cairns
9. Bill and Melinda Gates Foundation
10. BRAC, Bangladesh
11. Burnet Institute, Melbourne
12. Catholic Relief Service (CRS), Cambodia
13. Center for Tropical Medicine, Faculty of Medicine, Public Health and Nursing, Universitas Gadjah Mada
14. Centers for Disease Control and Prevention (CDC), Atlanta
15. Central Department of Microbiology, Tribhuvan University
16. Clinton Health Access Initiative (CHAI)
17. Community Partners International (CPI)
18. Department of Entomology, Faculty of Agriculture, Kasetsart University, Bangkok,
19. Department of Foreign Affairs and Trade (DFAT)
20. Eijkman-Oxford Clinical Research Unit (EOCRU), Jakarta
21. Environmental Science APAC, Crop Science Division, Bayer Pte Ltd
22. Foundation for Innovative New Diagnostics , Geneva
23. Health Poverty Action (HPA), multiple countries
24. Institute for Global Health Sciences, University of California, San Francisco
25. Intellectual Ventures/ Global Good
26. International Centre for Diarrhoeal Disease Research (icddr,b), Dhaka
27. International Organization for Migration (IOM)
28. Jiangsu Institute of Parasitic Diseases (JIPD)
29. Karolinska Institute
30. Khmer Women Welfare Association (KWWA)
31. London School of Hygiene & Tropical Medicine (LSHTM)
32. Mahidol Oxford Tropical Medicine Research Unit [48]
33. Mahidol Vivax Research Unit (MVRU), Faculty of Tropical Medicine, Mahidol University
34. Malara Free Mekong
35. Malaria Atlas Project , Oxford
36. Malaria Consortium, London
37. Malaria No More (MNM) India
38. Malaria Research Centre, Universiti Malaysia Sarawak (UNIMAS)
39. Malaria Unit, Pasteur Institute in Cambodia
40. Medical Anthropology Unit, Institute of Tropical Medicine (ITM), Antwerp
41. Medicine for Malaria Venture (MMV)
42. Menzies School of Health Research
43. MESA Alliance
44. Minister of Public Health, Indonesia
45. National Vector Borne Disease Control Program (NVBDCP), India
46. Nossal Institute for Global Health, University of Melbourne, Melbourne
47. Papua New Guinea Institute of Medical Research (PNG IMR)
48. Parasitology Department, Research Institute for Tropical Medicine (RITM)
49. PATH, Seattle
50. Pattanarak Foundation, Thailand
51. Pilipinas Shell Foundation, Inc. (PSFI), Puerto Princesa City
52. Population Education and Development Association (PEDA), Laos
53. Population Services International
54. Population Services Khermer (PSK), Cambodia
55. QIMR Berghofer Medical Research Institute (QIMR Berghofer), Brisbane
56. Raks Thai Foundation, Thailand
57. Research School of Population Health, Australian National University, Canberra
58. Roll Back Malaria Partnership (RBM)
59. Save the Children-Bangladesh
60. Scaling Up Nutrition Civil Society Alliance (SUN CSA​)
61. School of Public Health, Postgraduate Institute of Medical Education and Research, Chandigarh, India
62. Stella Maris Seafarer’s Center
63. Sumitomo Chemical
64. THINKMD,
65. UNICEF Indonesia
66. University Research Co., LLC (URC)
67. Walter and Eliza Hall Institute (WEHI), Melbourne
68. WHO, Global Malaria Program (GMP)
69. WHO, South-East Asia Regional Office (SEARO)
70. WHO, Western Pacific Regional Office (WPRO)
71. World Vision Foundation of Thailand
72. WorldWide Antimalarial Resistance Network (WWARN), University of Washington, Seattle
73. Young Muslim Association of Thailand

**S1.5** **Full list of information extracted from articles**

1. Title of the study/literature
2. Country where the program/intervention was implemented
3. Institution implementing the programme/intervention
4. Objective(s) of the study/programme
5. Type of study/literature
6. Study design/methodology (if applicable)
7. CHW/VHW cadres
8. Malaria services provided by VHW cadre
9. Non-malaria services provided by VHW cadre
10. VHW compensation for malaria services (if any)
11. Source and duration of funding of the programme
12. Implementation scale (number of VHWs)
13. Implementation period
14. Implementation location (national level, or village/region specific)
15. Evidence for impact/success on malaria/other outcomes
16. Evidence for impact/success of expanded role programme (including training, supervision, logistics, monitoring, financial costs of implementation and funding sources)
17. Facilitators of and barriers to effective implementation
18. Community engagement strategies
19. Strategies used/recommended to ensure sustainability (Community engagement and stakeholder collaborations, training and supervision, financing, other programme design features)

**S1.6 Extracted literature, types of document, methodologies, and eligibility assessments by programme**

| **Programme** | **Title** | **First author, year** | **Search channel** | **Types of document** | **QA tool /**  **Type of literature or methodology** | **Eligibility assessment^1^** | | | | | | **QA Score^2^ (meets the criterion)** |
| --- | --- | --- | --- | --- | --- | --- | --- | --- | --- | --- | --- | --- |
|  |  |  |  |  |  | **1** | **2** | **3** | **4** | **5** | **6** |  |
| AFG | The Community-Based Health Care System of Afghanistan | Aitken, 2020 | Google search | Programme report | AACODS / report | 🗸 | 🗸 | 🗸 | 🗸 | 🗸 | 🗸 | 11 |
|  | Community health worker service delivery for maternal and child health: an observational study from Afghanistan | Kelly, 2022 | Google search | Published article | MMAT / Quantitative nonrandomized | 🗸 | 🗸 | 🗸 | 🗸 | 🗸 | 🗸 | 3 |
| BNG | HNPP at a glance - 2013 | BRAC, 2013 | Org website | Programme brief | AACODS / grey literature | 🗸 | 🗸 | 🗸 | 🗸 | - | - | 8 |
| IND-I | ASHA Which way forward...? Evaluation of ASHA Programme | NHSRC, 2011 | Org website | Programme report | AACODS / report | 🗸 | 🗸 | 🗸 | - | 🗸 | - | 12 |
|  | Assessing community health workers' performance motivation: a mixed-methods approach on India's Accredited Social Health Activists (ASHA) programme | Gopalan, 2012 | Citation search | Published article | MMAT /  Mixed methods | 🗸 | 🗸 | 🗸 | - | 🗸 |  | 6 |
|  | Strengthening malaria service delivery through supportive supervision and community mobilization in an endemic Indian setting: an evaluation of nested delivery models | Das, 2014 | Database | Published article | MMAT /  Quantitative randomized | 🗸 | 🗸 | 🗸 | 🗸 | 🗸 | 🗸 | 6 |
|  | India’s Auxiliary Nurse-Midwife, Anganwadi Worker, and Accredited Social Health Activist Programs | Scott, 2020 | Google search | Programme report | AACODS / report | 🗸 | 🗸 | 🗸 | 🗸 | 🗸 | 🗸 | 12 |
|  | ASHAs: The changing face of malaria control | Sonal, n.d. | Org website | Programme report | AACODS / grey literature | 🗸 | 🗸 | 🗸 | 🗸 | 🗸 | - | 17 |
| IND-II | Study design and operational framework for a community-based Malaria Elimination Demonstration Project (MEDP) in 1233 villages of district Mandla, Madhya Pradesh | Rajvanshi, 2020 | Citation search | Published article | MMAT /  Quantitative Descriptive | 🗸 | 🗸 | 🗸 | - | - | 🗸 | 4 |
|  | A model for malaria elimination based on learnings from the Malaria Elimination Demonstration Project, Mandla district, Madhya Pradesh | Rajvanshi, 2022 | Citation search | Published article | MMAT /  Quantitative Descriptive | 🗸 | 🗸 | 🗸 | - | - | 🗸 | 7 |
|  | Assessment of ASHA for knowledge, diagnosis and treatment on malaria in Mandla district of Madhya Pradesh as part of the malaria elimination demonstration project | Rajvanshi, 2022 | Citation search | Published article | MMAT / Mixed Methods | 🗸 | 🗸 | 🗸 | 🗸 | - | 🗸 | 3 |
|  | Assessment of frontline health workers in providing services for malaria elimination in the tribal district of Mandla, Madhya Pradesh | Rajvanshi, 2022 | Google search | Published article | MMAT / Quantitative nonrandomized | 🗸 | 🗸 | 🗸 | 🗸 | - | - | 7 |
| IND-III | Involvement of Mitanins (female health volunteers) in active malaria surveillance, determinants and challenges in tribal populated malaria endemic villages of Chhattisgarh, India | Chourasia, 2017 | Database | Published article | MMAT / Mixed Methods | 🗸 | 🗸 | 🗸 | - | - | 🗸 | 3 |
|  | Knowledge Attitude and Practices of Mitanin's (Community Health Workers) in Chhattisgarh: Malaria Elimination Perspective | Ranjha, 2021 | Database | Published article | MMAT / Quantitative nonrandomized | 🗸 | 🗸 | 🗸 | 🗸 | - | 🗸 | 7 |
| IND-IV | India’s National Village Health Guides Scheme | Strodel & Perry, 2020 | Google search | Programme report | AACODS / report | 🗸 | 🗸 | 🗸 | 🗸 | 🗸 | 🗸 | 12 |
| IND-V | Changing roles of grass-root level health workers in Kerala, India | Nair, 2001 | Database | Published article | MMAT / Mixed Methods | 🗸 | 🗸 | 🗸 | - | 🗸 | 🗸 | 7 |
| IDN-I | Improving maternal and newborn health services in Eastern Indonesia: Findings from an external review | USAID-UNICEF, 2017 | Org website | Programme report | AACODS / report | 🗸 | 🗸 | 🗸 | - | 🗸 | 🗸 | 12 |
| IDN-II | Knowledge, attitudes, and practices of Anopheles mosquito control through insecticide treated nets and community-based health programs to prevent malaria in East Sumba Island, Indonesia | Bandzuh, 2022 | Database | Published article | MMAT / Qualitative | 🗸 | 🗸 | 🗸 | 🗸 | 🗸 | 🗸 | 5 |
| IRN | Iran’s Community Health Worker Program | Rahbar, 2020 | Google search | Programme report | AACODS / report | 🗸 | 🗸 | 🗸 | 🗸 | 🗸 | 🗸 | 11 |
| KHM- I | Scale-up of community-based malaria control can be achieved without degrading community health workers' service quality: the Village Malaria Worker project in Cambodia | Yasuoka, 2012 | Database | Published article | MMAT / Quantitative descriptive | 🗸 | 🗸 | 🗸 | 🗸 | 🗸 | 🗸 | 7 |
|  | Integrating child health services into malaria control services of village malaria workers in remote Cambodia: service utilization and knowledge of malaria management of caregivers | Hasegawa, 2013 | Database | Published article | MMAT / Quantitative descriptive | 🗸 | 🗸 | 🗸 | 🗸 | 🗸 | 🗸 | 7 |
|  | Improving access to health care amongst vulnerable populations: a qualitative study of village malaria workers in Kampot, Cambodia | Liverani, 2017 | Database | Published article | MMAT / Qualitative | 🗸 | 🗸 | 🗸 | 🗸 | 🗸 | 🗸 | 7 |
| KHM- II | In Search of the Last Malaria Cases: Ethnographic Methods for Community and Private-sector Engagement in Malaria Elimination in Vietnam, Laos, and Cambodia | Masunaga, 2021 | Google search | Published article | MMAT / Qualitative | 🗸 | 🗸 | 🗸 | - | - | 🗸 | 6 |
| KHM-III | Glucose 6 Phosphate Dehydrogenase (G6PD) quantitation using biosensors at the point of first contact: a mixed method study in Cambodia | Adhikari, 2022 | Database | Published article | MMAT / Mixed methods | 🗸 | 🗸 | 🗸 | - | - | 🗸 | 5.5 |
| KHM-IV | Potential for community based surveillance of febrile diseases: Feasibility of self-administered rapid diagnostic tests in iquitos, Peru and Phnom Penh, Cambodia | Morrison, 2021 | Database | Published article | MMAT / Quantitative nonrandomized | 🗸 | 🗸 | 🗸 | - | - | 🗸 | 6.5 |
| KHM-V | Expanding the role of village malaria workers in Cambodia: Implementation and evaluation of four health education packages | Betrian, 2023 | Database | Published article | MMAT / Qualitative | 🗸 | 🗸 | 🗸 | - | 🗸 | 🗸 | 7 |
|  | Expanding the roles of community health workers to sustain programmes during malaria elimination: a meeting report on operational research in Southeast Asia | Dysoley, 2024 | Database | Published article | AACODS / report | 🗸 | 🗸 | 🗸 | 🗸 | 🗸 | - | 13 |
| LAO-I | An assessment of early diagnosis and treatment of malaria by village health volunteers in the Lao PDR | Phommanivong, 2010 | Citation search | Published article | MMAT / Quantitative nonrandomized | 🗸 | 🗸 | 🗸 | 🗸 | 🗸 | 🗸 | 6 |
|  | Evaluating vertical malaria community health worker programs as malaria declines: learning from program evaluations in Honduras and Lao PDR | Napier, 2021 | Database | Published article | MMAT / Mixed methods | 🗸 | 🗸 | 🗸 | - | 🗸 | 🗸 | 6 |
|  | Perspectives of health and community stakeholders on community-delivered models of malaria elimination in Lao People's Democratic Republic: A qualitative study | Oo, 2022 | Database | Published article | MMAT / Qualitative | 🗸 | 🗸 | 🗸 | - | 🗸 | 🗸 | 6.5 |
|  | Prospects for the development of community-based care in remote rural areas: a stakeholder analysis in Laos | Liverani, 2024 | Database | Published article | MMAT / Qualitative | 🗸 | 🗸 | 🗸 | 🗸 | 🗸 | 🗸 | 7 |
| LAO-II | In Search of the Last Malaria Cases: Ethnographic Methods for Community and Private-sector Engagement in Malaria Elimination in Vietnam, Laos, and Cambodia | Masunaga, 2021 | Google search | Published article | MMAT / Qualitative | 🗸 | 🗸 | 🗸 | - | - | 🗸 | 6 |
| LKA | Are large-scale volunteer community health worker programmes feasible? The case of Sri Lanka | Walt, 1989 | Google search | Published article | MMAT / Mixed methods | 🗸 | 🗸 | 🗸 | - | 🗸 | 🗸 | 4 |
| MMR-I | Improving the quality of paediatric malaria diagnosis and treatment by rural providers in Myanmar: an evaluation of a training and support intervention | Aung, 2015 | Org website | Published article | MMAT / Qualitative | 🗸 | 🗸 | 🗸 | - | - | 🗸 | 7 |
|  | 7 iCCM Programs Highlight Diverse Approaches to Reduce Top Child Killers | PSI, 2020 | Org website | Programme brief | AACODS / grey literature | 🗸 | 🗸 | 🗸 | - | - | 🗸 | 11 |
| MMR-II | Malaria elimination in remote communities requires integration of malaria control activities into general health care: an observational study and interrupted time series analysis in Myanmar | McLean, 2018 | Database | Published article | MMAT / Quantitative nonrandomized | 🗸 | 🗸 | 🗸 | 🗸 | 🗸 | 🗸 | 7 |
|  | Successful elimination of falciparum malaria following the introduction of community-based health workers in Eastern Myanmar: A retrospective analysis | Zaw, 2023 | Database | Published article | MMAT / Quantitative nonrandomized | 🗸 | 🗸 | 🗸 | 🗸 | 🗸 | - | 6.5 |
| MMR-III | iCCM in Myanmar: Lessons from Sagaing region | MC, 2018 | Org website | Programme brief | AACODS / report | 🗸 | 🗸 | 🗸 | 🗸 | 🗸 | 🗸 | 12 |
|  | Training malaria volunteers to deliver integrated community case management: Lesson Learnt from Rural Myanmar | MC, 2020 | Org website | Programme brief | AACODS / report | 🗸 | 🗸 | 🗸 | 🗸 | 🗸 | 🗸 | 12 |
| MMR-IV | Myanmar’s Community-Based Health Workers | Davis, 2020 | Google search | Programme report | AACODS / report | 🗸 | 🗸 | 🗸 | 🗸 | 🗸 | 🗸 | 10 |
|  | Assessment of Knowledge and Performance of Village Health Volunteers after Expanding Their Responsibilities in Bago Region, Myanmar, 2017 | Than, 2020 | Google search | Published article | MMAT / Qualitative | 🗸 | 🗸 | 🗸 | 🗸 | - | 🗸 | 6 |
|  | Optimizing Myanmar's community-delivered malaria volunteer model: a qualitative study of stakeholders' perspectives | Oo, 2021a | Database | Published article | MMAT / Qualitative | 🗸 | 🗸 | 🗸 | - | 🗸 | 🗸 | 7 |
|  | Community demand for comprehensive primary health care from malaria volunteers in South-East Myanmar: a qualitative study | Oo, 2021b | Database | Published article | MMAT / Qualitative | 🗸 | 🗸 | 🗸 | - | 🗸 | 🗸 | 7 |
|  | Sustainability of a mobile phone application-based data reporting system in Myanmar's malaria elimination program: a qualitative study | Oo, 2021c | Database | Published article | MMAT / Qualitative | 🗸 | 🗸 | 🗸 | - | - | 🗸 | 7 |
| MMR-V | Longitudinal trends in malaria testing rates in the face of elimination in eastern Myanmar: a 7-year observational study | Rae, 2021 | Database | Published article | MMAT / Quantitative nonrandomized | 🗸 | 🗸 | 🗸 | 🗸 | - | 🗸 | 7 |
| MMR-VI | Quality of Malaria Treatment Provided under ‘Better Health Together’ Project in Ethnic Communities of Myanmar: How Are We Performing? | Minn, 2019 | Database | Published article | MMAT / Quantitative nonrandomized | 🗸 | 🗸 | 🗸 | - | - | 🗸 | 5.5 |
| MMR-VII | Multi-level partnerships to promote health services among internally displaced in eastern Burma | Mahn, 2008 | Citation search | Published article | MMAT / Qualitative | 🗸 | 🗸 | 🗸 | 🗸 | 🗸 | 🗸 | 6 |
|  | BPHWT 10 Years Report 1998-2009. Life, Liberty and the Pursuit of Health: A decade of providing primary health care in Burma's displaced and vulnerable communities | BPHWT, 2010 | Org website | Programme report | AACODS / report | 🗸 | 🗸 | 🗸 | 🗸 | 🗸 | 🗸 | 12 |
|  | BPHWT Annual Report 2018 Provision of Primary Healthcare among the Internally Displaced Persons and Vulnerable Populations of Burma | BPHWT, 2019 | Org website | Programme report | AACODS / report | 🗸 | 🗸 | 🗸 | 🗸 | - | 🗸 | 12 |
| MMR-VIII | Impact of Community-Based Maternal Health Workers on Coverage of Essential Maternal Health Interventions among Internally Displaced Communities in Eastern Burma: The MOM Project | Mullany, 2010 | Citation search | Published article | MMAT / Quantitative descriptive | 🗸 | 🗸 | 🗸 | 🗸 | - | - | 6.5 |
|  | Community-based delivery of maternal care in conflict-affected areas of eastern Burma: Perspectives from lay maternal health workers | Teela, 2009 | Citation search | Published article | MMAT / Qualitative | 🗸 | 🗸 | 🗸 | - | - | 🗸 | 7 |
|  | The MOM Project: Delivering Maternal Health Services among Internally Displaced Populations in Eastern Burma | Mullany, 2008 | Citation search | Published article | AACODS / report | 🗸 | 🗸 | 🗸 | - | - | 🗸 | 13 |
| NPL | Integrating Case Detection of Visceral Leishmaniasis and Other Febrile Illness with Vector Control in the Post-Elimination Phase in Nepal | Banjara, 2019 | Database | Published article | MMAT / Quantitative randomized control trial and Quantitative nonrandomized | 🗸 | 🗸 | 🗸 | - | - | 🗸 | 5 |
|  | A review of the maternal iron and folic acid supplementation programme in Nepal: Achievements and challenges | Paudyal, 2022 | Google search | Published article | MMAT / Mixed methods | 🗸 | 🗸 | 🗸 | 🗸 | - | - | 5.5 |
| PAK | Pakistan’s Lady Health Worker Program | Lassi, 2020 | Google search | Programme report | AACODS / report | 🗸 | 🗸 | 🗸 | 🗸 | 🗸 | 🗸 | 11 |
|  | Perceptions of lady health workers and their trainers about their curriculum for implementing the interventions identified for essential package of health services for Pakistan | Sohail, 2021 | Database | Published article | MMAT / Qualitative | 🗸 | 🗸 | 🗸 | - | - | 🗸 | 7 |
| PNG-I | Reaching the unreachable: leveraging lessons learned from malaria service delivery programs to expand integrated community case management in remote areas of Papua New Guinea | Gheen, 2018 | Org website | Programme brief | AACODS / grey literature | 🗸 | 🗸 | 🗸 | - | - | 🗸 | 18 |
| PNG-II | Village health volunteers’ individual social capital and caretakers’ health service utilization for febrile children in Malaria-endemic villages in Papua New Guinea | Inoue, 2017 | Google search | Published article | MMAT / Quantitative descriptive | 🗸 | 🗸 | 🗸 | - | - | 🗸 | 7 |
| PHL | Diagnosis of malaria in a remote area of the Philippines: comparison of techniques and their acceptance by health workers and the community. | Bell, 2001 | Database | Published article | MMAT / Quantitative nonrandomized | 🗸 | 🗸 | 🗸 | 🗸 | - | - | 5.5 |
|  | Operational efficiency and sustainability of vector control of malaria and dengue: descriptive case studies from the Philippines | van de Berg, 2012 | Database | Published article | MMAT / Qualitative | 🗸 | 🗸 | 🗸 | 🗸 | 🗸 | 🗸 | 7 |
|  | Governance of community health worker programs in a decentralized health system: a qualitative study in the Philippines | Dodd, 2021 | Google search | Published article | MMAT / Qualitative | 🗸 | 🗸 | 🗸 | - | - | 🗸 | 7 |

Note: ^1^ Referring to eligibility criteria no. 1-6; all included literature are eligible for no.1-3; 22, 26, 36 of included literature are eligible for no. 4-6 respectively

^2^ Quality assessment scores indicated counts “yes” responses to meeting criteria of respective assessment methodologies out of total criteria scores 7 for MMAT, 32 for ACCORDS grey literature, and 14 for ACCORDS report

**S1.7 Mixed Methods Appraisal Tool (MMAT) 2018**

| **Type of study** | **Methodological quality criteria** | **Responses** | | |
| --- | --- | --- | --- | --- |
|  |  | **Yes** | **No** | **Comments** |
| 1. Qualitative | S1. Are there clear research questions? |  |  |  |
|  | S2. Do the collected data allow to address the research questions? |  |  |  |
|  | 1.1. Is the qualitative approach appropriate to answer the research question? |  |  |  |
|  | 1.2. Are the qualitative data collection methods adequate to address the research question? |  |  |  |
|  | 1.3. Are the findings adequately derived from the data? |  |  |  |
|  | 1.4. Is the interpretation of results sufficiently substantiated by data? |  |  |  |
|  | 1.5. Is there coherence between qualitative data sources, collection, analysis and interpretation? |  |  |  |
| 2. Quantitative randomized controlled trials | S1. Are there clear research questions? |  |  |  |
|  | S2. Do the collected data allow to address the research questions? |  |  |  |
|  | 2.1. Is randomization appropriately performed? |  |  |  |
|  | 2.2. Are the groups comparable at baseline? |  |  |  |
|  | 2.3. Are there complete outcome data? |  |  |  |
|  | 2.4. Are outcome assessors blinded to the intervention provided? |  |  |  |
|  | 2.5 Did the participants adhere to the assigned intervention? |  |  |  |
| 3. Quantitative nonrandomized | S1. Are there clear research questions? |  |  |  |
|  | S2. Do the collected data allow to address the research questions? |  |  |  |
|  | 3.1. Are the participants representative of the target population?  3.2. Are measurements appropriate regarding both the outcome and intervention (or exposure)? |  |  |  |
|  | 3.3. Are there complete outcome data? |  |  |  |
|  | 3.4. Are the confounders accounted for in the design and analysis? |  |  |  |
|  | 3.5. During the study period, is the intervention administered (or exposure occurred) as intended? |  |  |  |
| 4. Quantitative descriptive | S1. Are there clear research questions? |  |  |  |
|  | S2. Do the collected data allow to address the research questions? |  |  |  |
|  | 4.1. Is the sampling strategy relevant to address the research question? |  |  |  |
|  | 4.2. Is the sample representative of the target population? |  |  |  |
|  | 4.3. Are the measurements appropriate? |  |  |  |
|  | 4.4. Is the risk of nonresponse bias low? |  |  |  |
|  | 4.5. Is the statistical analysis appropriate to answer the research question? |  |  |  |
| 5. Mixed methods | S1. Are there clear research questions? |  |  |  |
|  | S2. Do the collected data allow to address the research questions? |  |  |  |
|  | 5.1. Is there an adequate rationale for using a mixed methods design to address the research question? |  |  |  |
|  | 5.2. Are the different components of the study effectively integrated to answer the research question? |  |  |  |
|  | 5.3. Are the outputs of the integration of qualitative and quantitative components adequately interpreted? |  |  |  |
|  | 5.4. Are divergences and inconsistencies between quantitative and qualitative results adequately addressed? |  |  |  |
|  | 5.5. Do the different components of the study adhere to the quality criteria of each tradition of the methods involved? |  |  |  |

**S1.8 Authority, Accuracy, Coverage, Objective, Date, Significance (ACCODS) checklist for grey literature appraisal**

| **Dimension** | **Criteria** | **Author (year)** |
| --- | --- | --- |
| **Authority** | **Individual author** |  |
|  | Associated with a reputable organization? |  |
|  | Professional qualifications or considerable experience? |  |
|  | Produced/published other work (grey/black) in the field? |  |
|  | Recognized expert, identified in other sources? |  |
|  | Cited by others? (use google scholar as a quick check) |  |
|  | Higher degree student under “expert” supervision? |  |
|  | **Organization or group** |  |
|  | Is the organization reputable? |  |
|  | Is the organization an authority in the field? |  |
|  | **In all cases** |  |
|  | Does the item have a detailed reference list of bibliography? |  |
|  | **Dimension point score** | **/9** |
| **Accuracy** | Does the item have a clearly stated aim or brief? |  |
|  | Is so, is this met? |  |
|  | Does it have a stated methodology |  |
|  | If so, is it adhered to? |  |
|  | Has it been peer-reviewed? |  |
|  | Has it been edited by a reputable authority? |  |
|  | Supported by authoritative, documented references or credible sources? |  |
|  | Is it representative of work in the field? |  |
|  | If no, is it a valid counterbalance? |  |
|  | Is any data collection explicit and appropriate for the research? |  |
|  | If item is secondary material (e.g. a policy brief of a technical report refer to the original, and determine if it is an accurate, unbiased interpretation or analysis? |  |
|  | **Dimension point score** | **/11** |
| **Coverage** | All items have parameters which define their content coverage. These limits might mean that a work refers to a particular population group, or that it excluded certain types of publication. A report could be designed to answer a particular question, or be based on statistics from a particular survey.  Are any limits clearly stated? |  |
|  | **Dimension point score** | **/1** |
| **Objective** | It is important to identify bias, particularly if it is unstated or unacknowledged.  Opinion, expert or otherwise is still opinion: is the author’s stand point clear? |  |
|  | Does the work seem to be balanced in presentation? |  |
|  | **Dimension point score** | **/2** |
| **Date** | For the item to inform your research, it needs to have a date that confirms the relevance  Does the item have a clearly stated date related to content? No easily discernible date is a strong concern.  If no date is give, but can be closely ascertained, is there a valid reason for its absence? |  |
|  | Check the bibliography: have key contemporary material been included? |  |
|  | **Dimension point score** | **/2** |
| **Significance** | This is a value judgement of the item, in the context of the relevant research area  Is the item meaningful? (this incorporates feasibility, utility and relevance) |  |
|  | Does it add context? |  |
|  | Does it enrich or add something unique to the research? |  |
|  | Does it strengthen or refute a current position? |  |
|  | Would the research area be lesser without it? |  |
|  | Is it integral, representative, typical? |  |
|  | Does it have impact? (in the sense of influencing the work or behaviour of others) |  |
|  | **Dimension point score** | **/7** |
| **Total points rating** |  | **/31** |
