# Supplementary material for: Expanded roles of community health workers beyond malaria in the Asia-Pacific: A systematic review
Source: PLOS Glob Public Health. 2024 Oct 16;4(10):e0003113. doi: 10.1371/journal.pgph.0003113 (PMC11482702; doi:10.1371/journal.pgph.0003113)
Supplement: S5 Appendix — (DOCX) [file pgph.0003113.s005.docx]

**S2 Appendix.** Results Supplementary Information.

**S2.1 Description of roles and/or services provided by CHWs**

| **Roles and/or services** | **Description of service** |
| --- | --- |
| **Malaria Services** | |
| Treatment | Provide malaria treatment with antimalarial; and/or provision of primaquine for radical treatment of P. *vivax* (KHM-III) |
| Testing | Provide unspecified type of malaria test; malaria testing with RDTs or by obtaining blood smears for microscopic diagnosis, or both. |
| Referral | Referral of malaria cases including children, pregnant women, and/or severe cases; and/or referral to health center for G6PD testing |
| Provision/promotion of preventative measures | Promote the use of insecticide-treated bed nets (ITNs) and/or long-lasting insecticidal nets (LLINs); survey the use of mosquito nets; distribute and impregnate mosquito bets; distribute larvicides; and/or perform indoor residual spraying (IRS) |
| Surveillance, case reporting | Perform active case detection, surveillance activities, case reporting, and follow-up of cases in the community |
| Education and mobilization activities | Provide consultation, health education and engage in mobilization campaigns for malaria including taking on the role of health educator within the community |
| Patient follow-up | Follow-up patient to monitor adherence to treatment schedule |
| Early diagnosis and treatment | Early diagnosis and treatment |
| Active Case Detection | Active detection of malaria infections within the community and households that are of high risk |
| Integrated Vector Management | Integrated vector control activities |
| **Non-malaria services (health promotion activities)** | |
| Immunisation and vitamin A supplementation | Promote, assist and/or maintain records of immunizations, vaccination campaigns and provide combination preventative treatment such as vitamin supplementation and deworming |
| Antenatal care (ANC) and mother and child health (MCH) | Provide information on nutrition for pregnant women and infants, safe pregnancy, facility-based MCH services including institutional delivery, birth spacing, childbirth preparedness, care in postpartum period, and breast-feeding practices |
| Sensitisation and mobilisation activities | Prevent and promote health activities through health education and dissemination of health messages; conduct health mobilization activities such as community groups |
| Family Planning | Provide education on family planning and information on contraceptives |
| Sanitation; personal, food and water hygiene | Promote sanitation, food and water hygiene, and personal hygiene |
| Nutrition | Provide counselling on nutrition and promotion of positive health |
| HIV/AIDs and STIs | Provide health education on human immunodeficiency virus (HIV) and/or sexually transmitted infections (STIs) |
| Family Welfare | Unspecified |
| **Non-malaria services (direct patient care services)** | |
| Diarrhoea | Treat diarrhoea and provide ORS and zinc |
| Tuberculosis | Identify or screen cases, active case finding; perform follow-up and directly-observed therapy, and refer cases |
| Antenatal care (ANC) and mother and child health (MCH) | Provide antenatal care, delivery care, postnatal care, newborn care and other birthing complications |
| Minor or unspecified common illnesses | Carry first aid supplies, multivitamins, and/or provide first aid for wounds/injuries, eye care, and carry medications for treatment of common illnesses such as snake bites, vomiting, skin infections |
| Family planning | Provide or assist in acquisition of contraceptives |
| Acute respiratory infections (ARIs) | Diagnose, treat, or refer cases with respiratory infections |
| General case referral to the health centres | Refer cases to health facility, including assisting in health check-ups |
| Leprosy | Identify and refer cases with leprosy |
| Fever | Treat or refer cases with fever |
| Dengue | Dengue surveillance, including detection of suspected dengue case and respond to outbreak, and detection using multiplex devices or two-lined and five-lined RDTs that also identified dengue virus (KHM-IV), or dengue antigen-antibody RDTs and multiplex biosensors that also identified dengue virus (KHM-V). |
| Anemia | Provide iron/folic acid for anemia |
| Pneumonia | Treat or refer cases with pneumonia |
| HIV/AIDs and STIs | Recognising, and referring to a health centre, patients with human immunodeficiency virus (HIV) and/or sexually transmitted infections (STIs) |
| Child development and malnutrition | Monitor children growth and development, detect cases using mid-upper arm circumference measurement, treatment with ready-to-use therapeutic food, and/or refer cases with malnutrition |
| CCM, IMCI, iCCM | Community case management (CMM) of acute childhood illness (pneumonia, diarrhea, and malaria in endemic areas), and referral of complicated cases. Integrated management of childhood illnesses (IMCI) including of fever, diarrhoea, and ARI interventions, providing basic medications, and referral of severe cases to health centers. Integrated community case management (iCCM) delivering treatment and services to children under five including for malaria, pneumonia, diarrhoea, and malnutrition. |
| Non-communicable diseases | Identify, refer cases and/or supervise treatment for common NCDs, including diabetes, hypertension, mental disorders |
| Lymphatic filariasis (LF) | Unspecified |
| Other infectious diseases | Early detection and treatment of Chagas disease (PAK); use of multiplex devices or two-lined RDTs detecting dengue and Burkholderia pseudomallei, and five-lined RDT detecting dengue, B. pseudomallei, Plasmodium vivax and falciparum, Yersinia pestis (KHM-IV); and, use of multiplex biosensors for malaria (four Plasmodium species, using pLDH and HRP2), dengue virus, zika virus, chikungunya virus, leptospirosis, Rickettsia typhi, Burkholderia pseudomallei, and Orientia tsutsugamushi (KHM-V) |
| Visceral leishmaniasis (VL) | Identified probable case and refer |
| Covid-19 | Identify, treat, and follow-up COVID-19 cases; monitoring quarantine and distribution of supplies |
| Environmental and occupational health | Unspecified (IRN); treat sciatica. |
| G6PD testing | Glucose-6-Phosphate Dehydrogenase (G6PD) measurement using biosensor. |
| Unspecified infectious illness | Provide early recognition, diagnosis and supervised treatment for infectious diseases. |
| **General non-direct patient care activities** | |
| Collect, report and/or record health data | Collect, report and/or record health data including for births, maternal deaths, and deaths; conduct population survey, health risk assessment, and/or CHW evaluations |
| Household visits | Conduct home visits for various health programmes, disease surveillance activities, and/or to follow-up on absent patients |
| Disease surveillance | Participate in surveillance activities for various diseases such as for COVID-19, polio, chronic fever, and/or skin lesion |
| Respond to outbreaks of infectious disease and emergencies | Respond to outbreaks for infectious diseases and emergencies, including diarrhoea, measles, and/or influenza-like illnesses |
| Managing supplies | Maintain equipment, supplies, and/or medicines |

**S2.2 Extraction on evidence on impact or success of the identified programmes**

| **Programme/Description** | **Study/Report and programme outcomes** |
| --- | --- |
| **AFG**  The Afghanistan CHW program is part of the community-based health care (CBHC) component of the Basic Package of Health Services (BPHS) developed in 2003. CHWs provide a comprehensive set of services from health promotion to provision of health services and referral at a Basic or Comprehensive Health Center. CHW notable role includes their capacity to carry out community case management of acute childhood illness (pneumonia, diarrhoea, and malaria, where malaria is endemic), treatment of patients diagnosed with tuberculosis , and provision of family planning commodities. | **Aitken et al,** 2020, **Case Study**  To describe and provide insights into challenges and opportunities of the CHW programme in Afghanistan  **Malaria-related outcomes**  None  **Non-malaria related outcomes**  Changes observed between 2003-2013: 1. Decrease in maternal mortality ratio from 1,600 to 661 per 100,000 live births 2. Decrease in under-five mortality rate from 257 to 55 per 1,000 live births 3. Decrease in total fertility rate from 6.7 to 5.3  **Programme uptake and performance**  Based on the Afghan Mortality Survey in 2010, there were improvements in utilization of services and health status compared to the levels observed in 2003:  1. Contraceptive prevalence rate (CPR), 20% vs. 8.5% 2. Total fertility rate, 5.1 vs 6.7  3. 68% vs. 8% of women had obtained ANC  4. 34% vs.14% of births were attended by a skilled birth attendant 5. 64% of children with diarrhoea were given oral rehydration solution (ORS) or safe home fluids  6. 64% of children with symptoms of pneumonia were given antibiotics  It was found that in 2014:  7. CHWs treated 30% to 36% of all cases of childhood ARI and diarrhoea recorded by the HMIS 8. 55% of women reportedly using short-term contraceptive methods are supplied by CHWs  9. Rates of ANC, skilled birth attendance, and immunizations have increased Note: While CHWs and FHA groups have contributed to the aforementioned improvements, the presence of a female health worker in most health facilities was also essential 9. Drop-out rate of CHWs was consistently less than 8% per year, possibly due to 1) updated training courses and requests for training in new skills (e.g. first aid training delivered in collaboration with WHO for 2,000 CHWs in 23 provinces), and 2) more supervisory visits in 2011 (94% of a sample of CHWs had received three or more supervisory visits by community health supervisors with 83% written records and recommendations present for these visits). |
|  | **Kelly et al.,** 2022, **observational study**  To understand the reach of the CHW programme in Afghanistan, assess the availability of CHWs in remote and non-remote districts across Afghanistan, describe the provision of services by CHWs, and to assess the effect of remoteness on CHWs visit during the antenatal period and postnatal period.  **Malaria and non-malaria related outcomes**  None  **Programme uptake and performance**  The following were observed differences between services provided by CHWs in remote (13,482 CHWs or 87% of total CHWs) and non-remote districts (2080 CHWs or 13%):  1. There was no difference between CHWs’ mean services provision of malaria referral or treatment between remote and non-remote districts  2. There were more referrals to health facilities for normal deliveries in remote areas  3. Ratio of CHWs supplies per pregnant woman higher in remote areas  4. There was more referral and treatment for diarrhoea, ARI and malnutrition for children in remote areas. |
| **BNG**  Malaria Control Program is part of the Health, Nutrition and Population programmes (HNPP) managed by BRAC. The HNPP is aimed at promoting a broad concept of health among disadvantaged communities through the deployment of frontline community health workers (Shasthya Shebika or SSs and Shasthya Kormi or SKs) who provide a broad range of health services, promotes health, creates demand and links community with government and private health sectors. Under the MCP, BRAC works with the NMCP to strengthen national malaria control activities either directly or through other NGOs (20 sub-recipients of the global fund). MCP goal is to reduce overall burden of malaria (morbidity and mortality) by 60% (2008 baseline year) in 10.9 million population from 13 high endemic districts by 2015. | **BRAC,** 2013, **Programme Report**  To provide an overview of BRAC's HNPPs including the MCP. Report discusses programme coverage (27 Upazilas or sub-district and population of 2.18 million), distribution of Long Lasting Insecticidal Mosquito Nets (LLIN) (120,100 LLINs), and Malaria patients treated (11,428), as of 2013  **Malaria and non-malaria related outcomes**  None  **Programme uptake and performance**  Report published malaria case management indicators between 2008 - 2013 in 27 Upazilas as part of MCP directly managed by BRAC: 1. 89,354 Blood slide examinations  2. 55,465 RDTs 3. 11,569 Malaria case diagnosis  4. 11,428 Malaria treatment by community service providers  5. 15 Severe malaria referred  6. 120,100 LLINs distributed and in use For the EHC (essential health care) programme which covers 64 districts but is also delivered by SK and SS frontline community health workers managed by BRAC:  1. 3,089,891 Group health education forums organized  2. 172,728 Slab latrines installed 3. 846,368 Children under 1 year of age fully immunized 4. 783,794 Pregnant women received TT 5. 3,909,235 Common ailments treated by SS 6. 6,360,085 Modern contraceptives accepted 7. 497,291 Four or more antenatal care delivered 8. 619,716 Skilled attendance at birth 9. 195,700 Patients referred by SS for curative care 10. 1,123,642 Sputum microscopies for TB 11. 164,785 Symptomatic malaria cases tested |
| **IND-I**  Accredited social health activist (ASHAs) are village level health volunteers, introduced under National Rural Health Mission (NRHM) in 2005. ASHAs are usually a female local resident, engaged for local/peripheral healthcare services, mainly for maternal and child health care, and have later on been recruited by other communicable diseases programmes. ASHAs in many high-malaria endemic areas have been provided trainings for diagnosis and treatment of malaria. | **NHSRC,** 2011, **Programme report**  Structured interviews and questionnaires were implemented to answer the following objectives of the programme evaluation:  1. Understand the evolution of the ASHA programme, including stakeholder perspectives and experiences  2. Understand the characteristics of the ASHA, their beneficiaries, support structure and assigned roles  3. Assess ASHA's work outputs and attributable health outcomes (including effectiveness of programme in changing health behaviour, utilization of health services and responses to community's felt needs)  4. Review quality of key processes and mechanisms that constitute the programme  5. Use findings and recommendations of the evaluation to provide feedback to key stakeholders to strengthen the programme  6. Assess the extent to which such evaluations strengthen programme implementation  **Malaria and non-malaria related outcomes**  None  **Programme uptake and performance**  1. With regards to functionality (determined by ASHA motivation, system encouraging work, and signal for service uptake), functionality for malaria roles among ASHA is modest except in Orissa and Andhra Pradesh, with less than half of ASHA reporting functionality 2. In terms of work ASHAs self-reported as having done in last six months: only four districts (2 from Orissa) reported over 60% activity level for malaria control activities  3. Knowledge that blood smears need to be made to diagnose malaria were reported by over 85% of ASHAs in malaria endemic states; but knowledge of drug choice for malaria ranges is much lower. |
|  | **Gopalan et al,** 2012, **Mixed method study**  1. Assess the current level of performance motivation among the ASHAs  2. Understand factors affecting ASHAs’ level of motivation  3. Understand ASHAs' perceptions and experiences on the current status of the factors affecting their performance motivation  **Malaria and non-malaria related outcomes**  None  **Programme uptake and performance**  1. ASHAs are more motivated by the individual and the community level factors than the health system determinants 2. Qualitative findings support the survey outcomes that the healthcare delivery status and the human resource management modalities for ASHAs are not satisfactory  3. CHW management needs changes to ensure adequate supportive supervision, skill and knowledge enhancement and enabling working modalities. |
|  | **Das et al,** 2014, **Intervention Research Study**  This study tested the effectiveness of two service delivery models for reducing malaria burden, e.g. supportive supervision of community health workers (CHW) and community mobilization in promoting appropriate health-seeking behaviour for febrile illnesses in Odisha, India.  **Malaria and non-malaria related outcomes**  None  **Programme uptake and performance**  1. Significant improvements observed in the reported utilization of bed nets in both intervention models compared to control  2. Treatment-seeking from a CHW was higher in both intervention models compared to control 3. Fever cases were significantly more likely to visit a CHW and receive timely diagnosis of fever in the combined intervention models than in control  4. Care-seeking from trained providers increased with a substitution away from untrained providers  5. Fever cases from the supervision model and the community mobilization model were more likely to have received treatment from a skilled provider within 24 hours than fever cases from the control  6. Women from the intervention models were more likely to have received timely treatment from a skilled provider |
|  | **Scott et al,** 2020, **Case study**  To describe and provide insights into challenges and opportunities of the CHW programmes in India  **Malaria and non-malaria related outcomes**  None  **Programme uptake and performance**  Did not provide analysis but report on ASHA impact from other reviews/evaluation studies: 1. ASHA programme was associated with improvements in neonatal health, some aspects of care-seeking, and increased immunisation and health-related awareness such as 17% increase in receiving at least one ANC visit and 28% increase in facility births 2. ASHA programme provided wider coverage for lower income individuals |
|  | **Sonal,** year not known, **Programme brief**  1. Describe outcomes of ASAHs' involvement in malaria control programme in India  2. Describe challenges for increasing their involvement  3. Suggest way forward to utilize ASHA for malaria control  **Malaria and non-malaria related outcomes**  None  **Programme uptake and performance**  The National Vector Borne Di1.sease Control Programme indicated that as an outcome of ASHA involvement:  1. Over 10 million fever cases have been tested at the community level  2. About ten thousand Pf cases are being detected within the same day  3. >10 Million LLINs have been distributed during 2009-2011, protecting 25 million population in high endemic areas from malaria |
| **IND-II**  The goal of the Mandla Malaria Elimination Demonstration Project (MEDP) was to demonstrate that the elimination of malaria from 1,233 villages of district Mandla was possible using robust surveillance, case management, and vector control. To interrupt the local transmission of malaria in Mandla district (stop locally transmitted malaria), the MEDP uses evidence-based proven strategies of case management (rapid diagnosis and prompt treatment of malaria case), integrated vector management (IRS, minor engineering, LLINs), robust surveillance system and appropriate Information Education Communication (IEC) and Behaviour Change Communication (BCC) strategies. The project draws on a multitude of CHWs including VMWs and MFCs, in conjunction with other traditional vector borne disease control affiliated CHWs such as ASHAs, MPWs, ANMs. | **Rajvanshi et al,** 2020**, Case study**  To describe the study design and operational framework for a malaria elimination model piloted in Mandla district, Madhya Pradesh, India.  **Malaria related outcomes**  1. Between June 2017 to May 2020:  - MEDP reported a reduction of 91% indigenous malaria cases in Mandla district  - Malaria positivity rate fell from 0.33% in 2017–18, 0.13% in 2018–19 and 0.06% in 2019–20, to 0.18% malaria positivity in September–October 2018, followed by 0.06% in June 2019, and 0.03% in December 2019  **Programme uptake and performance**  None |
|  | **Rajvanshi et al,** 2021b, **Case study**  This case study presents a model for malaria elimination with district as an operational unit, which may be considered for malaria elimination in India and other countries with similar geography, topography, climate, endemicity, health infrastructure, and socio-economic characteristics.  **Malaria related outcomes**  Between June 2017 to May 2020:  1. MEDP reported a reduction of 91% indigenous malaria cases in Mandla district  2. Several rounds of mass screenings were conducted and revealed 0.18 % positivity in Sep-Oct 2018, followed by 0.06 % in June 2019, and 0.03 % in December 2019, and these were mostly asymptomatic cases in the community  3. The district has a total of 297 sub-centres, out of which, in the year 2017, 143 sub-centres were free of malaria, which increased to 198 in 2018, and 211 in 2019.  **Non-malaria related outcomes**  None  **Programme uptake and performance**  1. Significant improvement in the mosquito knockdown rates and usage of LLINs was observed following monitoring of vector control measures (IRS and LLIN) by MEDP  2. The project has trained over 422 field staff and achieved qualifying rate of 94.3% after a single training session  3. Significant and progressive improvement in scores of existing and new staff has been noted with techniques such as ‘shadowing’ and introduction of new monitoring tools (30-point checklist)  4. Using the Solution for Community Health-Workers (SOCH) mobile application, the project enrolled the entire population of the district (1.15 million) and undertook a detailed study on the socio-economic factors affecting malaria and found association between malaria cases and different household variables, such as age, gender, number of members, number of rooms, caste, type of house, toilet facilities, water supply, cattle sheds, agricultural land, income, and vector control interventions  5. SOCH mobile application improved the stock accountability by 60%  6. SOCH mobile application improved adherence to the Advance Tour Plans (detailed work plans for VMWs to follow in conducting surveillance activities) from 62–95%  7. During the 9 months of rolling out the SOCH application which moved reporting to an online platform, the initial difference in reporting between paper-based system and mobile-app systems was 49%, which was reduced to 0.4% with regular troubleshooting |
|  | **Rajvanshi et al,** 2021a, **Qualitative study**  This Knowledge, Attitudes and Practice (KAP) study sought to assess the health literacy and malaria-related training needs of ASHAs within the Mandla district.  **Malaria related and non-malaria related outcomes**  None  **Programme uptake and performance**  1. 91.8% ASHA reported using RDTs, with 85% reporting the use of blood slides  2. Only 1 out of 220 ASHA could interpret various test results of bivalent Pf and Pv malaria RDT correctly, although 87% correctly identified negative results. 15% ASHA correctly recognized Pv positive case while 11% can identify Pf. 60% ASHA can identify mixed infections correctly; incorrect and invalid test were correctly identified by 51% and 33% ASHAs respectively  3. Duration ASHAs took to interpret RDT results varied. 41.4% ASHA took 5 minutes, 46.8% took 15 minutes, 8.6% took 30 minutes, and 3.2% did not know how to answer the question  4. 8.2% ASHAs report diagnosing a patient based on symptoms, and 1.4% reported not knowing how to diagnose malaria  5. With regards to drug stocks, 19.1% ASHAs reported not having any RDTs for diagnosis and 47.7% reported not having ACT for treatment of Pf malaria (the dominant infection in the district)  6. With regards to malaria treatment choice, 85% ASHAs used chloroquine, 44.5% used artemisinin-based combination therapy (ACT), and 55.5% used primaquine. Only 38.2% ASHA gave PQ for 14 days in cases of Pv.  7. ASHA showed a lack of knowledge about the correct anti-malarial to use. ASHAs could not correctly recognize the age group-wise colour packs of ACTs, and all ASHAs did not have adequate literacy levels to read the age-groups on the packs (although about 25% ASHAs could recognize one correct ACT pack colour corresponding to the age group)  8. KAP assessment found 97.7% ASHA knew that mosquitoes were the main spread of malaria, but 30% also believed that malaria can be spread by drinking bad water and 5% had other misinformation about how malaria is spread. |
|  | **Rajvanshi et al,** 2022, **cross-sectional study**  To assess the capabilities of ASHAs and ANMs of Mandla district to diagnose and treat malaria as part of MEDP program in the tribal district of Mandla  **Malaria and non-malaria related outcomes**  None  **Programme uptake and performance**  KAP assessment found that ASHA and ANM had knowledge about the following:  1. 93.42% ASHA and 98.52% ANMs know that Mosquito bites are the transmission vector  2. Both groups report misconceptions about fresh running water and garbage as the breeding source of malaria mosquito  3. ANM (60.29%) were better at correctly identify infants and pregnant women as high risk group for malaria than ASHAs (22.37%)  4. Both groups identify mosquito nets as the preventative measure against malaria  5. Accuracy of interpreting RDT results by ASHA is higher in this study compared to baseline survey (Rajvanshi 2021a)  6. ANM had better understanding of malaria treatment according to national drug policy |
| **IND-III**  The Mitanin program aimed to improve access to health care and the health status of rural communities in Chhattisgarh state in India through the employment of Mitanin female health workers. | **Chourasia et al,** 2018, **Mixed-method study**  1. Evaluate and compare the knowledge and quality of malaria surveillance work performed by Mitanins’ engaged with Implication of Insecticide Resistance (IIR)-India project in comparison to Mitanins not involved in the project activities (Non-IIR)  2. Explore the effect of various socio-demographics factors (e.g. age and education) on malaria surveillance work of the study population  3. Find the various challenges faced by the Mitanins to perform routine malaria surveillance activities.  **Malaria and non-malaria related outcomes**  None  **Programme uptake and performance**  1. IIR-Mitanins’ (who received training on malaria surveillance) performance was significantly better than non-IIR group  2. The performance factors were basic educational qualification, level of knowledge about malaria sign and symptoms, knowledge about malaria control, slide preparation, RDT preparation, knowledge about anti-malarial drugs, treatment of Pv Treatment of Pf, form filling and documentation, and temperature reading |
|  | **Ranjha et al,** 2022, **Qualitative study**  To assess the knowledge gaps and skills of Mitanins in Chhattisgarh, and identify factors to improve their performance for malaria control and elimination  **Malaria and non-malaria related outcomes**  None  **Programme uptake and performance**  1. 60% of mitanins had an average KAP score, about 26% had good KAP scores, and 13.3% had poor KAP scores.  2. For malaria, diagnosis, prevention, and treatment section, the frequency of mitanins with the low scores was high, 76.8%, 35%, and 24.1%, respectively. Documentation and basic malaria were the sections where the mitanins scores were high  3. KAP score most affected by education factor, level of endemicity |
| **IND-IV**  Village Health Guide (VHG) Programme aims to align medical training with the country’s needs and establish guidelines for a new group of health workers and improve coordination between Multipurpose Health Workers and medical officers. The programme also target to increase community access to preventive, promotive, and curative care services and create a link between rural communities and the formal health sector. | **Strodel et al,** 2020**, Case study**  To describe and provide insights into challenges and opportunities of the VHG programme  **Malaria and non-malaria related outcomes**  None  **Programme uptake and performance**  The case study reported challenges of the scheme as follows:  1.VHG training at local Public Health Care centers failed to provide them with the skills necessary to carry out their assigned responsibilities. Instructors training the VHGs were often unaware of the overall goals and methods of the VHG Scheme, and only one half of the VHG trainees received training manuals. Only 3% of VHGs received their medical kits during training, further impeding the learning of new skills  2. VHG curriculum included practical skills such as latrine building and water purification, but did not include education on broader social factors affecting health hindering their performance  Based on a survey conducted by the National Institute of Health and Family Welfare in 1979:  3. VHGs responded correctly to only 20–30% of questions about patient referral, disease prevention, and emergency care  4. VHGs responded correctly less than 30% of the time to questions assessing knowledge of disease prevention; conditions requiring referral to higher-level facilities; emergency treatments; and general preventive, promotive, and curative services  5. VHGs failed to constitute a link between the community and the PHC centers, referring on average fewer than two patients to the PHC centers every 2 weeks  6. A study in the Mysore district of Karnataka found that although 80% of VHGs correctly identified malaria symptoms, only 20% knew how to make oral rehydration solution to treat diarrhoea  7. Another study found 74% of the population thought that the most important task of the VHGs was to treat minor ailments, while only 0.4% said that community health education was most important  8. The VHG stipend (payments) shifted the role of the VHG away from that of a community advocate, educator, and link to the formal health system. Instead, VHGs’ duties were focused on basic curative care and tasks assigned to them by medical personnel  9. All evaluations carried out revealed a high level of contact of VHGs with the population they were serving and a high level of satisfaction with the curative care that they provided, hence showing high acceptability within the community |
| **IND-V**  Multipurpose Health Workers (MPWs) were the result of integrating various community health workers that have previously worked under vertical programmes in India (Malaria workers under the National Malaria Eradication Programme and Auxiliary Nurse Midwives under the Maternal and Child Health Programme, among others). Health structure in each state varies; within Kerela, MPWs (one male and one female) are the primary functionary at a health subcentre which is the first level of contact of the community with the health care system. | **Nair et al, 2001, Mixed methods study** 1. Evaluate the work performance of the MPWs in the provision of basic health care and in the implementation of national health programmes  2. Examine the relevance of the current job responsibilities of MPWs in the context of the felt needs of the community and with regard to actual time spent by the workers on each of these diverse activities  **Malaria and non-malaria related outcomes**  None  **Programme uptake and performance**  Wider coverage of care provided by MPWs reduces unequal access to care: there is evidence for negative impact as MPWs were found to over-report their performances in national programmes. The highest discrepancy between MPW claim of service provision and actual service delivered as estimated by household survey results was in providing malaria services |
| **IDN-I**  MiP-MCH or the Maternal and Child Health and Integrated  Malaria Control in Eastern Indonesia is an expansion of the MCH programme aimed to improve access to quality maternal and new-born care services in the four eastern provinces of North Maluku, Maluku, Papua and West Papua. The specific aim of MiP-MCH program is to reduce the rate of malaria in pregnancy and its consequences through malaria prevention, screening and early diagnosis and treatment among pregnant women. | **USAID-UNICEF,** 2017, **Case Study employing mixed-method study design**  1. Describe the MiP-MCH programme development, early and current implementation at the national level  2. Identify the current implementation, challenges and achievement in Jayapura and Maluku Tenggara Barat (MTB) as case studies  3. Identify the potential challenges and issues that should be addressed in the future programme implementation  **Malaria and non-malaria related outcomes**  None  **Programme uptake and performance**  1. Increased LLIN protection due to programme’s continuous year-long distribution system targeting vulnerable populations, pregnant women at first ANC visit and infants completing their basic immunization schedule  2. Improved access to malaria diagnosis and treatment of pregnant women: Malaria screening among pregnant women is highest in Jayapuraand MTB districts, showing annual coverage higher than 92% in Jayapura in 5-year period (with an exception of the 2013 - 73% coverage) and in MTB with coverage range from 73% to 97% (with an exception of the 2014 – 67% coverage). In Jayaupura and Maluku Tenggara Barat, village midwives trained to screen and treat malaria also extended malaria services to entire populations in remote places where they were based. They also provided malaria screening for children under five with fever  3. Strengthened health worker’ capacity for malaria control beyond health facilities to the village-level through teamwork and task sharing, and training on malaria management  4. Increased public awareness about the dangerous consequences of malaria |
| **IDN-II**  Posyandu is an integrated health care post established to achieve health equity in Indonesia and in remote areas engaging primarily women volunteers as Kaders working with approximately 100 children under 5. | **Bandzuh,** 2022, **qualitative study**  To investigate local knowledge, attitudes, and practices of mosquito-borne disease prevention  **Malaria and non-malaria related outcomes**  None  **Programme uptake and performance**  The study found a positive impact of the programme on malaria prevention using ITNs. |
| **IRN**  The Behvarz programme is the expansion of the 1980s health care reforms in Iran which established health houses or public health centers to serve local communities. The programme is intended to scale up CHWs or Behvarz group and develop a health care system that is capable of delivering priority services (services relating to elder health, youth health, communicable and non-communicable diseases) in addition to the original mandate that only covered MCH. | **Rahbar et al,** 2020, **Programme report**  To describe and provide insights into challenges and opportunities of the CHW programme  **Malaria related outcomes**  None  **Non-malaria related outcomes**  1. The Behvarz programme have been a fundamental element in the establishment of a strong public health care system in Iran. WHO state that health houses contributed to the sharp drop in mortality and increased life expectancy over the last decades. In rural areas, Behvarzs have contributed to narrowing the rural–urban gap in health status since the 1980s. 2. Infant mortality rate decreased to 30.2 per 1,000 live births in 2000 in rural Iran and to 27.7 in urban Iran (in 2017, the infant mortality rate in urban Iran was 11.3 and 15.8 in rural areas)  3. Burden of disease attributable to communicable diseases has decreased to 10%  **Programme uptake and performance**  1. Provision of services by the Behvarz increased the coverage for target groups, contributed to economic growth and improvement in literacy rate as well as environmental services such as access to safe water and sanitation in rural areas 2. Behvarzs and Moraghebe-salamats (another CHW group) screened 76 million people (92% of the total population) for COVID-19 over a two-month period, identified and referred suspected cases and trained others in contact tracing |
| **KHM-I**  The VMW project added child health services to malaria control services in 2009, wherein VMWs in 400 villages, who had previously been trained and started providing malaria control services in 2008, received additional training to provide child health services by 2009. This new approach was technically and financially supported by the World Health Organization and the Global Fund for AIDS, TB and Malaria (GFATM). | **Yasuoka et al,** 2012, **Qualitative study**  1. Examine whether the scale-up of the VMW project with the additional health services interfered with the VMWs’ original services to control malaria  2. To analyse VMWs’ overall achievements, perceptions, and knowledge regarding the additional health services performed  **Malaria and non-malaria related outcomes**  None  **Programme uptake and performance**  1. 66.3% VMW respondents became more enthusiastic about serving as a VMW since the scale-up, and all but one respondent reported being willing to continue providing the new services 2. Most VMWs’ knowledge areas (malaria epidemiology and vector ecology) showed significant improvement. |
|  | **Hasegawa et al,** 2013, **Cross-sectional study**  1. Identify determinants of caregivers’ VMW service utilization for childhood illnesses  2. Examine the association between VMWs’ service quality and caregivers’ knowledge of childhood malaria management  **Malaria and non-malaria related outcomes**  None  **Programme uptake and performance**  1. Among the caregivers, 23% in M villages (VMW perform malaria services only village) and 52% in M+C villages (VMW perform malaria and child health services villages) utilized VMW services for childhood illnesses  2. VMWs’ service quality and experience duration are important determinants for caregivers’ utilization of VMWs’ child health services and their knowledge of malaria management  3. Caregivers were found to seek VMWs’ support for childhood illnesses even if VMWs provide only malaria control services. |
|  | **Liverani et al,** 2017, **Qualitative study**  1. To understand care seeking behaviours of communities and factors promoting or discouraging the utilization of VMW services  2. Explore factors that promote or discourage access to VMW services in poor communities. With regards to expanding roles of VMWs, the study aims to understand:  3. Whether health volunteers should serve as a link between the community and the health system (e.g. through surveillance, case referral, health promotion) or be involved increasingly in care and treatment delivery (e.g. taking on a role and tasks conventionally performed by health professionals)  4. Whether future programmes should be driven by a vertical approach, focused on specific diseases or health concerns, or horizontal approach whereby health workers should provide a more comprehensive range of services  5. To assess knowledge about factors that may facilitate access to services  6. To assess acceptability of programmes among user communities.  **Malaria and non-malaria related outcomes**  None  **Programme uptake and performance**  1. Utilization and reception of VMWs: a majority of forest goers (n = 20/35) in the study sample visited the local VMW in the past, and some (n = 9/35) had done so during their latest febrile illness episode. In “eVMW” villages (villages with VMWs performing expanded roles), only a minority of caregivers (n = 3/16) used VMW services for their children.  2. Accommodation: VMWs are volunteers, and economic pressures may limit their ability to accommodate patients and turn to other economic activities such as farming or running a business  3. Accessibility (proximity to health facilities or VMW house): spoor infrastructures and distance between VMW household and community which they serve was reported to hinder accessibility  4. Availability (supply of services and products): VMWs were reportedly not used as a first point of consultation when patients or their caregivers were uncertain about the cause of disease. The need for a more comprehensive service and ability to diagnose and treat a variety of conditions were cited by all categories of interviewees as a reason to prioritise other providers over VMWs. Residents, including caregivers, prioritised clinics due to their ability to provide correct diagnosis when symptoms were not clearly attributable to a specific disease  5. Acceptability: reported high acceptability because VMWs were elected by villagers, after candidates were shortlisted by the village chief in consultation with the local health centre. However, a number of forest goers (n = 5) and caregivers (n = 4) were reluctant to consult VMWs as they had no professional skills or formal medical training. Interviews with other participants indicate that perceptions could change as trust and confidence can be built over time. |
| **KHM-II**  The Greater Mekong Subregion Elimination of Malaria through Surveillance (GEMS) project engages healthcare providers (clinics and pharmacies), community-based health providers (malaria workers and mobile malaria workers in Cambodia), and community-based outlets (grocery stores) for malaria case management, and promote active case detection for the hard-to-reach and at-risk populations. | **Masunaga et al,** 2021, **Qualitative study**  To evaluate how the community and private sector have been incorporated into the GEMS project in Lao PDR, Vietnam and Cambodia.  **Malaria and non-malaria related outcomes**  None  **Programme uptake and performance**  None |
| **KHM-III**  The objective of roll out radical cure research project is to explore the feasibility and acceptability of biosensor use by village malaria workers (VMWs) in rural Cambodian villages. | **Adhikari et al,** 2022, **qualitative-dominant mixed-methods study**  To evaluate the operational aspects of biosensor deployment by village malaria workers (CMWs) in Cambodia.  This was done by providing biosensors to VMWs and health centre-based lab technicians after training to perform G6PD tests among vivax malaria and febrile patients over the period of a year.  **Malaria and non-malaria related outcomes**  None  **Programme uptake and performance**  The quantitative questionnaire assessment demonstrated progressive improvement in participants (including 28 VMWs) skills in using biosensors |
| **KHM-IV**  This is a pilot project extending from the U.S. Defense Threat Reduction Agency Joint Science and Technology Office (DTRA-JSTO) program to develop novel point-of-need diagnostics for surveillance of emerging infectious diseases. Evaluation of the diagnostics in endemic countries began in 2014 in sites in Thailand, Australia, Sierra Leone, Cambodia, and Peru. In Cambodia, the pilot study recruited Village Health Worker (VHW) in per-urban area around Phnom Penh to assess the feasibility of self-administered RDTs. | **Morrison et al,** 2021, **Cross-sectional study, quantitative non-randomized**  To assess whether CHWs and community members in Cambodia and Peru can use 2 types of RDT tests (one that detected two pathogens, dengue and Burkholderia pseudomallei, and the other that detected five pathogens including dengue, B. pseudomallei, Plasmodium vivax and falciparum, Yersinia pestis) by observational FGD and scoring of their competency in each of the steps (12 and 15 steps for each respective RDTs).  **Malaria and non-malaria related outcomes**  None  **Programme uptake and performance**  1. Competency to use either test never reached 80%. Both CHWs and community members struggled to perform RDT tasks with the percentage of participants demonstrating high competency on their ability to execute the steps appropriately without asking for help, ranged from 26–76% and 23–72%, for the two-line and five-line tests, respectively. CHWs did slightly better than community member but differences were not statistically significant. Comparatively, in Iquitos Peru competency levels reached above 80%.  2. In Cambodia, no significant difference in ability to perform the test was observed between the two devices, between CHWs and householders, or by age. Only education level had a statistically significant effect. |
| **KHM-V**  This project explores the feasibility of expanded roles of village malaria workers (VMWs) in Cambodia, and is a collaboration with Mahidol Oxford Tropical Medicine Research Unit (MORU) and Action for Health Development Cambodia (AHEAD), supervised by the Cambodian National Center for Entomology, Parasitology, and Malaria Control (CNM). AHEAD oversaw field implementation from June to December 2022, providing VMWs in four districts of Battambang province with training the use of novel rapid diagnostic test kits for febrile illness and health education modules, evaluated primarily through qualitative methods. | **Betrian et al.,** 2023, **Qualitative study**  VMWs and health center personnel from 4 districts in Battambang province were trained on topics such as hygiene, disease surveillance, management of mild illness, and vaccination/antenatal care, and 19 focus group discussions (FGDs) were conducted to assess the acceptability and feasibility of expanding VMW roles in these topics.  **Malaria and non-malaria related outcomes**  None  **Programme uptake and performance**  According to self-reported data from the VMWs' logbooks, a total of 6,192 community members (59% female) received training on health topics from the VMWs. Differences were observed in the numbers of community members reached during subsequent dissemination of health topics by VMWs, with disease surveillance being the most frequently discussed topic. Vaccination and Antenatal Care (ANC) were the topics on which the fewest community members (n = 481/6,192) received education. |
|  | **Dysoley et al.,** 2024, **Meeting report**  This meeting report documents findings from a stakeholder meeting during which findings from the operational research project was reviewed in the context of current malaria elimination strategies. The project provided 105 VMWs were equipped with new rapid diagnostic tests, which included dengue antigen–antibody and combined malaria/C-reactive protein tests. They were also provided training in electronic data collection and participated in health education sessions covering topics such as hygiene and sanitation, disease surveillance, first aid, management of mild illness, and vaccination and antenatal care.  **Malaria and non-malaria related outcomes**  None  **Programme uptake and performance**  None |
| **LAO-I**  The village health volunteers (VHVs) constitute the most peripheral level of the public health care system in Lao PDR. VHVs provide primary health care services, including diagnosis and management of basic diseases (respiratory diseases, diarrhoea, and uncomplicated malaria) as well as providing health education, assist in vaccination campaigns and insecticide treatment of bed-nets, and reporting of morbidity and mortality data to health or district health offices. | **Phommanivong et al,** 2010, **Cross-sectional qualitative study**  To evaluate the effects of training on VHV's knowledge and practice of EDAT (early diagnosis and treatment, i.e. the practice of RDT and quality of ACT prescription) malaria national strategy which was initiated in three pilot areas in 2005. Performance of VHV in the practice of RDTs and prescription quality of ACTs were compared between VHVs who were enrolled in the pilot villages, versus VHVs that received training in the expanded phase of the project (non-pilot villages) between 2006-8.  **Malaria and non-malaria related outcomes**  None  **Programme uptake and performance**  1. Improved RDT practices: VHVs from Pilot Programme had an overall better score (12.2) than non-PP (9.1)  2. Dispensation of ACT by VHV from PP was better than VHV from non-PP. |
|  | **Napier et al,** 2021**, Qualitative study**  1. Describe the demographics of the CHW cadre and contributions to malaria case management in Lao PDR and Honduras  2. Document ongoing implementation of community case management programmes  3. Identify areas for network strengthening.  **Malaria and non-malaria related outcomes**  None  **Programme uptake and performance**  Overlap between CHWs of NMCPs and VHVs (performing other health services) improves receptivity of CHW and their services despite declining malaria |
|  | **Oo et al,** 2022, **qualitative study**  To inform a new community delivered malaria elimination model with recommendations of key health stakeholders, VHVs and community members.  **Malaria and non-malaria related outcomes**  None  **Programme uptake and performance**  None |
|  | **Liverani et al.,** 2024, **qualitative study**  To document prospects to develop current community-based health programmes, taking into account community perceptions regarding health and healthcare priorities, implementation challenges, the policy landscape and opportunities associated with emerging technologies.  **Malaria and non-malaria related outcomes**  None  **Programme uptake and performance**  None |
| **LAO-II**  The Greater Mekong Subregion Elimination of Malaria through Surveillance (GEMS) project engages healthcare providers (clinics and pharmacies), community-based health providers (village health volunteers), and community-based outlets (shop-based volunteer malaria workers in Lao PDR) for malaria case management, and promote active case detection for the hard-to-reach and at-risk populations. | **Masunaga et al,** 2021, **Mixed methods study**  To evaluate how the community and private sector have been incorporated into the GEMS project in Lao PDR, Vietnam and Cambodia.  **Malaria and non-malaria related outcomes**  None  **Programme uptake and performance**  1. Performance of Village health workers (VHW – the umbrella term used in this study) is impacted by frequent lack of diagnostics and/or treatment tools at their disposal  2. VHWs have to meet their own work requirements in addition to health provider role, making it difficult for care seekers to access at times  3. VHW role as "malaria worker" deters care seekers because the shift in their function to performing malaria work as well is viewed as limiting their competency in treating other illness |
| **LKA**  The Health Volunteer programme in Sri Lanka expanded from a movement supporting volunteers through various NGOs starting from a UNICEF supported volunteer programme in the 1950s that had primarily focused on health education and development. While the programme is valued, it was never formalized by the Ministry of Health due to lack of interest by the prevailing government to fund health programmes. | **Walt et al,** 1989**, Case study**  To understand the motivation of CHW cadres/volunteers and to consider the feasibility of relying on volunteers to support primary health care policies in Sri Lanka.  **Malaria related outcomes**  None  **Non-malaria related outcomes**  None  **Programme uptake and performance**  The conclusion is that large-scale community level volunteer programmes will be characterized by high attrition and low activity rates and will only be sustainable under particular enabling conditions.  The study reported lack of community engagement activities in this programme; but discussed that selection of CHWs by community members are important to make community members feel more comfortable with the health services and increase CHWs' motivation.  Case study suggests that to sustain CHW programmes, certain conditions need to be present:  1. Presence of young, educated men and women in rural areas where opportunity for further training or employment is limited  2. Cultural force and the associated religious or ethical values that values voluntary work  3. Political commitment  4. CHWs' continuing education, regular supervision and meetings with health staff may also be motivating forces |
| **MMR-I**  Population Services International (PSI) Myanmar started a social franchising programme with the Sun Quality Health network which focused on engaging private providers in urban and peri-urban settings in 2001, and in 2008, established the Sun Primary Health (SPH) network to increase rural coverage of priority health services including malaria prevention and treatment, reproductive health (RH) services, pneumonia diagnosis and referrals, diarrhoea prevention and treatment, and referrals to treatment facilities for tuberculosis (TB). SPH providers are typically auxiliary midwives, retired nurses, teachers, farmers, or housewives residing in the rural areas they serve. The SPH Network was expanded in 2011. | **Aung et al,** 2015**, Case Study**  1. Evaluate the effectiveness of a training programme for improving diagnostics and treatment quality of paediatric malaria by SPH providers 2. Assess whether any quality improvements were sustained over the following 12 months after training was delivered.  **Malaria and non-malaria related outcomes**  None  **Programme uptake and performance**  1. Average clinical quality scores for diagnosis and treatment of paediatric cases of newly recruited SPH providers prior to training (baseline) were 12/100. Six months after training, average quality scores were 48/100 2. 12 months after training, providers were retested and average quality scores were 45/100  3. 197 providers were added to the rural health workforce with a greatly improved capacity to test, diagnose, and treat malaria |
|  | **PSI,** year not known, **Programme brief**  To explore multiple models to scale‐up integrated community case management (iCCM) of pneumonia, diarrhoea and malaria for remote populations.  **Malaria and non-malaria related outcomes**  None  **Programme uptake and performance**  None |
| **MMR-II**  Medical Action Myanmar (MAM), a medical aid organisation, has been supporting a network of Village Health Workers (VHWs) in the most remote communities in Myanmar. The MAM VHWs initially provided malaria control activities exclusively but as malaria decreased rapidly in these communities, VHWs were trained to offer an extended basic health care (BHC) package. The expansion was initiated between 2013 and 2014 in an effort to meet health needs and ensure continued community uptake of malaria services provided by the CHWs. | **McLean et al**, 2018, **Observational study and interrupted time series analysis**  1. Assess the rates of decline in Plasmodium falciparum and Plasmodium vivax malaria incidence, RDT positivity, and monthly blood examination rates with each year of VHW operation  2. Investigate the effects of the addition of a BHC package on the uptake of malaria services in four cohorts of VHW which had provided malaria services only for at least 1 year prior to the expansion  **Malaria related outcomes**  Communities with VHWs providing malaria diagnosis and treatment experienced declines in P. falciparum and P. vivax malaria incidence of 70% and 64% respectively with each year of operation.  **Non-malaria related outcomes**  None  **Programme uptake and performance**  1. BHC package integration was associated with an immediate and sustained increase in blood examination rates 2. Increase in health seeking behaviours after including BHC package  3. Increase number of CHWs providing BHC package (from 43 in 2013 to 1,040 in 2016)  4. 284,658 BHC consultations provided by CHW:  - diagnosis of 14,509 pneumonia cases  - identification and referral of 6278 patients with suspected tuberculosis  - identification and referral of 859 patients for other severe disease causes |
|  | **Zaw et al,** 2023, **retrospective analysis**  This study analyses the impact of a specific subset of CHWs managed by MAM in hard-to-reach communities in Mon State, East Myanmar by Medical Action Myanmar. The goal is to assess the impact of CHWs delivering early diagnosis and treatment for malaria on the incidence and RDT positive rates of P*.f* and P.*v.*  **Malaria related outcomes**  1. Over an 8-year period, 172 CHWs served a population of 236,340, conducting a total of 260,201 RDTs.  2. Each year of CHW operation saw a 70% decline in the incidence of P.*f* (and mixed infections), with the RDT positive rate for P.*f* dropping from 2.80% to 0.004% between 2011 and 2018, equivalent to a 69% yearly reduction.  3. Incidence of P. *v* declined by 56% per year of CHW operation, with RDT positivity rates decreasing from 2.41% to 0.61% between 2011 and 2018, indicating a 53% reduction in P.*v* positivity rate per year of CHW operation.  4. Out of 54,961 patients tested during the final 2 years of the project, only 1 imported positive case of P.*f* was detected. |
| **MMR-III**  Between 2016 and 2017, Malaria Consortium piloted an integrated community case management (iCCM) approach for malaria in three townships in Sagaing, in partnership with the Ministry of Health and Sports, the National Malaria Control Programme (NMCP) and township health departments. Existing malaria volunteers (MVs), previously trained by the NMCP to diagnose and treat malaria in their communities, were given further training to diagnose and treat pneumonia and diarrhoea, and detect malnutrition in children under  five. | **Malaria Consortium (MC),** 2018, **Learning brief**  Evaluate iCCM training programme to improve MV motivation and promote the retention of MVs in areas where malaria transmission rates are declining.  **Malaria related outcomes**  None  **Non-malaria related outcomes**  None  **Programme uptake and performance**  1. 99% of MVs were correctly prescribing amoxicillin and 94 percent were correctly prescribing cotrimoxazole 2. 95% of MVs were able to count respiratory rates correctly, 98 percent could accurately identify severe signs of pneumonia, and 90 percent could correctly identify cases for referral 3. 91% of MVs were able to assess malnutrition using mid upper-arm circumference (MUAC) tape measurement and 90 percent could accurately use the weighing scale 4. Expansion of MV role alleviated basic health staff workload and motivated MVs to continue providing malaria services 5. 90% of trained MVs were still active by the end of the project |
|  | **Malaria Consortium (MC),** 2020, **Learning brief**  Discusses key lessons learnt from MC training of malaria volunteers to deliver iCCM in six townships in Sagaing region.  **Malaria related outcomes**  None  **Non-malaria related outcomes**  None  **Programme uptake and performance**  1. Report claims that the iCCM expansion successfully improved the management of common childhood illnesses in Sagaing region, with basic health staff noting that there were fewer complicated cases visiting the clinics as MVs provided services to 8,520 under-fives at the community level between 2017 and 2019 2. Focus group discussions found most MVs mentioned being highly motivated by their enhanced role and that they had earned their communities’ trust, who widely accepted iCCM  3. Targeted, responsive and regular supervision are key to the provision of sustainable and high-quality services  4. Monitoring supervision reports to determine appropriate frequency for supervision, and setting different frequencies for different settings depending on MV needs, also increases efficiency of BHS work. |
| **MMR-IV**  In 2016-17, national health authorities developed the integrated community malaria volunteer model (ICMV) that added services for other communicable diseases (dengue, lymphatic filariasis, tuberculosis, HIV/AIDS and leprosy) to maintain the social role of malaria volunteers or village malaria workers (VMW) as malaria incidence declines. The ICMV model succeeds the malaria volunteer model (vertical malaria programme) and was expanded nationally in 2018-19.  Across the country, different implementing partners manage ICMVs within their respective; however, these partners follow the NMCP ICMV manual. | **Davis et al,** 2020, **Case study**  To describe and provide insights into challenges and opportunities of the Community Based Health Workers (CBHW, another nomenclature referring to the range of CHWs in Myanmar) programme  **Malaria related outcomes**  None  **Non-malaria related outcomes**  None  **Programme uptake and performance**  1. According to the National Strategic Plan 2016–2020 for Malaria, Malaria Volunteers diagnosed and treated a total of 104,925 (57%) of the 182,616 malaria cases in 2015  2. Malaria volunteers trained to provide a broader, integrated package of services demonstrated an immediate and sustained increase in blood examination rates (McLean et al.. 2018) 3. Analysis of baseline-to-endline survey data demonstrated statistically significant higher coverage of antenatal care (72% versus 39%) and use of modern methods of contraception (24% to 45%) among other intervention coverage improvements 4. National Tuberculosis Programme noted significant contributions of volunteers in referrals and treatment, with 30,114 presumptive TB cases referred by TB Volunteers, resulting in detection and treatment of 5,130 cases in 2015 5. Roles of CBHWs supported improved outcomes in terms of Disability-Adjusted Life Years (DALYs), with 7,740 DALYs per 100,000 population averted per year, and is expected to increase to 9,708 DALYs per 100,000 population with the addition of family planning, nutrition, and pneumonia treatment |
|  | **Than et al,** 2020, **Case control and qualitative analysis**  1. Assess village health volunteers (another nomenclature of VMW) knowledge and performance before and after ICMV intervention (training of additional roles and integrating into ICMVs)  2. Identify challenges perceived by VHVs and inform how to improve support for ICMVs  **Malaria related outcomes**  None  **Non-malaria related outcomes**  None  **Programme uptake and performance**  1. Three months after the ICMV intervention (expanded roles training in 2017), knowledge scores for malaria *increased* from 48 to 63 points, with a difference in mean of 15 pts 2. Comparing the performance (completeness, consistency, timeliness and stock management) between the pre and post intervention:  - For malaria reporting status: the completeness of malaria reporting *increased* from 91% to 96%, the internal consistency (accuracy of data reported, no data discrepancies during measurement period*) increased* from 83% to 89%, but the differences were not statistically significant. Timeliness (newly diagnosed cases reported to a township malaria focal point within 7 days of month) increased from 85% to 98%, which was significantly different in the post intervention township  - For malaria commodities (measured as no stockout occurring for more than 1 week a month; expired RDT and antimalarials are considered stockouts): decrease in stockout of anti-malaria drugs were statistically significant in intervention township (although this is due in part to lack of positive cases and the need for treatment), but the decrease is not significant for RDTs 3. Malaria blood testing with RDTs was measured by recording the number of individuals tested with an RDT per month and the number of patients with a positive RDT per month:  - Average number of patients tested, or incidence of testing, in the intervention township *increased* from 4.3 tests per person-month to 7.7 tests per person-month after the intervention - Rate of testing (average number of test/month/VHV) in the intervention township was 1.56 times *higher* in the pre-intervention period than the comparison township - The rate-ratio of testing increased to 3.84 higher for intervention township than control township at post-intervention period which suggests that the expansion of ICMV roles did not have a negative impact on malaria outcomes  4. Three months after the ICMV intervention (expanded roles training in 2017), knowledge scores for all additional diseases (dengue haemorrhagic fever, lymphatic filariasis, tuberculosis, leprosy and HIV/aids) significantly increased |
|  | **Oo et al,** 2021a, **Qualitative study**  1. Explore the views of the MoHS and implementing partners (IP) on the current community-delivered malaria models in Myanmar  2. Explore MoHS/IP experiences in policy making and programme implementation of community-delivered models  3. Identify strategies that maintain and motivate the social role of volunteers in the community  4. Identify the preferred community-delivered malaria model  **Malaria related outcomes**  None  **Non-malaria related outcomes**  None  **Programme uptake and performance**  1. Performance of ICMVs were impacted by lack of financial and in-kind support that did not increase to match their increasing roles and responsibilities  2. ICMV faced issues in reporting non-malaria diseases because of lack of reporting forms  3. Uptake of ICMV services is impacted by lack of interest among villagers in ICMVs’ provision of sole-purpose health education |
|  | **Oo et al,** 2021b, **Qualitative study**  1. Explore the perspectives of community leaders and members, and malaria volunteers in South-East Myanmar regarding community-delivered models  2. Inform an optimal and contextualized model targeting malaria elimination in Myanmar  **Malaria related outcomes**  None  **Non-malaria related outcomes**  None  **Programme uptake and performance**  Community prefers midwives over VMW for non-malaria health services, and even for malaria services prefer basic health staff over VMW because they have better knowledge and facility. However, community still utilize VMW service as first point of contact because of accessibility. |
|  | **Oo et al,** 2021c, **qualitative study**  To qualitatively assess the sustainability prospects of the mobile case base reporting system (MCBR) system in the context of Myanmar’s malaria elimination program.  **Malaria and non-malaria related outcomes**  None  **Programme uptake and performance**  None |
| **MMR-V**  The Back Pack Health Worker Team (BPHWT) is a non-profit and multi-ethnic organization providing primary health care to ethnic groups and vulnerable populations in armed conflict and rural areas of Myanmar. BPHWT utilizes mobile health teams to provide a range of primary medical care, maternal and child health services, and community health education and prevention programmes. | **Mahn et al,** 2008, **Case study**  1. Describe key elements of the partnership model used to deliver health services by the BPHWT  2. Describe the role of BPHWTs and their contribution to improving the health of ethnic groups and vulnerable populations in rural areas of Myanmar.  **Malaria and non-malaria related outcomes**  None  **Programme uptake and performance**  1. In 2005, BPHWT reported diagnosis and treatment of common illness (malaria, ARI, diarrhoea and anaemia) with 93% and 86% accuracy; although authors noted that issues with diagnosing malaria was the most common challenge 2. BPHWT treated 78,000 cases through internally displaced persons (IDPs) service areas  3. Administered 43,000 doses of Vitamin A supplement and deworming treatments to children and postpartum women  4. Advocated for changes to the pollical and policy environments affecting IDPs through partnership and health information system at the international level (Thailand, UK, the UN) |
|  | **Backpack Health Workers Team (BPHWT),** 2010, **Programme report**  To report on BPHW activities, performance and achievements between 1998 and 2009.  **Malaria related outcomes**  1. Decrease in presumptive and confirmed malaria caseload from 17,404 in 2003 to 14,697 in 2009 2. Decrease in malaria rates from 2003-2009  **Non-malaria related outcomes**  None  **Programme uptake and performance**  1. Increased coverage of deworming and vitamin-A for children under 12 from 21% to 42% and 27% to 57%, respectively, between 2004 and 2009 (Note: statistics may be questionable due to difficulty in tracking the number of unique distributions to children in the target population)  2. Since 2015, installed 64 gravity flow water systems, 63 shallow wells, and at least 8,080 latrines in villages  3. BPHWs mass drug administration (MDA) coverage reached 71% of eligible lymphatic filariasis target population in 2009  4. Vitamin A was distributed to 3,348 pre-and postpartum women and de-worming medication was given to 3,281 pregnant women, which respectively denotes to about 53% and 52% coverage out of the total eligible population  5. Between 2007 and 2009 iron and folic acid has been distributed to approximately 3000-3500 pregnant women a year, which is roughly 55% of target population  6. According to a 2009 internal clinical logbook review, 94% of BPHWT pf patients were treated correctly according to clinical symptoms and treatment guidelines as outlined in Burma Border Guidelines 7. In 2019, BPHWs treated a total of 88,786 patients under the Malaria Control programme |
|  | **Backpack Health Workers Team,** 2019, **programme report**  To report on BPHW activities, performance and achievements  **Malaria related outcomes**  1. Decreasing malaria caseloads from 13,891 in 2010 to 3,596 in 2018  **Non-malaria related outcomes**  None  **Programme uptake and performance**  1. 3,596 malaria cases treated by the field health workers in 2018  2. 29,850 ITNs distributed in 2018 |
| **MMR-VI**  Better Health Together Project was launched by Community Partners International (CPI) aimed at strengthening and improving access to integrated health services in under-served and hard-to-reach communities in five states and regions of Myanmar. The project formalizes the coordination and cooperation between the MoHS and ethnic health organizations (EHOs) implementing ICMV programmes. | **Minn et al,** 2019, **cross sectional study**  To assess the extent of and factors associated with non-receipt of ‘correct’ and non-receipt of ‘correct and timely’ malaria treatment services provided by EHOs under the project.  **Malaria and non-malaria related outcomes**  None  **Programme uptake and performance**  1. Among 2881 people who were malaria positive (recorded by ICMV register maintained at health posts, mobile teams, and village-based ICMVs under the BHT project between 2017-18), 94.6% people received correct treatment, while 44.6% received correct and timely treatment  2. Among service delivery types, mobile team and village-based ICMVs were significantly more likely to provide correct treatment than health posts. |
| **MMR-VII**  The Malaria Elimination Taskforce (METF) Programme operates in 4 townships of Kayin state in Myanmar, supporting 1250 malaria since 2014 posts. These malaria posts are operated by malaria post workers who provide uninterrupted access to free early diagnosis and treatment of malaria to all fever patients. 42 of the 1250 malaria posts are based in a clinic or primary health care facility and therefore gave resources to manage non-malaria fever and offer inpatient services. | **Rae et al**, 2021, **observational study and regression analysis**  Investigates longitudinal trends in malaria testing rates, and impact of integrating malaria and non-malaria services on testing rates in METF programme in Kayin state of Myanmar using surveillance data and regression models.  Reported results are for Malaria Posts (which include the ICMW structure):  **Malaria related outcomes**  1. Incidence of Pf declined though this was not observed for Pv since 2014  **Non-malaria related outcomes**  None  **Programme uptake and performance**  1. From 2014-16 RDT rates declined but stabilised in 2016 (with an initial decrease in RDT rates from first to second year of malaria post opening, then for the consequent years, there were no significant difference between RDT rates between years. No observable impact of ICMW training on RDT rates up until 16 months after training was delivered to malaria posts in Hlaingbwe, 24 months for Kawkareik, and 19 for Myawaddy. As such authors conclude that the ICMW training seem to have no impact on RDT testing rates.  2. 72-83% of consultations occurred within 48 hr of fever onset (an indicator for malaria post service uptake is the delay between fever onset and malaria post consultation - which means a majority of consultations were conducted before positively diagnosed individuals became infectious therefore reducing onward malaria transmission)  3. On RDT quality control, recording of RDT results by the malaria post workers improved over time in all townships, sub-optimal RDT tests accounted for 1-7% in 2020 compared to 10-17% of RDTs checked in 2016. |
| **MMR-VIII**  The MOM Project is a collaboration between multiple organizations, training a three-tiered network of maternal health workers in basic emergency obstetric care, antenatal care, and family planning. Local health workers are trained centrally and then pass on their knowledge to second-tier local workers and third-tier traditional birth attendants. The goal is to expand coverage for essential maternal care by implementing basic interventions through less-trained providers and more complex ones through higher-trained providers, all delivered through mobile services at the village level. | **Mullany el at,** 2010**, two-stage cluster-sampling surveys**  To evaluate the impact of the MOM project on uptake of family planning, attendance at delivery by those capable of providing emergency obstetric care, and coverage of essential maternal health interventions.  Surveys utilizing two-stage cluster sampling were conducted among ever-married women aged 15–45, both before and after program implementation. These surveys aimed to assess alterations in the coverage of crucial antenatal care interventions, attendance at births by individuals trained to handle complications, postnatal care, and family planning services.  **Malaria and non-malaria related outcomes**  None  **Programme uptake and performance**  **Malaria:**  During implementation period, women whose latest pregnancy during this period were significantly more inclined to receive:  1. Malaria screening 55.9% compared to 21.9% at baseline with positive rates of 11.8% and 36.7% respectively  Or 2.53 (CI 2.01-3.18) times higher  2. Use insecticide treated net 59.3% compared to 21.6%  Or 2.75 (CI 2.19-3.45) times higher  **Non-malaria:**  During implementation period, women whose latest pregnancy during this period were significantly more inclined to receive:  1. At least one ANC visit during their last pregnancy 71.8% compared to 39.3% at baseline  Or 1.83 (CI 1.64-2.04) times higher  2. ANC visits 4 times or more 34.4% compared to 16.7% at baseline  Or 2.06 (CI 1.72-2.47) times higher  3. Blood pressure measured 72.9% compared to 43.1% at baseline  Or 1.69 (CI 1.51-1.86) times higher  4. Urine test 42.4% compared to 15.7% at baseline  Or 2.69 (CI 2.05-3.54)  5. Deworming treatment 58.2% compared to 4.1% at baseline  Or 14.18 (CI 10.76-18.71)  6. 90d Pe/Folic Acid 41.3% compared to 11.8% at baseline  Or 3.49 (CI 2.80-4.35)  Note: The administration of tetanus toxoid immunizations during pregnancy, not part of the MOM program package, showed lower rates during the MOM project period, although this difference did not reach statistical significance. When the analyses were redone with the baseline recall period limited to 2 years, the program's impact estimates remained similar. |
|  | **Teela,** 2009**, qualitative study**  To characterize and interpret the realities of reproductive health work in a conflict-affected setting.  **Malaria and non-malaria related outcomes**  None  **Programme uptake and performance**  None |
|  | **Mullany et al,** 2008**, programme report**  To describe the rationale, design and implementation of the project and a parallel monitoring plan for evaluation of the project  **Malaria and non-malaria related outcomes**  None  **Programme uptake and performance**  None |
| **NPL**  This is intervention research designed to determine the effectiveness of the integrated active case finding (ACD) and vector control interventions in Nepal through two intervention methods: fever camps or the utilization of Female Community Health Volunteers (FCHV). Primarily, FCHVs fall under the Family Health Division and provide Maternal and Child Health services, health education, communication and community outreach, while acting as a linkage between the health facility and the community. This group of workers do not traditionally perform malaria control activities nor provide health services regarding malaria. | **Banjara et al,** 2019, **Quantitative randomized control trial and Quantitative nonrandomized**  1. To compare the effectiveness of two different intervention strategies of integrated Active Case Detection: - Combined fever camps approach for ACD of VL/PKDL (Visceral Leishmaniasis / post-kala-azar dermal leishmaniasis) cases and other febrile and skin lesion disease including malaria, tuberculosis and leprosy implemented by mobile teams of health workers (rapid response team) in villages with VL cases reported in 2015 - Incentives based approach for ACD of the aforementioned diseases, implemented by FCHVs in villages with no VL cases in 2015 2. To determine the effectiveness of vector control interventions at the community level  **Malaria and non-malaria related outcomes**  None  **Programme uptake and performance**  1. The combined fever camps were attended by 398 people, and 275 were further tested: 79 for suspected VL, 97 for tuberculosis, three for leprosy and 99 for malaria. Of those tested, one each was positive for VL, leprosy and malaria, and three were tuberculosis positive. Blanket house-to-house screening, conducted immediately after the camp, found that of the 44,323 people from 7,211 households, an additional 679 chronic fever and 461 skin lesion cases were identified, although none of which was positive for the diseases in focus 2. Under the incentive approach, no case was reported by FCHVs during the year that followed their training to detect aforementioned diseases. Blank screening of 9,627 households was conducted 12 months after the training identified 11 cases of chronic fever and six skin lesions, but none tested positive for any of the diseases in focus. |
|  | **Paudyal et al,** 2022, **scoping review**  Too examine how community-based distribution of IFA supplements during pregnancy and to discuss future directions for sustaining coverage and achieving target on anaemia reduction.  **Malaria and non-malaria related outcomes**  None  **Programme uptake and performance**  1. Increase in % woman aged 15-49 who consume IFA supplements and anthelmintic treatment during their most recent pregnancy increased from 23% to 91% between 2001 and 2016  2. Increase in % woman aged 15-49 attending ANC from 49% to 94% between 2001 and 2016 |
| **PAK**  The Lady Health Worker (LHW) Programme was established to provide high quality integrated health services to underserved populations in rural and urban settings, and promote health and reduce poverty by bridging the gap between health services and communities. The programme draws on a group of LHWs attached to local public health centers but work from their homes and are primarily based within their communities. The programme was expanded in 2003 with the goal of improving quality of services and expanding coverage through increased deployment of LHWs across all five provinces in Pakistan. | **Lassi et al,** 2020, **Case study**  To describe and provide insights into challenges and opportunities of the CHW programme in Pakistan  **Malaria related outcomes**  None  **Non-malaria related outcomes**  None  **Programme uptake and performance**  1. Improvements in tetanus toxoid coverage, percentage of deliveries attended, percentage of children fully immunized, awareness among mothers regarding how to prepare oral rehydration solution for treatment of diarrhoea and level of exclusive breastfeeding  2. Population served by LHWs had substantially better health than the population without LHWs, including an 11% increased likelihood of using modern family planning and a 15% increase in immunization coverage among children younger than three years of age 3. The effect of LHW services was generally greatest in the poorest households 4. In villages where LHWs and trained birth attendants (TBAs) were linked and have received a brief training on new born care and service delivery, there were significant reductions in the number of stillbirths and neonatal mortality rate 5. Women in LHW programme service areas were 50% more likely to use modern reversible contraceptives than those who lived in areas without LHW services |
|  | **Sohail et al**, 2021**, qualitative study**  To explore the perceptions of LHWs and their trainers about their existing curriculum and identify gaps in the curriculum for effective implementation of the interventions identified in Essential Package of Health Services (EPHS).  **Malaria and non-malaria related outcomes**  None  **Programme uptake and performance**  None |
| **PNG-I**  The Home-based Management of Malaria (HMM) program managed by Population Services International (PSI) attempted to bridge the malaria service gap in three high burden provinces (East Sepik, East New Britain and Sandaun) using a network of volunteer community-based distributors (CBD). PSI selected provinces based on malaria incidence, access to other health facilities and level of support by the Provincial Health Administration. | **Lewinski et al,** 2018, **Programme brief**  1. Leverage lessons learned from malaria service delivery programmes to expand integrated community case management in remote areas of Papua New Guinea  PSI conducted a study to help generate evidence to expand HMM services to include all three integrated community case management diseases by:  2. Comparing treatment seeking behaviour by households among HMM and non-HMM communities  3. Assessing caregiver knowledge, attitudes and treatment seeking practices for diarrhoea and pneumonia  **Malaria related outcomes**  None  **Non-malaria related outcomes**  None  **Programme uptake and performance**  The primary finding of this research is that the HMM intervention has achieved many of its desired goals – primarily that caregivers in HMM intervention areas are much more likely to be aware of:  1. The CBDs working in their area  2. That CBDs can treat malaria, and select CBDs for the treatment of fever over other service providers  The findings suggest that there is substantial room to expand the clinical services provided by CBDs to include the additional  iCCM diseases, such as pneumonia and diarrhoea. |
| **PNG-II**  The Papua New Guinea National Health Plan 2011-2020 recognized that increasing access to prompt quality diagnosis and appropriate treatment needs to be a key intervention strategy to reducing malaria related mortality and morbidity. Village health volunteers or VHV, called a marasin meri or marasin man, who have been trained by NGOs to provide  primary health care services, including distribution of essential drugs (e.g. artemether/lumefantrine, amoxicillin, albendazole, acetaminophen, ferrous sulphate, folic acid and oral rehydration salt) and rapid diagnostic tests for malaria were identified as effective health service providers in their communities became the focus for achieving this national strategy. | **Inoue et al,** 2017, **Qualitative study**  This study draws on stakeholder surveys to investigate the association between VHV's social capital and caretakers' choice of healthcare provider for febrile children.  **Malaria and non-malaria related outcomes**  None  **Programme uptake and performance**  It was found that caretakers' choice of healthcare provider for febrile children depended on the social interaction (i.e. social capital) of VHVs and the various type of people inside/outside the village of which they work. VHVs with greater social capital through people in outside positions/roles was linked to more utilization of health services by caretakers. |
| **PHL**  The Republic Act No 760 or the 1991 Local Government Code, decentralized the governance and administration of health services to local government units. The structure includes having the Department of Health (DoH) setting national health policy agenda, with provincial governments managing tertiary level health services, and municipalities managing the delivery of primary and preventative care through rural health units and barangay health stations. In urban settings city health offices also manage barangay health stations within their boundaries.  At the operational level, Barangay workers may be employed by multiple levels of government and perform roles across different disease programmes depending on the needs of the barangay. | **Bell et al.,** 2001**, quantitative non-randomized study**  1. To assess the reliability and acceptability of immunochromatographic tests (ICT tests) in a remote area of the Philippines where volunteer CHWs receive limited training and support to diagnose and treat malaria  2. To compare the efficacies of remote symptom-based diagnosis of malaria, rapid diagnostic tests and microscopy in an area of low endemicity in the Philippine.  Only reported outcomes that relate to the functionality of Barangay workers were extracted as follows:  **Malaria and non-malaria related outcomes**  None  **Programme uptake and performance**  1. ICT tests were well accepted by BHWs  2. Similarity in ICT test results and slide concordance between the two trials (trial 1 by researchers and trial 2 by BHWs) show that BHWs were achieving ICT results of similar accuracy to those of the researchers. |
|  | **Berg et al,** 2012, **Case Study**  Focusing on three case studies in the Philippines, this study investigates the management of vector control systems at different levels of public administration, and describes the lessons that could be learnt about operational efficiency and sustainability of vector control strategies based on the decision-making conditions and integration of various programmes and/or health service providers (barangay health workers) that occurs at the varying administrative levels in the three case studies.  **Malaria related outcomes**  In one case study (Cagayan Valley), establishment of functional microscopy centres and RDT sites in remote areas and integration with existing community mechanisms (such as involving midwives) improved the quality of malaria detection and diagnostic services. The area observed a decline in confirmed malaria cases, predominantly pf, of 490 cases in 2005 to zero in 2011.  **Non-malaria related outcomes**  None  **Programme uptake and performance**  None |
|  | **Dodd et al,** 2021, **qualitative study**  To explore the experiences of CHWs and administrators of CHW programmes to examine individual-level and structural factors influencing the operations of CHWs across different settings.  **Malaria and non-malaria related outcomes**  None  **Programme uptake and performance**  None |

**S2.3 Extraction on strategies to ensure sustainability and factors on effective implementation**

| **Programme** | **Programme funding** | **CHW compensation** | **Training** | **Supervision** |
| --- | --- | --- | --- | --- |
| **AFG**  Community Health Workers (CHW) | Major donors (funded through partnership agreement/contracts with NGOs to deliver health services): 1. World Bank 2. US Agency for International Development 3. European Union   Smaller grants from: 1. Global Alliance for Vaccines and Immunizations (GAVI) 2. Global Fund to Fight AIDS, Tuberculosis and Malaria (Global Fund) 3. Japan International Cooperation Agency (JICA) | None (CHWs are volunteers) but they received in-kind supports from their communities. The MoPH has recently had success with performance-based cash incentives for CHWs providing care and financial incentives to encourage mothers to attend ANC and PNC, and delivering their child at a health facility.  CHW expenses for meetings and training are paid but survey showed that some CHWs do not receive travel expense or allowance from attending training courses with almost 50% reporting not being compensated for travel or food/other allowances when attending training.   For TB programme specifically, CHWs get compensated for accompanying suspected TB case to facilities. An award of US$ 100 per province per quarter were also offered to best performing CHWs in the TB programme.  Some honorarium is given to CHWs for supporting activities during National Immunization days and polio campaigns, among others. | Induction training: 3-4 months (3 modules/class with 3 classes)  Practical training: 1 month  Tool: training package and pictorial job aids | Supervisory visit at health post.  Monthly meeting at health facility.  Supervisor: full-time Community Health Supervisor (CHS) employed by government |
| **BNG**  Shasthya Shebika (SS) and Shasthya Komi (SK) | Not specified in text | SS are given small loans to establish revolving funds, which may be used to create income by purchasing and then reselling health products at a small mark-up.  SK work full-time and receive a monthly salary of US$190. | Skills-based trainings: 1. Malaria microscopy for lab technician 2. Case management and reporting documentation for staff  3. Malaria diagnosis and treatment for health workers | Not specified in text |
| **IND-I**  Accredited Social Health Activists (ASHA) | Funding for ASHA compensation varies across states:  1. Incentives paid through various national health programmes, or through untied funds from the Village Health Sanitation and Nutrition Committee, or specific state health department depending on the state 2. Ministry of Health and Family Welfare funds US$ 163 per ASHA (as of 2006) which excludes ASHA worker remuneration derived from  budgets of various other MoHFW initiatives 3. State funding  Malaria funding: 1. GFATM funded malaria work in northeast states (Round 9 during 2010-2015) 2. World Bank funded malaria work in other high endemic states (2008-2013) | ASHAs are honorary volunteers but are provided:  1. Compensation for their time in specific situations (training attendance, monthly reviews, other meetings)  2. Offered incentives under various National health programmes (cataract, family planning DOTs, malaria slides, support in water and sanitation programme).   The fixed performance or activity-based incentives provided for their services averaging US$ 42-56 per month for over 64 activities, such as: 1. US$ 9 for facilitating an institutional delivery 2. US$ 2.50 for facilitating a child’s completion of immunizations 3. US$ 28-29 per month for satisfactory performance of routine tasks - (unknown amount) incentives for malaria and TB works  Some states also offer other awards such as  1. Fixed monthly honorarium from state funds  2. Cash awards for the best-performing ASHAs  3. Recognition in newsletter and radio programs  4. Provision of bicycles for all ASHAs  5. Career development opportunities by giving scholarships to study nursing. | Induction training: 8 days  Skill-based training: 20 days  Additional training: 15 days/year  Refresher training: 10 days/year | Monthly meeting  Supervisor: ASHA facilitator |
| **IND-II**  Village Malaria Worker (VMW)  Accredited Social Health Activists (ASHA)  Auxiliary Nurse Midwife (ANW)  Multipurpose Health Worker (MPW) | Not specified in text | ASHAs are reported to receive an average of 12,521 INR according to a survey  Some ASHAs and ANWs were reported to receive incentives for malaria diagnosis and treatment | Induction training: 4-5 days  Refresher training: yearly (for field staff with minimal passing threshold of 70%)  Training conducted with ASHA, Auxiliary Nurse Midwives (ANM), Multipurpose Health Workers (MPW) in batches of smaller than 40 people | Supervisory visit at village by Malaria Field Coordinators (MFC).  Monitored by programme and district officer.  Supervisors:  1. MFC, programme officer, and district officers  2. ASHA sahayogi are ANM assistants who work with MPWs at health centers and manage up to 5 ASHAs  3. MPWs and ANMs are supervised by Lady health visitors and MPW supervisors who report to Block Medical Officer |
| **IND-III**  Mitanins (female health volunteers) | Not specified in text | Mitanin receives incentives based on the nationally prescribed incentives schedule for ASHAs for each activity they perform (there are no ASHAs in Chhattisgarh) | Skill-based training: 2 days on malaria control and surveillance | Supervisor: Malaria Surveillance Workers (MSW) |
| **IND-IV**  Village Health Guide (VHG) | Funding for VHG activities were derived from the government, with additional funds intended to be raised from communities and health system personnel, although it is unclear if this was achieved.   At programme inception, the central government fully funded the programme but by the end of the 1970s half of the cost was covered at the state level. Although the report did not identify external donors, there is indication that the government’s financing of the program was heavily dependent on external aid. | VHGs received a salary of approximately US$ 24 per month (2019 currency), but received a higher rate of about US$100 (2019 currency) during their training. VHGs also received rs 50 worth of medicines and supplies from the Public Health Center (PHC) to dispense to their patients.  VHGs are paid through PHCs. | Not specified in text | Not specified in text |
| **IND-V**  Multipurpose Health Workers (MPW) | Not specified in text | MPWs were provided financial compensation for performing activities under vertical programmes (National Malaria Elimination Programme) | Not specified in text | Not specified in text |
| **IDN-I**  Midwives | 1. Global Fund (malaria programme that also supports integrated maternal health and immunization) 2. USAID via UNICEF (initial implementation) 3. Co-financed by national budget and local budget | Not specified in text | Not specified in text | Not specified in text |
| **IDN-II**  Kaders | Funded by the Ministry of Home Affairs, Ministry of Health, Family Welfare Movement ((Pemberdayaan Kesejahteraan Keluarga or PKK) and PKMD | Text specified as employed based on volunteerism | Kaders are trained through puskesmas or community health clinics | Not specified in text |
| **IRN**  Behvarz | The Government of Iran provides all of the financing for health houses and health posts where the Behvarz are assigned. Funding is drawn from a combination of public government funding and the insurance system. | Behvarzs are officially salaried Ministry of Health and Education staff, and receive a monthly government salary of approximately US$350 per month plus an additional performance-based formulation of payment for incentive.  For all Public Health Center employees, including Behvarzs and Moraghebe-salamats, incentives may also be provided for good performance. | 2-year skill-based practical training (only 6 months in length for degree holders) | Periodical supervision (unspecified)  Supervisor: physicians and health centre staff |
| **KHM-I**  Village Malaria Workers (VMW) | 1. VMW programme is financed by the Global Fund through the National Centre for Parasitology, Entomology and Malaria Control (CNM) 2. Medications for extended roles are financed by the WHO and three international donors via the Ministry of Health | Unspecified whether consistent compensation is provided within text, but indicated that since 2019, a majority of VMWs who participated in scheduled meetings of the programme received payment for participation through electronic payments | Induction training: 2 days  Refresher training: once a year or with introduction of new roles | Supervisory visit at village (twice a year)  Monthly meetings at health centres  Supervisor: CNM staff |
| **KHM-II**  PSI trained VMW and recruited Mobile Malaria Workers (MMW) | PSI was contracted to conduct the study with funding from the Gates Foundation Project, by Bill & Melinda Gates Foundation.  PSI provided the recruited providers in the GEMS project with: RDTs, referral cards, antimalarial drugs, Orasel (vitamin supplements), and condoms. | Incentive of US$20 per month was provided if workers performed a minimum number of tests and reported cases | PSI provided training, details unspecified. | Not specified in text |
| **KHM-III**  Village Malaria Workers (VMWs)  Lab technicians | The pilot study was funded by the Joint Global Health Trials MRC/Wellcome  The programme overall is funded by the Global Fund and CNM | VMWs receive minimal monthly incentives, with additional incentives for each new cases of malaria when diagnosed, referred and followed-up within the community | 33 participants in this study received initial training on background information on *P.vivax*, radical cure, the role of G6PD enzyme and the use of biosensors and interpretation of the results in small groups. A practical session was also provided. | Participants remain in regular contact with study coordinators via phone. |
| **KHM-IV**  Village Malaria Worker | The study was sponsored by the US Defence Threat Reduction Agency Joint Science and Technology Office (DTRA-JSTO). | Not specified in text. | Training to perform RDT test provided prior to conducting the competency test. | Not specified in text. |
| **KHM-V**  **Village Malaria Workers** | This study is part of operational research titled “Sustaining village health worker programmes with expanded roles in the GMS” supported by the Global Fund to Fight AIDS, Tuberculosis and Malaria. | Amount not specified in text, but mentions VMWs reporting that their incentives are too low to carry out their roles, especially when there is a need to travel. | VMWs are trained to provide malaria diagnosis, treatment, referral, surveillance, and preventive activities. For the expanded roles, VMWs were also trained to use new test kits (dengue antigen–antibody RDTs, combined malaria/CRP tests, and multiplexed biosensors), collect data electronically, and on health education packages (hygiene and sanitation, disease surveillance and first aid, management of mild illness, and vaccination and antenatal care). | The new diagnostics were offered to VMWs by AHEAD under the supervision of CNM and supported technically by MORU. |
| **LAO-I**  Village Health Volunteers (VHV) and Village Malaria Workers (VMWs) | 1. Global Fund (routine training, supervision and malaria commodities) 2. United Nations Office for Project Services 3. Civil society organizations  Many expanded roles were being funded by external malaria funding  For PSI-GEMS sub-project, PSI provided the providers in the GEMS project with: RDTs, referral cards, antimalarial drugs, Orasel (vitamin supplements, and condoms. | VHVs are volunteers receiving no regular compensation for their work.  VMWs receive a 19 USD monthly stipend (12 USD incentive and 7 USD transportation payment) (Napier, 2021).  VMWs are provided 13 USD for helping with vector control activities, disease surveillance, case management using RDTs and ACT, and referral of patients with severe malaria to the health centres they are linked with (Liverani, 2024) | Type and duration not specified in text but training is provided by provincial staff. | Due to limited resources, provincial and district staff were unable to conduct regular field-based supervision. VHVs received supervision during monthly meetings at health centres during which VHVs can submit reports, get stock replenishment, mentorship, and payments. |
| **LAO-II**  Shop-based volunteer malaria workers (sVMW) and PSI/Laos supported private providers | Not specified in text | Not specified in text | Not specified in text | Not specified in text |
| **LKA**  Health Volunteers (HV) | Government does not provide funding for the programme, and various international donors were cited as providing financial support, although it is unclear how much and for what aspects of the programme. UNICEF is cited to have provided training support for health volunteers, but coverage is limited. | Volunteers are non-salaried, nor are their supervisor/trainers (Public Health Midwives) | Training: 3-month course for HVs in settlement areas and 5-day course for HVs in non-settlement areas | Monthly meetings with public health staff for HVs in settlement areas and informal supervision for HVs in non-settlement areas |
| **MMR-I**  Sun Primary Health (SPH) providers | Not specified in text | Financial incentives based on monthly performance (did not specify further) | Not specified in text | Not specified in text |
| **MMR-II**  Village Health Workers (VHW) | 1. Three Millennium Development Global Fund 2. Global Fund to fight AIDS, Tuberculosis and Malaria  3. Medical Action Myanmar | 5000 Kyats or 4 USD fixed monthly incentive, plus 500 kyats (0.4 USD) for every patients tested | VHWs received training in line with the curricula of the NMCP and the National TB programme, provided by MAM mobile medical teams.  Induction training:  1. 2 days for malaria  2. 1 day for TB  On-the-job training: monthly for malaria and TB | VHWs were managed by MAM mobile medical teams that visited the community monthly to supervise, collect routine data, and provide supplies and training for VHWs (Zaw, 2023). |
| **MMR-III**  Malaria Volunteers (MV) | Funding not specified for pilot period.  Comic Relief funded post-pilot period implementation of the programme by Malaria Consortium. | Indicated that MVs receive financial and in-kind support (not specified further).   Some ICMVs stated that they felt that in recognition of their increased workload and responsibility they should receive a higher cash incentive. Recommendation said MOHS could consider increasing incentives for ICMVs to match this expanded workload, in addition to financial support for transportation and case referral, and continuous medicine and equipment support (e.g., ORS, multivitamin, first aid kit, BP cuff) for sustainability.  Not sure if implemented or not "they will receive a cash incentive of MMK 50,000 each quarter" p20 | Induction training by iCCM trainers  Refresher training by supervisors | Not specified in text |
| **MMR-IV**  Integrated Community Malaria Volunteers (ICMVs) | Donor not specified, but indicated that donors provided monetary incentive for volunteers as well as their kits, along with replenishment of medicines and supplies on a monthly basis  Government funding provides for health facility infrastructure, payment of government staff salaries, and some supplies | Financial (transportation, case referral, among others) and in-kind (continuous medicine and equipment support) provided but the amount did not match the increasing roles and responsibilities as part of the Integrated Community Case Management Volunteer model.  One study specified that ICMVs were supplied with mobile credit of between 5000 MMK (approx. 3.5 USD) per three months and 10,000MMK (approx. 7 USD) | Malaria induction training: 2 days in-classroom followed by monthly on the on the job training by NMCP and other NGOs  Skill-based training: 5-6 days for new roles as ICMV  Refresher training: intended to be done annually but frequency depends on donor support | Supervisor: NGO staff, government health staff e.g. malaria staff at township health department |
| **MMR-V**  Integrated community health worker (ICMW) | Not specified in text | Unlike ICMVs. ICMWs are compensated; the amount was not specified in text. | Training in ICMV provided to malaria posts in 2019. The training included an initial 5 day training programme; based on biannual knowledge and skills assessment, refresher trainings may be provided to malaria posts when needed. | Routine monitoring and evaluation visits conducted, |
| **MMR-VI**  Better Health Together project’s Integrated community health volunteer (ICMV) | Better Health Together is funded by the Three Millennium Goal Fund (3MDG) | Fixed honorarium of about 17 USD per month. | Community Partners International (NGO) trains Ethnic Health Organizations (EHOs) in ICMV guidelines. Then EHOs offer multiplier training offered to volunteers, with each training lasting nine days (7 for ICMV guidelines and 2 for recording and reporting formats). EHOs conduct refresher training annually. | Continuous supervision by EHOs. |
| **MMR-VII**  Backpack Health Worker Team (BPHWT) | Donor not specified, but reported on their income including from major donors between 2002-2009 in their 10 year report. | Not specified in text | Utilizes training of trainers model whereby BPHW are trained to provide training to local CHWs | Not specified in text |
| **MMR-VIII**  Mobile Obstetric Maternal Health Workers (MOM) Project | The MOM Project is funded by grants from the Bill and Melinda Institute for Population and Reproductive Health at the Johns Hopkins Bloomberg School of Public Health, Global Health Access Program/Planet Care, the Hussman Foundation, and the Foundation for the People of Burma. | Remuneration for all types of workers is determined based on the policies of the ethnic health organizations. MHWs and lay health workers receive a monthly stipend for their fieldwork, along with a monthly food allowance. In some instances, this food allowance is merged with existing clinic funds to cover meals for all health workers. TBAs receive a per diem allowance during both initial and follow-up training sessions. Survey team members are similarly compensated during their field survey period, typically lasting two months. | In Phase I, recruited MHWs were trained over 6-month long specialized program tailored for the project. The training was delivered by a physician and senior medics from the reproductive health department at Mae Tao Clinic, with technical guidance from the Global Health Access Program (GHAP) and the Johns Hopkins Center for Public Health and Human Rights.  Prior to MOM training, all workers participated in a minimum of four months of basic health training, with the majority completing a six-month course. Most workers had over 2 years of fieldwork experience, and all workers achieved at least a sixth standard of education. | The project incorporates regular information-sharing and training workshops. MHWs are required to return to Thailand annually, while team leaders attend every six months. These follow-up sessions involve reviewing clinical work in the field, supplemented by practical training and supervision at the Mae Tao Clinic's reproductive health department. The workshops also serve as a chance to coordinate logistical arrangements for resupplying areas, review data collection tools, and make necessary updates. Periodic reviews of the progress and capabilities of MHWs enable the addition of new training modules and interventions to the existing platform. |
| **NPL**  Rapid Response team or Female community health volunteers (FCHVs) | Not specified in text but the incentive paid to FCHVs are covered by the Nepalese government | FCHVs receive an incentive of Rs. 400 per day per activity as transportation cost. | FCHVs were trained to build their knowledge and skills on how to distribute Iron and folic acid (IFA) supplements, and counsel women on the benefits of IFA and on taking supplements for pregnant women. | FCHVs were supervised by health workers and participated in monthly meetings at the nearest health facility. During the monthly meetings, they also report on their activities and received supplies. |
| **PAK**  Lady health workers (LHWs) | Between 1994 and 2002 LHW programme budget was at US$ 155 million comprised of:  1. Government of Pakistan (largest funder) 2. Donors (not specified; about 11% of programme budget)  Additional note regarding funding: 1. Program has been underfunded since its inception 2. Between 2003 and 2008 LHW programme budget was estimated at US$ 356.6 million  3. Overall, the program spent approximately US$ 570 per LHW per year between 2003 and 2008  4. Approximately 70% of LHW programme costs are for LHW stipends, drugs, and contraceptives, and an additional 4% are for training 5. LHW salary costs increased 31% between 2003 and 2008, leading to a reduction in other expenditures, especially for LHW kit supplies. | LHWs receive a salary of approximately US$ 180 per month for their services and are not supposed to engage in any other paid activity, although some do.    Additional note on compensation: 1. LHW stipend is often the only source of family income and is a critical source of family support 2. Salaries are paid monthly into the LHWs’ personal bank accounts, but delays are common  3. LHWs are also paid a small amount of money during the initial training for the first three months followed by almost half of the regular salary per month 4. LHWs can earn additional income from selling contraceptives to their clients 5. LHWs also have additional incentives such as professional advancement and promotions | Basic training: 3 months  On-the-job training: 1 year  LHWs are appointed after completion of OTJ training  Refresher training: once after previous training  Training designed by Federal Project Implementation and conducted by provincial and district units | Monthly visit/meeting  Supervisor: LHW supervisor |
| **PNG-I**  Volunteer Community-based Distributors (CBDs) | Not specified in text | Not specified in text | Not specified in text | Not specified in text |
| **PNG-II**  Marasin meri or Marasin man (Village Health Volunteer or VHV) | Not specified in text | Not specified in text | 1-month initial training (for what activities not further specified). | Supervision provided by nursing officer and health workers stationed at the health center. |
| **PHL**  Barangay Health Workers (BHW) | Barangay health worker programme draws funding from multiple sources. At the operational level they are managed through Barangay health posts under local government units (city health offices or municipal health offices). Other partners and donors fund community-based malaria control programmes; for example, the Kilusan Ligtas Malaria (KLM) (Tagalog: Movement Against Malaria) is also funded Japan International Cooperation Agency and the Global Fund (through the Pilipinas Shell Foundation, Inc.), and the Agusan del Sur Malaria Control and Prevention Project (ADS-MCP) which received funding from the Australian National Health, Medical Research Council, and AusAID. | Type and amount of compensations for BHWs vary based on areas/city they are in. In higher income barangays, BHWs may receive a regular salary but in others they may be appointed on a voluntary basis, receiving only a small stipend for their work | The Republic Act no. 7883 (1995), also referred to as the Barangay Health Worker Act, indicates that Barangays receive 6 months of training and undergo a post-training accreditation process implemented by the DOH, local governments, and the Civil Service Commission. Across the country, several common characteristics of training include some level of basic training, yearly ‘refresher’ trainings, and monthly ‘update’ meetings. Components of the training (length, fees paid to attend training, providers of basic training, and the availability of supplementary training) varied between regions, and sometimes even within barangays. | Not specified in text |
